# Well-Defined Synthetic Copolymers with Pendant Aldehydes Form Biocompatible Strain-Stiffening Hydrogels and Enable Competitive Ligand Displacement

**DOI:** 10.1021/jacs.4c04988

**Published:** 2024-08-20

**Authors:** Ivo A. O. Beeren, Francis L. C. Morgan, Timo Rademakers, Jurica Bauer, Pieter J. Dijkstra, Lorenzo Moroni, Matthew B. Baker

**Affiliations:** † Department of Instructive Biomaterials Engineering, MERLN Institute for Technology-Inspired Regenerative Medicine, Maastricht University, 6229 ER Maastricht, The Netherlands; ‡ Department of Complex Tissue Regeneration, MERLN Institute for Technology-Inspired Regenerative Medicine, Maastricht University, 6229 ER Maastricht, The Netherlands

## Abstract

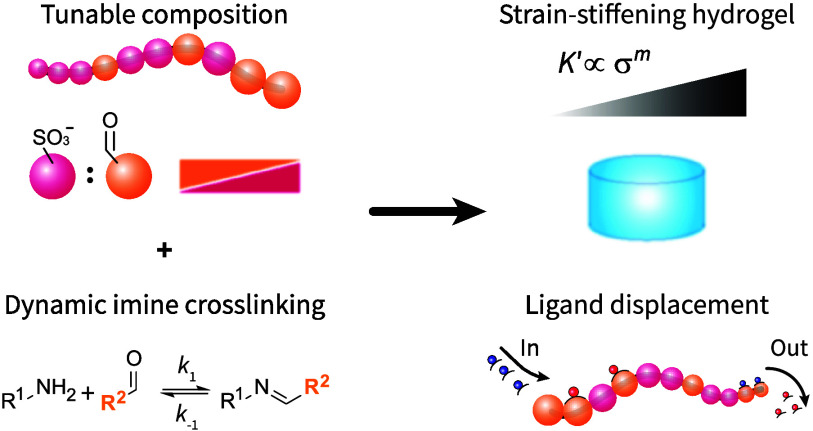

Dynamic hydrogels are attractive platforms for tissue engineering and regenerative medicine due to their ability to mimic key extracellular matrix (ECM) mechanical properties like strain-stiffening and stress relaxation while enabling enhanced processing characteristics like injectability, 3D printing, and self-healing. Systems based on imine-type dynamic covalent chemistry (DCvC) have become increasingly popular. However, most reported polymers comprising aldehyde groups are based on either end-group-modified synthetic or side-chain-modified natural polymers; synthetic versions of side-chain-modified polymers are noticeably absent. To facilitate access to new classes of dynamic hydrogels, we report the straightforward synthesis of a water-soluble copolymer with a tunable fraction of pendant aldehyde groups (12–64%) using controlled radical polymerization and their formation into hydrogel biomaterials with dynamic cross-links. We found the polymer synthesis to be well-controlled with the determined reactivity ratios consistent with a blocky gradient microarchitecture. Subsequently, we observed fast gelation kinetics with imine-type cross-linking. We were able to vary hydrogel stiffness from ≈2 to 20 kPa, tune the onset of strain-stiffening toward a biologically relevant regime (σ_c_ ≈ 10 Pa), and demonstrate cytocompatibility using human dermal fibroblasts. Moreover, to begin to mimic the dynamic biochemical nature of the native ECM, we highlight the potential for temporal modulation of ligands in our system to demonstrate ligand displacement along the copolymer backbone via competitive binding. The combination of highly tunable composition, stiffness, and strain-stiffening, in conjunction with spatiotemporal control of functionality, positions these cytocompatible copolymers as a powerful platform for the rational design of next-generation synthetic biomaterials.

## 1 Introduction

The development of water-soluble polymeric biomaterials and hydrogels that capture native ECM bioactivity and mechanical properties is an open challenge. While covalently cross-linked hydrogels have been successful at mimicking select ECM properties such as stiffness, they fail to capture the time-dependent dynamic characteristics of the ECM including stress relaxation or spatiotemporal ligand presentation.^1−3^ Dynamic systems based on host–guest chemistry,^4,5^ supramolecular self-assembly,^6−8^ and dynamic covalent chemistry (DCvC)^9−13^ have emerged as important tools for designing next-generation biomimetic materials. Their inherent potential to mimic dynamic interactions in the native ECM arises from the reversible nature of noncovalent interactions and reversible covalent bonds.

Chaudhuri et al. were one of the first to demonstrate that substrates with a similar stiffness, but differences in stress relaxation, affected cell spreading and differentiation.^14,15^ More recently, the time scales of the reversible cross-links or supramolecular interactions have been directly linked to the control of the viscoelastic character of hydrogels, which is ultimately vital for the control of cell fate.^16−19^ Reversible chemistries can also provide opportunities for temporal control over the presentation as well as the release of the bioactive molecule(s). For example, Boekhoven et al. performed pioneering work on displacing bioactive epitopes in hydrogels using competitive host–guest binding to exert control over the adhesive response of fibroblasts.^20^ Similarly, Zhan et al. used boronic acid esters to trigger the release of biological epitopes either using a competitor or by exploiting pH-dependent binding affinities.^21^

Imine-type DCvC (reversible reaction between an aldehyde and a terminal amino group) has become increasingly popular for tuning the viscoelastic time scales of soft biomaterials.^22^ Different types of cross-linkers such as oximes, semicarbazones, and hydrazones allow the engineering of hydrogel properties (i.e., stiffness, stress-relaxation, (bio)printability) based on the inherent differences in molecular equilibrium and rate constants.^22−24^ Notably, the chemical design space of such dynamic polymeric hydrogels is limited to telechelic synthetic systems or pendant/main-chain modifications of natural polymers. While some examples of synthetic telechelic systems can be found,^22,25−27^ there remain remarkably few examples of water-soluble, biocompatible, synthetic polymers with pendant aldehyde groups.^28,29^ On the other hand, the facile chemical modification of natural biopolymers with aldehydes via oxidation makes them popular candidates for DCvC.^9,10,23,24,30,31^ However, the inherent heterogeneity and dispersity of polysaccharides, in addition to their propensity to tie up aldehydes as hemiacetals,^32^ ultimately can lead to limited control over composition and batch-to-batch variability. In contrast, synthetic polymers can circumvent some of these drawbacks due to their well-defined chemical composition, molecular weight, and structural simplicity.

Modern polymerization techniques such as reversible addition–fragmentation chain-transfer (RAFT) or atom transfer radical polymerizations enable the synthesis of low dispersity polymers.^33^ However, polymerization in the presence of free aldehydes is difficult due to their high reactivity toward nucleophiles,^28^ aldol condensation,^34,35^ and propensity for radical harvesting.^36^ There are some examples of the successful polymerization of hydrophobic monomers or mixtures of hydrophobic and hydrophilic monomers, yet these polymers are not easily amenable to hydrogel formation and thus biological applications.^37−40^ Despite these few examples, most studies that reported water-soluble polymers comprising pendant free aldehydes via controlled radical polymerization techniques, used monomers with protected aldehydes.^41−47^ Current examples of the protection strategy typically require multiple complex synthetic steps to finally yield a water-soluble polymer. Ideally, a straightforward synthetic strategy could provide a path forward toward hydrogel and biomaterials formation.

**Figure 1:**
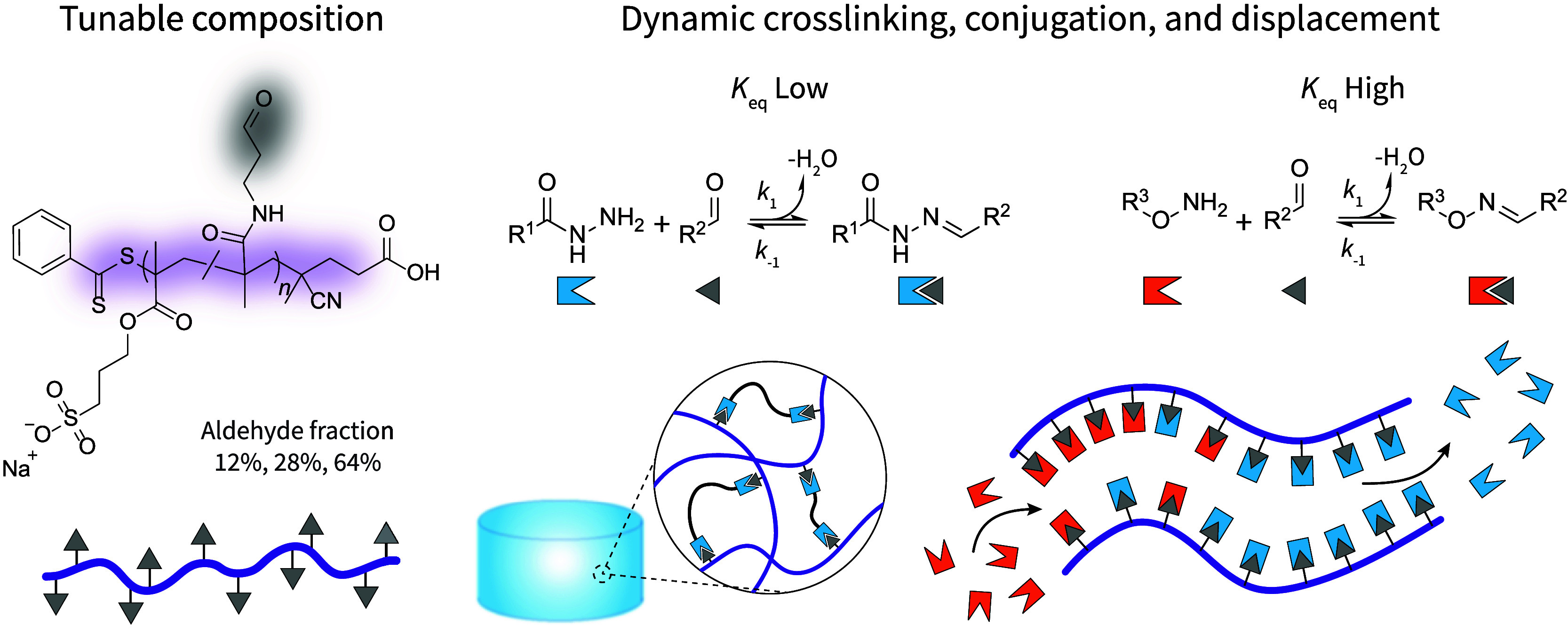
Synthetic copolymer platform to create well-defined, multifunctional hydrogels. By strategically choosing monomers that ensure both aqueous solubility (sulfonate groups) and strategic reactivity (aldehyde groups), we created copolymer hydrogels with dynamic cross-links and polymers with bioconjugate groups that can be released through competitive binding.

Herein, we present the controlled RAFT copolymerization of *N*-(3,3-diethoxypropyl)-methacrylamide (**DEPMAm**) and 3-sulfopropyl methacrylate potassium salt (**SM**) to obtain a water-soluble aldehyde containing polymer. We obtained “blocky”^48^ gradient copolymers with a tunable pendant aldehyde fraction, depending on the monomer feed ratio (Figure 1, left). These copolymers cross-link rapidly with homobifunctional poly(ethylene glycol) dihydrazides (**PEG-HZ**), and exhibit strain-stiffening behavior, which has only been observed in a few synthetic systems.^49−51^ To test the ability of our synthetic system to mimic the evolving ligand presentation of native ECM, we leverage the reversible nature of the dynamic covalent bonds to demonstrate competitive ligand displacement (Figure 1, right) via fluorescence resonance energy transfer (FRET). These synthetic hydrogels also display excellent cytocompatibility–a prerequisite for biomaterials applications. Due to the straightforward synthetic procedure and modular hydrogel formation, we believe that this novel synthetic platform is accessible to the broader biomaterial community.

## 2 Results and Discussion

### 2.1 Synthesis and Characterization of Poly(3-Sulfopropyl Methacrylate-*co*-*N*-(3-oxypropyl) Methacrylamide) (pSM-*co*-OMAm)

**Scheme 1:**
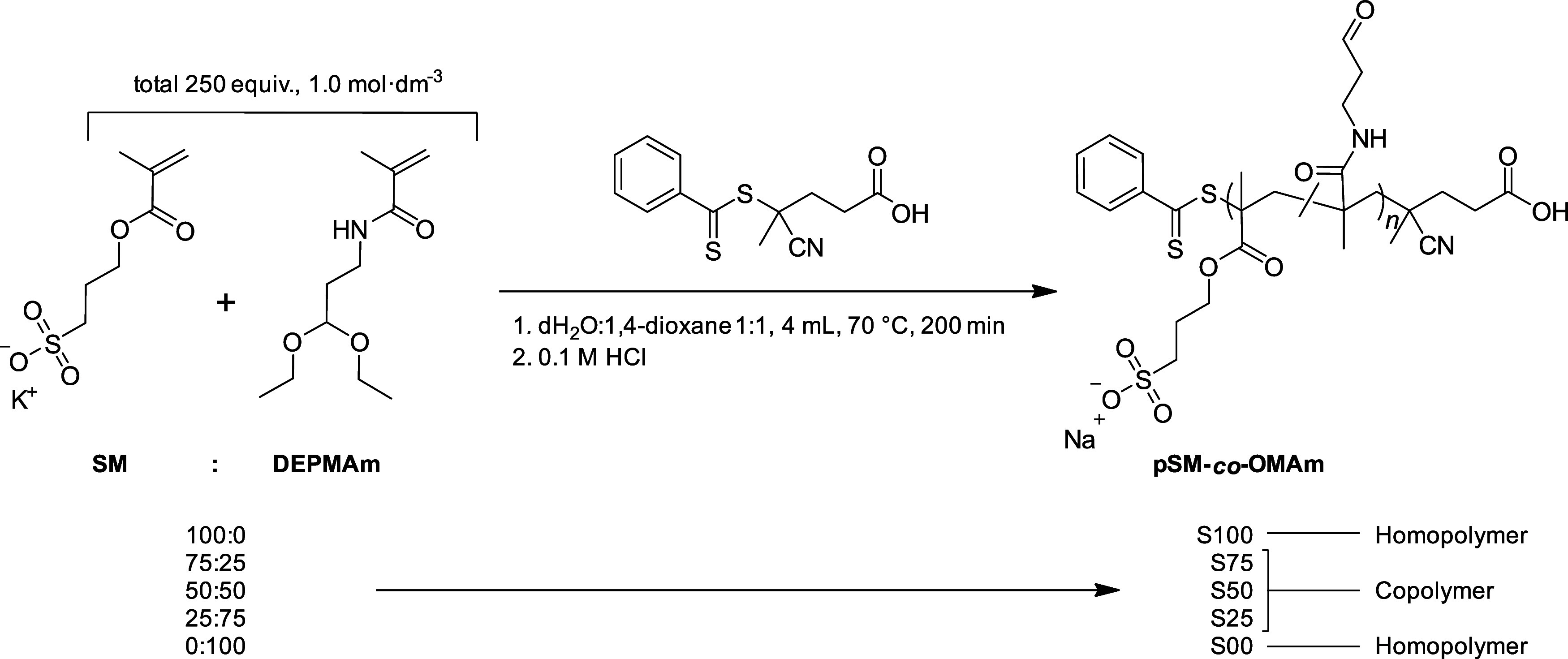
Synthesis of a Small Library of Poly(3-sulfopropylmethacrylate-*co*-*N*-(3-oxypropyl) Methacrylamide) (pSM-*co*-OMAm) Copolymers and Associated Homopolymers Different molar feed ratios for the reversible addition–fragmentation chain transfer (RAFT) copolymerization of 3-sulfopropyl methacrylate potassium salt (**SM**) and *N*-(3,3-diethoxypropyl)methacrylamide (**DEPMAm**) enable control over the final aldehyde content of the resulting (co)polymers.

To introduce pendant aldehyde groups on polymer chains by RAFT polymerization, monomer **DEPMAm**, containing a diethoxy-protected aldehyde, was selected. The monomer was synthesized by methacrylation of 1-amino-3,3-diethoxypropane adapted from a previously reported synthetic method (Figures S1–S3).^52^ To ensure the aqueous solubility of a polymer for the preparation of hydrogels, 3-sulfopropyl methacrylate potassium salt (**SM**) was selected as a comonomer. RAFT (co)polymerizations at different comonomer feed ratios ranging from 100 mol % **SM** to 100 mol % **DEPMAm** with 25 mol % increments were performed using 4-cyano-4-(phenylcarbonothioylthio)pentanoic acid (CPPA) as a chain transfer agent (Scheme 1). A mixed solvent system of dioxane and distilled water (1:1, *v*/*v*) was used to obtain a homogeneous solution during the polymerization. Following polymerization, crude reaction mixtures were dialyzed against 0.1 M HCl to ensure the conversion of acetal groups to aldehydes. At all feed ratios, except for the homopolymerization of **DEPMAm** (Figures S4, S5 and Table S1), a water-soluble polymer was obtained. In **Discussion S1**, we elaborate on the side reaction observed during **DEPMAm** homopolymerization that could explain the insolubility of the purified product. Thus, throughout this manuscript, we have denoted the prepared (co)polymers according to the mole fraction of **SM** monomer in the initial feed ratio, namely, **S25**, **S50**, **S75**, and **S100**.

**Figure 2:**
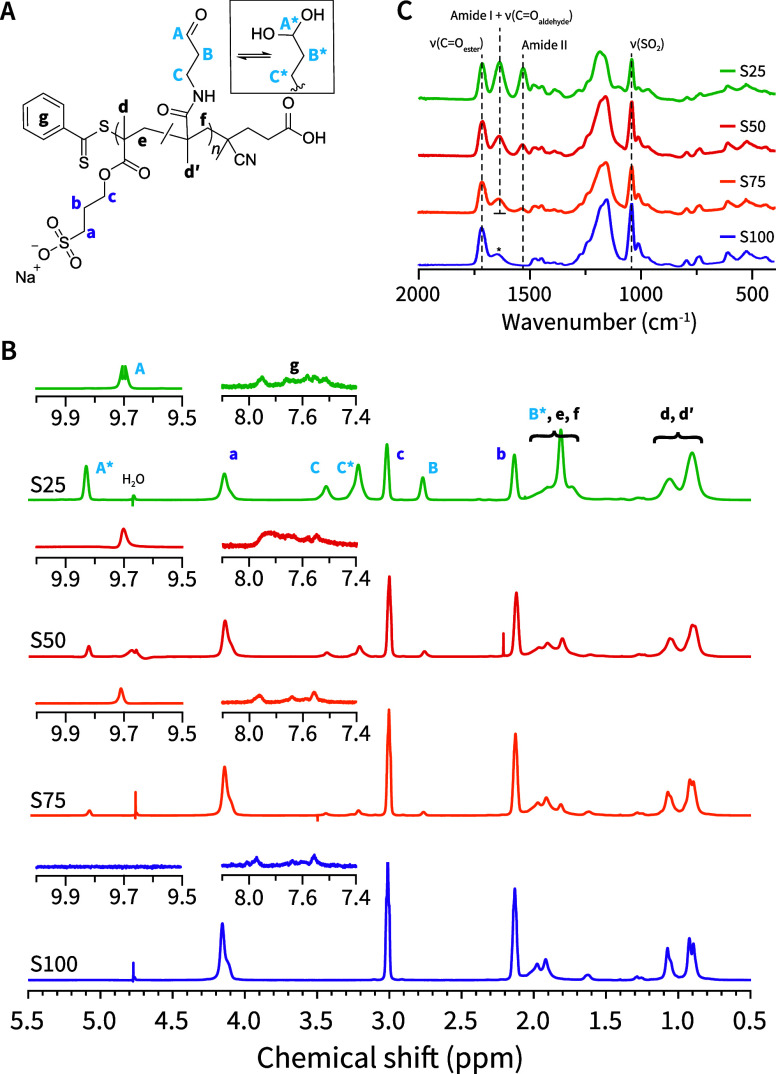
Structure and characterization of poly(3-sulfopropyl methacrylate-*co*-*N*-(3-oxypropyl) methacrylamide) (pSM-*co*-OMAm) (co)polymers. (A) Polymer structure showing the aldehyde in equilibrium with its hydrate. (B) ^1^H NMR (700 MHz, D_2_O) spectra of purified **pSM-***co***-OMAm** copolymers (**S25–S75**) and the poly(3-sulfopropyl methacrylate) homopolymer (**S100**) with peak assignments. Note the presence of the free aldehyde state (A, B, C) and its hydrate (A*, B*, C*). In Figure S14, the **S25** spectrum is included as a representative example to indicate that the integral ratio of a:b:c is ≈1:1:1, and (A + A*):(B + B*):(C + C*) is ≈1:2:2. (C) ATR-FTIR spectra of purified **S25–S100**. The amide I and aldehyde carbonyl stretch (1637 cm^–1^), amide II bands (1537 cm^–1^), sulfonate (1041 cm^–1^), and ester (1714 cm^–1^) peaks are denoted with dotted lines. The peak with an asterisk (*) in the **S100** spectrum does not align with the assigned amide I and aldehyde peak (1648 vs 1637 cm^–1^). The full ATR-FTIR spectra can be found in Figure S15.

Immediately apparent in the purified NMR spectra is the appearance of a free-aldehyde peak (≈9.7 ppm) and the absence of the acetal-protecting group (≈1.1 and 3.5 ppm, Figure 2). Analysis of the progress of the reaction by ^1^H NMR revealed that deprotection of the aldehyde groups took place to a large extent during the reaction (Figures S6–S9). This was unexpected, as the pH of the reaction mixture was ≈5, and diethyl acetals are typically stable under aqueous conditions at pH > 4.^53^ In looking to control the deprotection, we performed a small-scale copolymerization around neutral pH (≈6.5), yet these conditions did not prevent in situ deprotection nor influence the degree of **DEPMAm** incorporation (Discussion S2, Figures S10–S12 and Table S2). Encouragingly, we found a report of deprotection under conditions that are very similar to our reaction conditions. This report indicates that elevated temperature alone is sufficient for the deprotection of acyclic aliphatic acetals in aqueous–organic solvent systems.^54^ Interestingly, the in situ deprotection of **DEPMAm** did not appear to greatly affect the copolymerization of **S25–S75** under the initial reaction conditions. This result led to the hypothesis that polymerization of a monomer containing a free aldehyde group under the same reaction conditions may be possible. In the following, we attempted to deprotect the **DEPMAm** monomer under slightly acidic conditions (typical for acetal-protecting groups, Figure S13). We were unable to isolate *N*-(3-oxopropyl)methacrylamide) due to dominant side reactions (Discussion S2), and consequently, we could not explore the RAFT polymerization of this aldehyde-containing monomer. Overall, no postmodification steps were required to obtain the desired products, and we leveraged the simplicity of the procedure moving forward.

The ^1^H NMR spectra of the final (co)-polymers **S25–S100** are depicted in Figure 2B. The composition of each isolated and purified polymer was determined using the integral ratio of signals of free and hydrated aldehyde (9.71 and 5.10 ppm) to the signal of the methylene protons (4.16 ppm) adjacent to the sulfonate group (Figure S14). Results of this analysis showed that **S25**, **S50**, and **S75** contained 64, 29, and 12 mol % **DEPMAm**, respectively. Aligned with the trends in NMR, ATR-FTIR spectra of **S25–S100** displayed an increase in the amide II band (1537 cm^–1^) with increasing amounts of **DEPMAm** incorporation, along with a concomitant decrease in the symmetric SO_2_ stretch at 1041 cm^–1^ (Figures 2C and S15). These results indicated that **SM** is incorporated preferentially over **DEPMAm** into the copolymer under the applied reaction conditions. This observation is consistent with the reported higher reactivity of methacrylates compared to methacrylamides when CPPA is used as the chain transfer agent.^55^

Calculation of the molecular weight by NMR using the end-group proton signals of the chain transfer agent at 7.5–8.0 ppm led to inconsistent and overly large values (Table 1), likely due to partial hydrolysis of the CTA during extended exposure to aqueous conditions (dialysis).^56^ In contrast, GPC analysis showed *M*_n_'s ranging from 51.5 kg mol^–1^ for the **S100** to 43.5–34.5 kg mol^–1^ for the **S75–S25** copolymers, which align with the theoretical *M*_n_’s when adjusted for the total monomer conversion (Table 1 and Figure S16). Synthesis of the **DEPMAm** homopolymer afforded multiple low molecular weight species that we could not isolate (Discussion S1). The low dispersity for **S25–S100** (co)-polymers (≤1.20) is consistent with a controlled RAFT polymerization. Notably, we also attempted free radical copolymerization of **SM** and **DEPMAm**. Although the product by NMR appeared similar to the product of the controlled polymerization, we observed precipitation after 1–2 h and a broad bimodal molecular weight distribution (Discussion S2).

**Table 1:** Effect of Molar Feed Ratio on the Composition and Molecular Weight of pSM and pSM-*co*-OMAm

entry	*f*_SM_ (%)^a^	*f*_DEPMAm_ (%)^a^	conv. (%)^b^	*F*_Ald_ (%)^c^	*M*_n,theo_ (kg mol^–1^)^d^	*M*_n,NMR_ (kg mol^–1^)^e^	*M*_n,GPC_ (kg mol^–1^)	*Đ*^f^
**S100**	100	0	91	0	58.4	108	51.5	1.18
**S75**	76	24	64	12	57.3	95	43.5	1.14
**S50**	51	49	59	29	56.3	44	36.4	1.18
**S25**	25	75	65	64	55.2	60	34.5	1.20
**S00**	0	100	49	100	54.1	–f	8.5^e^	1.32

a The monomer molar feed ratio was determined by ^1^H NMR (DMSO-*d*_6_).

b The monomer conversion was determined by ^1^H NMR (DMSO-*d*_6_).

c The fraction of incorporated aldehyde units was determined by ^1^H NMR (D_2_O), except for **S00** whose solvent was DMSO-*d*_6_.

d The theoretical *M*_n_ was determined according to [M]_0_*p*(*f*_SM_ × *M*_SM_ + *f*_DEPMAm_ × *M*_DEPMAm_)/[CPPA] + *M*_CPPA_, where [M]_0_ is the initial total monomer concentration, and *M*_SM_ and *M*_DEPMAm_ are the molecular weights of **SM** and **DEPMAm**, respectively.^57^ The conversion (ρ) was set to 1, and *f*_SM_ and *f*_DEPMAm_ denote the initial feed ratios. The expression (*f*_SM_ × *M*_SM_ + *f*_DEPMAm_ × *M*_DEPMAm_) represents the feed ratio adjusted average molecular weight of the initial monomer composition.

e The *M*_n, NMR_ was determined using the integral ratio of CTA and backbone signals (see Materials and Methods, Section 4.10).

f The purified product was insoluble (Table S1). We present GPC data, taken from the crude reaction mixture at *t* = 200 min (Figure S4). The dispersity was derived from GPC.

Finally, after confirming the chemical composition and molecular weight of the (co)polymers, we assessed the physical characteristics of **S25–S100**. Thermogravimetric analysis (TGA) showed an initial 10% mass loss attributed to the loss of water absorbed or bound to the sulfonate groups followed by a plateau up to at least 250 °C (Figure S17). Differential scanning calorimetry (DSC) analysis of **S50–S100** did not exhibit melting transitions up to 200 °C, indicating that these polymers are amorphous. The **S25** showed a small apparent glass transition around 121 °C (Figure S18) which lies well outside of physiologically relevant temperatures for biomedical applications and is beyond the scope of the current investigation.

### 2.2 Polymerization Kinetics and Monomer Reactivity Ratios

**Figure 3:**
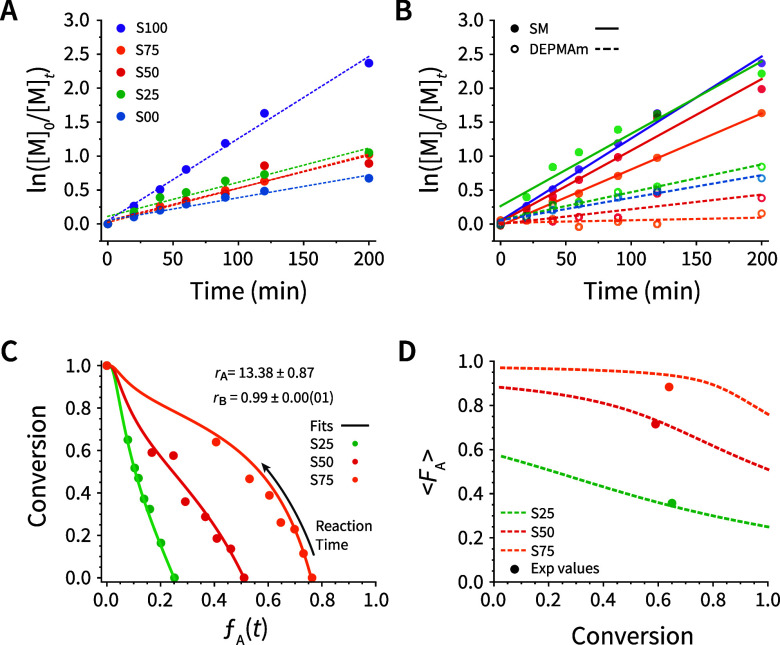
Reaction kinetics and reactivity ratios for the RAFT (co)polymerization of SM and DEPMAm. (A) Apparent first-order kinetics plot of global monomer conversion for homo- and copolymerizations. Here, [M]_*t*_ is the total monomer concentration at time *t*, and [M]_0_ is the total initial monomer concentration. These values were determined via ^1^H NMR using the methacrylate, methacrylamide, and backbone proton signals (see Materials and Methods, Section 4.15). (B) Apparent first order fits for **SM** (solid lines) and **DEPMAm** (dotted lines) individually in homo- and copolymerizations. (C) Plot of the global monomer conversion (via NMR) vs the real-time mole fraction of unreacted **SM** monomer, *f*_A_(*t*). The lines represent the fit of these data to the Meyer–Lowry model (eq 1), which was used to determine the reactivity ratios for each monomer. **SM** is denoted as **A** and **DEPMAm** as **B** for discussing reactivity ratios; *r*_A_ = *k*_AA_/*k*_AB_ and *r*_B_ = *k*_BB_/*k*_BA_. (D) A plot of the average **SM** fraction incorporated into the copolymer (< *F*_A_>, dotted lines) as a function of conversion (1 – [M]_*t*_/[M]_0_, eq S1) according to eq 2. The filled circles correspond to the final mole fraction of **SM** incorporated into the purified copolymers for the conversion obtained after 200 min.

The polymerization kinetics were investigated by evaluating the crude reaction mixtures over time via NMR and further analyzed to obtain insight into the microstructure of the prepared (co)-polymers. A plot of the natural logarithm of the total combined monomer concentration vs time yielded linear correlations at all feed ratios and revealed apparent first-order kinetics (Figure 3A). Almost independent of the amount of **DEPMAm** present (**S25**–**S75**), the apparent overall reaction rate constants showed similar values of ≈0.005 min^–1^ (Table 2). To better understand the reactivity of individual monomers, we then considered the apparent first-order kinetic profiles for **SM** and **DEPMAm** separately (Figures 3B and S19), affording the values presented in Table 2. The apparent rate constants for **SM** polymerization in the copolymers (≈0.010 min^–1^) are similar to those determined for the homopolymerization (≈0.012 min^–1^), while **DEPMAm** varies from almost zero to 0.004 min^–1^, indicating some dependency on feed ratio. Indeed, a plot of each monomer’s conversion over time demonstrates high (≥80%) final conversion for **SM** regardless of the initial concentration (Figure S19A), while **DEPMAm** conversion decreases as the apparent rate constant (and initial concentration) decreases. The general decrease in reaction rate with increasing **DEPMAm** feed ratio remains consistent with the lower reactivity of methacrylamides compared to methacrylates and has been observed in other methacrylamide/methacrylate copolymerization systems.^55,58^

**Table 2:** Apparent First Order Rate Constants and Goodness of Fit from Both Global and Individual Monomer Conversion

	SM + DEPMAm		SM			DEPMAm			
entry	*k*_app_ (10^–3^ min^–1^)	*r*^2^	*k*_app_ (10^–3^ min^–1^)	*r*^2^	conv. (%)	*k*_app_ (10^–3^ min^–1^)	*r*^2^	conv. (%)	*k*_rel_^a^
**S100**	12.1 ± 0.5	0.99	12.1 ± 0.5	0.99	91				3.6^e^
**S75**	5.0 ± 0.1	0.99	8.2 ± 0.1	0.99	80	0.4 ± 0.4^b^	0.02	14	21.2^b^
**S50**	4.8 ± 0.7	0.88	10.5 ± 0.8	0.96	86	2.2 ± 0.6	0.65	32	4.9
**S25**	5.0 ± 0.5	0.95	10.7 ± 1.2	0.93	89	4.1 ± 0.3	0.97	57	2.6
**S00**	3.3 ± 0.3	0.96				3.3 ± 0.3	0.96	49	

a Relative apparent rate constant of **SM** to **DEPMAm** given by *k*_app_SM/*k*_app_DEPMAm. In the case of homopolymerization, *k*_rel_S100 is given by *k*_app_S100/ *k*_app_S00.

b The value obtained for the relative apparent rates for the **S75** copolymerization contains large uncertainty, given the large relative error in the apparent rate of **DEPMAm** polymerization (Figure S20).

Since we noticed differences in reactivity rates of the monomers, we were interested in gaining further insight into the composition of our copolymers – notably with regard to the distribution of the monomers throughout the copolymer chains. Analyzing the reactivity ratios of monomers in a copolymerization enables a description of the deviation from random copolymerization and, consequently, the distribution of monomers on a growing polymer chain. To obtain reactivity ratios for **SM** and **DEPMAm**, data acquired from NMR measurements were analyzed using the Meyer-Lowry model (eq 1,^59^ and Figure 3C) as discussed by Lynd et al.^48^ In these equations, **SM** was denoted A and **DEPMAm** B for simplicity. Consequently, *f*_A_ is the mole fraction of **SM** (at time *t*), *f*_A_^0^ is the initial mole fraction of **SM**, and *r*_A_ and *r*_B_ are the reactivity ratios of **SM** and **DEPMAm,** respectively. [M]_*t*_ is the total monomer concentration at time *t,* and [M]_0_ is the total initial monomer concentration (eq S1). In the initial ([A]_0_ and [B]_0_) and time-dependent ([A]_*t*_ and [B]_*t*_) concentrations, A refers to **SM** while B refers to **DEPMAm**. For a more detailed discussion of this analysis, the reader is directed to Discussion S3.
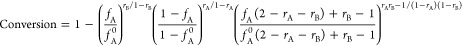
1where
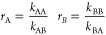


The reactivity ratios, *r*_A_ and *r*_B_, describe the propensity of **SM** and **DEPMAm** to self-propagate. Values >1 reveal a preference for self-propagation while values <1 indicate a preference for reacting with the comonomer. Global fitting was employed to minimize errors associated with individual runs (Figures 3C and S21). We obtained *r*_A_ and *r*_B_ values of 13.38 ± 0.87 and 0.99 ± 0.00(01), respectively, indicating that the copolymerization followed the behavior of a blocky gradient (*r*_A_ × *r*_B_ > 1; *r*_A_ > *r*_B_).^48^ Finding *r*_A_ > *r*_B_ remains consistent with the lower reactivity of methacrylamides compared to methacrylates.^55,58^

The experimentally determined reactivity ratios were used to calculate instantaneous (*F*_A_) copolymer compositions, which offer insight into the chain microstructure (Discussion S3, Figure S19B,C, eqs S1 and S2). We then plotted the average (<*F*_A_>) copolymer chain composition (Figure 3D, dotted lines) using eq 2, which is of particular use in targeting an <*F*_A_> for an initial feed ratio and conversion. Using the in situ monomer conversions in the copolymerizations as obtained by NMR, the calculated molar fraction of incorporated aldehyde units was 66, 27, and 6% for the **S25**, **S50** and **S75**, respectively. Comparing these values to those obtained for the purified copolymers (Figure 3D, filled circles), the **S25** and **S50** aldehyde contents are in good agreement with results determined by ^1^H NMR, while **S75** underestimates the obtained *F*_Ald_ value (6 vs 12%). Consequently, the Meyer-Lowry model appears promising for predicting a copolymer chain composition within an intermediate range (≈20–80%) at moderate (≈60%) monomer conversion given an initial molar feed ratio and final monomer conversion. However, larger studies are needed to validate this predictive power across the full range of compositions and monomer conversions.
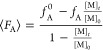
2

### 2.3 Hydrogel with Tunable Stiffness and Strain-Stiffening from **S25–S75** Copolymers via Dynamic Covalent Hydrazone Cross-Linking

We next explored if we could form hydrogels with these new copolymers via dynamic covalent imine-type cross-linking using adipic dihydrazide and propylbishydroxylamine (data not shown). However, these small molecule cross-linkers were unable to form hydrogels at low wt % (<6 wt %). We confirmed using *O*-ethylhydroxylamine that the hydroxylamines react with the aldehydes on our **S75** copolymer and believe that the small molecules may be unable to form hydrogels due to a predominance of intramolecular cross-linking (Figure S22). Consequently, we switched to a macromeric PEG dihydrazide cross-linker for hydrogelation studies. We screened different compositions of **S75** – possessing the lowest aldehyde content – and **PEG-HZ** (*M*_w_ ≈ 5 kg mol^–1^) via the vial inversion test in phosphate-buffered saline (PBS) at pH = 7.4 (Table S3). All formulations gelled rapidly (<10 s), slowing (<60 s) only when decreased to 2 wt % **S75**.

**Figure 4:**
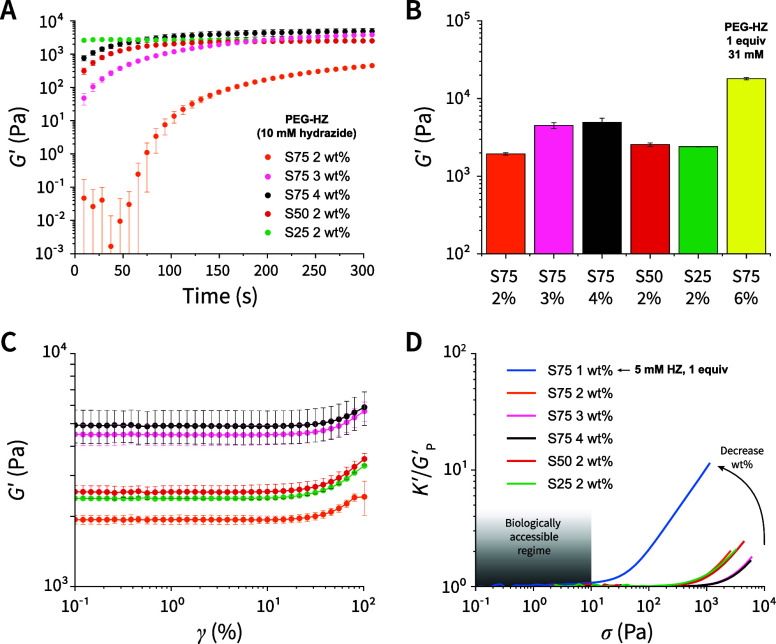
Effect of polymer content and composition on hydrogel stiffness for a fixed cross-linker concentration. (A) Time sweeps demonstrating the rapid cross-linking of **pSM-***co***-OMAm** hydrogels as a function of copolymer wt % and copolymer composition, while maintaining a constant concentration of cross-linker (**PEG-HZ**). (B) Shear storage moduli for the different **pSM-***co***-OMAm** copolymers at a fixed cross-linker concentration (Figure S22) reported as the mean ± standard deviation of 2–3 replicates. A 6 wt % **S75** hydrogel with 1 equiv (with respect to aldehydes) of **PEG-HZ** was included to indicate the potential range of accessible moduli. (C) Strain sweeps of the initial formulations varying either copolymer content or composition demonstrated strain-stiffening behavior. Complete strain sweeps can be found in Figure S23. (D) Normalized differential modulus (*K*′ = ∂σ/∂γ) versus stress (σ) of copolymer hydrogel formulations uncovered the tunability of the strain-stiffening regime. Reducing the copolymer concentration to 1 wt % and maintaining equimolar hydrazide dramatically decreases the critical stress (onset of strain-stiffening, σ_c_) while increasing the stiffening index (final slope, *m*). Both σ_c_, and *m* display a linear correlation with total wt %; see Figures S25, S27 and Table S5.

Encouraged by the above results, we wanted to assess the rheological properties of these hydrogels to understand how differences in both our chain topology and concentration affect hydrogel gelation and mechanical properties. We realized that attempting to maintain an equimolar relationship between the hydrazide and the aldehyde would lead to a large difference in total mass content (wt % of all polymeric species) between samples (**PEG-HZ** becomes a significant contributor). To minimize this, we decided to keep the total concentration of cross-linker constant (2.70 wt % **PEG-HZ**, [hydrazides] = 10 mM, 1 equiv hydrazide with respect to **S75** aldehyde concentration at 2 wt %), while varying either the copolymer wt % or the copolymer composition (Table S4). We observed that increasing the **S75** polymer concentration from 2 to 3 wt % decreases the gelation onset time from ≈60 to <8 s (Figure 4A and Table S4). A further increase to 4 wt % only slightly increased cross-linking speed, approaching the limits of sample preparation. Maintaining a constant 2 wt % copolymer concentration, and constant cross-linker concentration, while changing the copolymer composition (and thus increasing the aldehyde concentration) had a dramatic impact on cross-linking kinetics. The **S75** took ≈60 s to begin gelling while both the **S50** and **S25** had already begun gelling before acquisition began (<8 s). Surprisingly, **S25** had already reached a plateau storage modulus prior to the acquisition, showing no further increase in *G*′. We attributed this increase in cross-linking kinetics to a higher local concentration of aldehyde, enabling faster formation of a contiguous network. Full-time sweeps over the 30 min acquisition period can be found in Figure S23A. Frequency sweeps revealed that all formulations demonstrated frequency-independent moduli over the measured range (1–100 rad s^–1^) (Figure S23B). This was as expected as typical crossover frequencies for side-chain functionalized dynamic hydrazone cross-links are found at ≈10^–3^ rad s^–1^.^23,60^

Looking next at the final shear moduli obtained for our two series (Figure 4B), an increase in **S75** concentration from 2 to 4 wt % increased *G*′ from 1.9 to 4.9 kPa. However, keeping the copolymer (and cross-linker) concentration constant while changing the copolymer composition had a much smaller impact, with **S50** and **S25** at 2 wt % reaching 2.6 and 2.4 kPa, respectively; though the **S25** should be considered with care due to its extremely fast cross-linking. This finding suggests that the hydrogel properties are largely dictated by cross-linker concentration with only small deviations based on copolymer composition. To illustrate the potential range of hydrogel stiffnesses accessible with our copolymer systems, we also prepared a 6 wt % **S75** hydrogel with 1 equiv (hydrazide with respect to aldehyde) **PEG-HZ**, resulting in a *G*′ of 18 kPa–an order of magnitude increase in stiffness compared to the 2 wt % formulations (Figure S23C,D). As hydrogel systems are increasingly the target of models relating rate and equilibrium constants to macroscopic mechanical properties, we were curious if a recent phantom network model could successfully fit our hydrogel stiffnesses here based on reported equilibrium constants.^61,62^ Unfortunately, this model was unable to capture the behavior of our nonideal network and highlights the need for the development of advanced dynamic network models in the future.

Interestingly, while studying the rheological responses of our hydrogels, we noticed strain-stiffening behavior (Figure 4C and S24–S27). Strain-stiffening is common in natural polymers and a fundamental property of the native ECM, playing a key role in mechanotransduction and consequently cell fate.^63^ However, this phenomenon is rare in purely synthetic hydrogel systems.^64^ Therefore, we further evaluated the strain-stiffening properties by determining the differential modulus as a function of stress for the same data (Figure 4D). Our initial formulations possess critical stresses (σ_c_; onset of strain-stiffening; a measure of the sensitivity of the material to external force) ranging from 580 Pa (2 wt %) to 1740 Pa (4 wt %), increasing with the total mass content (and resulting stiffness) (Table S5). The stiffening parameter (*m*; a measure of the magnitude of the stiffening response) decreases slightly with increasing mass content from 2 to 4 wt % (Δ*m* = 0.08). In contrast to the mass content, changing the copolymer composition at a constant 2 wt % had only a minor impact on σ_c_ (**S75**:580 Pa vs **S25**:860 Pa) and a similar magnitude of impact on *m* (Δ*m* = 0.12). These results suggest that the network density is the dominant factor for strain-stiffening in our system.

To further explore this hypothesis, we studied a 1 wt % **S75** formulation (1 equiv hydrazide, 5 mM **PEG-HZ**, 2.35 total wt %) and saw a dramatic reduction in σ_c_ to 40 Pa, with a concomitant increase in *m* to 0.70 (cross-linking kinetics and frequency sweep of the 1% hydrogel can be found in Figure S23C,D). This result supports the importance of the network density for strain-stiffening behavior in our hydrogels. Further evidence is shown by pooling the data and plotting both σ_c_ and *m* against total mass content, which yields a general trend of decreasing σ_c_ (and increasing *m*) with decreasing mass content (Figure S27). Notably, a σ_c_ of 40 Pa is close to the biologically accessible range of σ_c_ (stresses that cells are able to exert on their surroundings)^65^ of ≈0.1–10 Pa.^66−68^ We expect that future optimization of total mass content and cross-linker equivalents could grant access to the biological regime in this copolymer system.

Only a few synthetic strain-stiffening systems have been designed,^64^ based on self-assembly of polyisocyanopeptides,^50,69,70^ bisurea bolaamphilphiles,^51,71^ block copolymers,^72^ or different phase domains.^73^ These systems mimicked the native strain-stiffening mechanism arising from the entanglement and bundling of fibrillar structures under strain. Consequently, they typically exhibited an increase in both the stiffening index and critical stress with increasing polymer (fiber) content.^64^ Recently, strain-stiffening hydrogels from synthetic, flexible polymers using DCvC for network junctions were reported using dynamic covalent imine formation,^74^ and boronate ester cross-linking.^49^ However, the strain-stiffening behavior reported in these systems did not follow the expected trend; instead of exhibiting an increase in the stiffening index at higher polymer concentrations, a decrease was observed. We found the same trend in our hydrazone-based copolymer hydrogels (Figures 4D, S25 and S27). The mechanism of strain-stiffening in flexible polymer hydrogels has been attributed, in part, to the entropic penalty (loss of conformational freedom) upon strain-induced chain extension.^75,76^ Webber et al. recently studied this unexpected strain-stiffening behavior in boronate ester hydrogels, and they propose a hybrid strain-stiffening mechanism arising from a combination of both entropic and enthalpic (bond deformation) contributions.^49^ Our results align with their proposed framework for strain-stiffening in flexible dynamic networks, though more in-depth studies will be needed to confirm whether dynamic covalent junctions alone are sufficient to induce the observed behavior. Curious, we also tested whether the strain-stiffening behavior was reproducible when subjected to cyclic strains of increasing amplitude (up to 300%) and found no major changes in the strain-loading behavior prior to rupture. After rupture, however, the plateau modulus could be recovered but not the yield strain (Figure S26). To understand more about the relationship between flexible dynamic covalent networks and strain-stiffening behavior, hydrogel systems including those reported herein will be a necessary tool while also holding promise for mimicking the strain-stiffening behavior present in native ECM for biomedical applications.

### 2.4 Microfluidic Printing of **S25** and **S75** Copolymer Hydrogels

To take advantage of our very rapidly cross-linking hydrogels (<8 s), we also performed a proof-of-concept experiment to print both **S25** and **S75** hydrogels using an Aspect microfluidic bioprinter. For this technique, polymer and cross-linker solutions meet just before entering the printing nozzle. The mixture resides only ≈5 s in the nozzle and must cross-link during this short window in order to extrude a fiber, making our rapid gelation times particularly attractive for this fabrication method. Currently, most work using this microfluidic system is performed using calcium cross-linker-based bioinks,^77−80^ so we wanted to explore whether our synthetic polymer system could expand the polymer library for this developing technology.

To this end, we used copolymer solutions of either **S75** (8.0 wt %, [aldehyde] = 40 mM) or **S25** (9.5 wt %, [aldehyde] = 280 mM) with a **PEG-HZ** (13 wt %, [hydrazide] = 50 mM) cross-linking solution. Despite these relatively high polymer concentrations, the viscosity of these stock solutions was sufficiently low to flow freely through the microfluidic channels of the print head under pressure. The interfacial gelation kinetics were suited to extrude rigid fibers (Figure S28A). Moreover, we successfully fabricated two simple geometries (Figure S28B,C). Given the relatively large difference in aldehyde concentration between **S75** and **S25**, these results indicated that the presented copolymer platform could potentially be used as a tunable (bio)ink for this printing technology and offers an avenue for future research.

### 2.5 Sequential and One-Pot Functionalization of pSM-*co*-OMAm via Oxime Ligation

**Figure 5:**
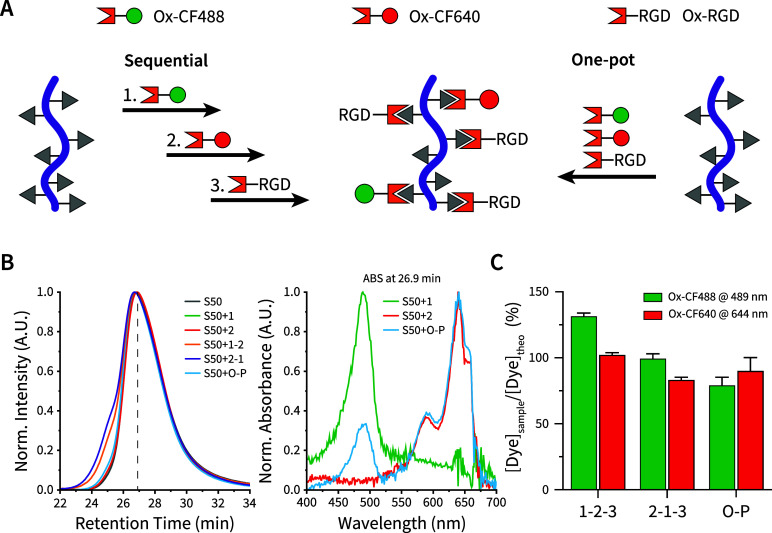
Modularity of the synthetic platform for (bio)molecule conjugation via either sequential or one-pot reactions. (A) Aminooxy-conjugated dyes (**Ox-CF488, 1** and **Ox-CF640, 2**), as well as aminooxy-RGD (**Ox-RGD, 3**), were attached onto **S50** via oxime ligation using either a sequential or one-pot (**O–P**) reaction route. In the sequential route, two reaction sequences were investigated: **1-2-3** and **2-1-3**. After each reaction, a purification step via dialysis was performed. (B, left) Retention times of the products (**S50**, **S50+1**, **S50+2**, **S50+1-2**, **S50+2-1**, and **S50+O–P**) were evaluated by GPC. (B, right) To confirm coupling of the dye to the polymer, we assessed the absorption spectra at an elution time of 26.9 min for **S50+1**, **S50+2**, and **S50+O–P**, showing the characteristic absorption spectra of both **Ox-CF488** and **Ox-CF640.** (C) Ratio of measured to targeted dye concentrations in the purified samples (**S50+1-2-3**, **S50+2-1-3**, and **S50+O–P**) was determined via UV–vis at 489 (**Ox-CF488**) and 644 nm (**Ox-CF640**). Values reported are the mean ± standard deviation (*N* = 2, and *n* = 5).

Biofunctionalization of polymer platforms is a critical step in the creation of effective biomaterials. However, different tissues and applications can require different bioactives, or cocktails of bioactives, for optimal function. For example, recent work highlighted the importance of synergistic presentation of bioactive molecules and their agonistic effect on cell fate.^81−84^ Modularity, where the biomaterial can have the desired bioactives attached via mixing at the lab bench, is an attractive characteristic of a platform that can be tailored for several applications. The presented copolymer with pendant aldehydes provides opportunities to add ligands via bio-orthogonal conjugation, in particular via oxime ligation,^85^ in addition to utilizing the dynamic covalent hydrazone cross-linking (vide supra). We envisioned that the copolymer could be used for decoration with aminooxy-functionalized ligands according to a mix-and-match principle. To this end, we validated both a sequential and a one-pot synthetic route to attach a cocktail of aminooxy-functionalized molecules onto the **S50** polymer (Figure 5A). We chose two commercially available aminooxy-functionalized fluorophores, CF488A (**Ox-CF488**, **1**) and CF640R (**Ox-CF640**, **2**), and an aminooxy-functionalized RGD peptide (**Ox-RGD**, **3**) to demonstrate this principle.

We began by reacting a 4.4 × 10^–2^ μmol (1.7 × 10^–3^ equiv with respect to aldehyde groups) solution of either **1** or **2** with a solution of **S50**. Following purification, each product (**S50+1**, **S50+2**) was reacted again with either **2** or **1,** respectively, testing both **1**–**2** and **2**–**1** reaction sequences. Both reaction sequences (**S50+1-2**, **S50+2-1**) were then reacted with **3** (0.75 μmol, 5.0 × 10^–2^ equiv with respect to aldehyde groups). In addition, we performed a one-pot synthesis in which 3.9 × 10^–2^ μmol of both **1** and **2** (1.7 × 10^–3^ equiv with respect to aldehyde groups), and 3.9 × 10^–1^ μmol of **3** (1.7 × 10^–2^ equiv with respect to aldehyde groups) were coupled simultaneously (**S50+O–P**). One reaction (**S50+1/S50+2/S50+O–P**) did not lead to any apparent changes in the dispersity of the polymer, while two sequential reactions (**S50+1-2** and **S50+2-1**) resulted in a small broadening compared to pristine **S50** (*Đ* ≈ 1.26 vs *Đ* ≈ 1.18; Figure 5B, **left**). Evaluation of their absorption spectra at maximum elution signals visualized the simultaneous elution of dye and polymer, confirming their attachment to each other (Figures 5B, right, and S29).

After finalizing the sequential reaction routes by addition of **Ox-RGD**, we used ^1^H NMR spectroscopy to verify successful coupling, as indicated by the appearance of characteristic RGD and *Z*/*E* oxime peaks at 4.4, 7.0, and 7.7 ppm, respectively (Figure S30). We then estimated the percentage of dye attachment using UV–vis measurements (Figures 5C and S31). Overall, the results indicated a high degree of ligation with only small differences between either sequential (**1–2–3** and **2–1–3**) or one-pot (**O–P**) synthesis routes.

We wondered whether this biofunctionalization affected gelation. In order to test this, we transformed **S50+O–P** (2% functionalization) into a 1.5 wt % hydrogel ([aldehyde] = 18.8 mM) with 1 equiv of hydrazide cross-linker. We observed that gelation still took place and appeared to be slower (≈50 s) than previous samples (Figure S32A). Thus, we sought to explore what degree of decoration was possible before the mechanical properties of the copolymer were affected or a hydrogel was no longer able to be formed (Figure S32B). Next, we prefunctionalized **S50** with 0.2, 0.6, or 0.9 equiv of a small molecule oxime (*O*-ethylhydroxylamine) prior to forming 2 wt % **S50** hydrogels with 0.4, 0.4, or 0.1 equiv **PEG-HZ**, respectively. We found that 20% prefunctionalization delayed the onset of cross-linking (from <6 to ≈90 s) and rate of cross-linking without impacting the final shear storage modulus. If more functionalization was added (60%) then gelation significantly slowed (≈9 min) and the final network modulus was reduced by over a decade. Prefunctionalization of 90% of available aldehydes prevented gelation within the 20 min measurement.

Taken together, these results indicate that this copolymer system is a modular platform that enables facile decoration with multiple ligands depending on the desired biological application while maintaining its ability to form a hydrogel via rapid hydrazide cross-linking (at ≤20% prefunctionalization). Although a one-pot reaction requires less work to obtain a multifunctionalized product, the sequential pathway shows that the addition of another biomolecule is possible at a later stage. Additionally, the ability to modulate the onset of hydrogelation by controlling the fraction of aldehydes available for cross-linking enables applications that require delayed network formation. For example, it is for maintaining handleability and pliability during surgical manipulation and minimally invasive procedures.

### 2.6 FRET Study Demonstrates the Release of Bound Ligands via Dynamic Competitive Displacement

The spatiotemporal presentation of bioactive cues regulates numerous processes in the native ECM, including cellular differentiation, morphogenesis, maturation, and disease progression, and remains a challenge in synthetically designed ECMs.^86^ However, commonly employed covalent bonds fail to capture the spatiotemporal presentation of ligands inherent to the native ECM. Inspired by Boekhoven et al., who showed that weakly bound ligands could be replaced by strong binding ones using host–guest chemistry,^20^ we aimed to leverage the difference in reaction equilibrium constants (i.e., reversibility) of the imine-type bonds to replace one ligand with another on our copolymer system. Whether this replacement proceeds via an associative or dissociative mechanism (transimination vs hydrolysis) under aqueous conditions remains unclear and presents an interesting target for future studies.^87^

**Figure 6:**
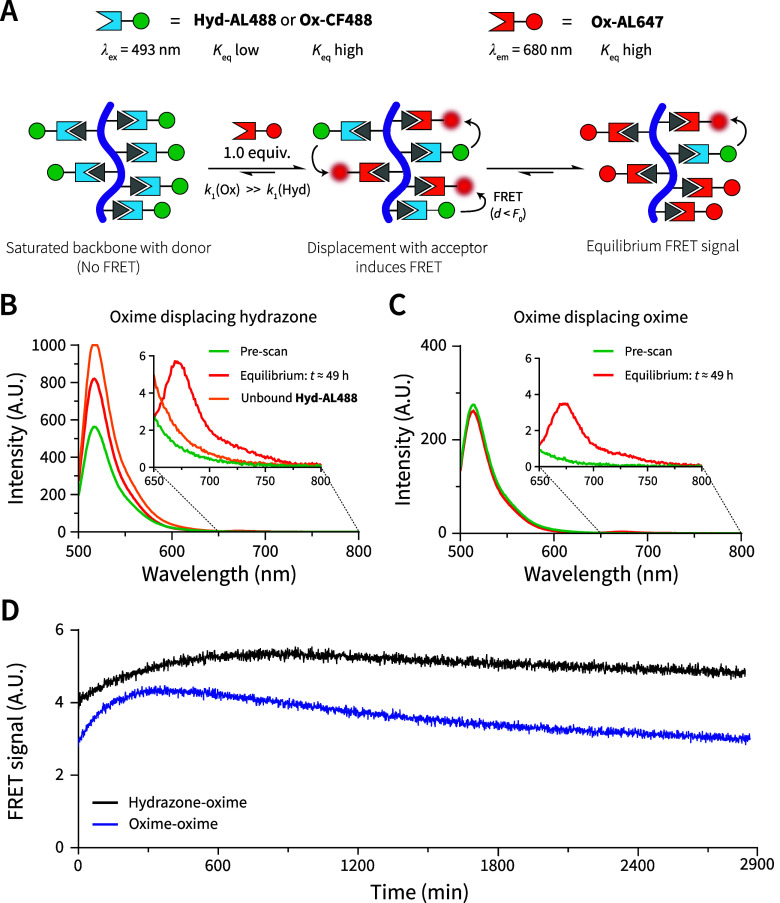
FRET measurements demonstrate competitive displacement of ligands on the polymer system. (A) Schematic representation of experimental setup. The pendant aldehydes present in **S25** were first mixed with 1.0 equiv of a hydrazide (**Hyd-AL488**, *K*_eq_ low) or aminooxy (**Ox-CF488**; *K*_eq_ high) conjugated donor dye. Upon addition of 1.0 equiv of **Ox-AL647** (acceptor), we would expect the evolution of a FRET signal (λ_em_ = 680 nm upon excitation at 493 nm). (B + C) Emission spectra from 500 to 800 nm (λ_ex_ = 493 nm) were recorded before addition of the **Ox-AL647** (prescan, green) and after displacement (*t* ≈ 49 h, red), illustrating the absence and subsequent presence of a FRET signal (*n* = 3). In addition, **Hyd-AL488** was measured at the same concentration as the FRET study without copolymer present (unbound **Hyd-AL488**, orange in (B)). (D) After addition of the acceptor, the evolution of the FRET signal was tracked over ≈49 h for both **S25** mixed with either **Hyd-AL488** (black, *n* = 1) or **Ox-CF488** (blue, *n* = 3). All raw data of the fluorescent intensities of dyes during the measurement can be found in Figure S33A,B. The **Hyd-AL488** displayed a shift in λ_max_ and intensity in the bound versus unbound state. Due to the evolving spectrum, we decided not to report the FRET ratio, which can be found in Figure S33C.

To probe the exchange dynamics of competing ligands, we chose to use fluorescence resonance energy transfer (FRET). FRET is a powerful tool for studying the proximity of (partner) molecules on length scales of several nanometers based on the Förster radius (*F*_0_, the distance between FRET pairs where FRET efficiency is 50%).^88^ For example, the Meijer group has used FRET to showcase the speed of exchange of benzene-1,3,5-tricarboxamide units from a self-assembled stack depending on (a)chirality.^89^ Here, we selected two commercially available FRET pairs: aminooxy-functionalized Alexa Fluor647 (**Ox-AL647**, *K*_eq, oxime_ ≈ 10^8^ L mol^–1^)^85^ as the FRET acceptor and either hydrazide-functionalized Alexa Fluor488 (**Hyd-AL488**, *K*_eq, hydrazone_ ≈ 10^4^ L mol^–1^)^85^ or aminooxy-functionalized **Ox-CF488** (*K*_eq, oxime_ ≈ 10^8^ L mol^–1^) dyes as donors (Figure 6A). The *F*_0_ for these FRET pairs is ≈57 Å, according to the manufacturer’s reported formula,^90^ and we estimated the maximum distance between adjacent aldehydes in **S25** to be ≈22 Å (14 linear bonds – ignoring bond angles – of ≈1.54 Å).

We mixed **S25** with 1.0 equiv (with respect to aldehydes) of either **Hyd-AL488** or **Ox-CF488** and measured the emission spectra of these donors (λ_ex_ = 493 nm, λ_em_ = 515 nm). Then, we added a further 1.0 equiv of **Ox-AL647** (acceptor) and followed the evolution of the donor, acceptor, and FRET signals for ≈49 h. Finally, we recorded an end-point scan of the same emission spectra to highlight the presence of the newly appeared FRET signal (Figure 6B, C). In both cases, we observed the emergence of a FRET signal during the reaction. However, we noticed a drop in emission intensity and a red shift in λ_max_ of **Hyd-AL488** in the bound versus unbound state of the polymer (Figures 6B and S33). This observation prevents us from using the FRET ratio to observe oxime replacing hydrazone; instead, we examined the evolution of the raw FRET signal over time (Figure 4D).

After the addition of **Ox-AL647**, we observed the signal starting at a nonzero value in both mixtures. We attributed this initial FRET signal to the fast reaction of the added competing dye to unoccupied aldehydes, which aligned with the rapid reaction observed during hydrogelation (Figure 3A). To test whether our prefunctionalized **S25** was saturated, we mixed **S25** in a separate experiment with either 1 or 2 equiv of **Hyd-AL488** or **Ox-CF488**. After purification, the absorbance value of 2 equiv was higher than 1 equiv, indicating that free sites indeed were present (Figure S34). Continuing to analyze the FRET signal, we observed a further increase in both mixtures until a maximum is reached. This gradual increase (over hours) after the initially observed nonzero values supports the replacement of the acceptor by the donor. The oxime–oxime FRET increase proceeds faster than the hydrazone-oxime displacement, as evidenced by a steeper slope and earlier signal maximum (≈5 vs ≈10 h), which could suggest faster conjugation/displacement in the oxime–oxime system (Figure 4D).

Notably, we observed a slight decrease in the FRET signal following the observed maximum (expected to arise at 50:50 occupancy), in both systems. We would expect the hydrazone-oxime system to tend toward a mostly oxime-functionalized polymer based on their differences in *K*_eq_, leading to a decrease in the FRET signal after the maximum. Following the same logic, we would expect more of a plateau in the oxime–oxime system. The origin of this deviation is not fully understood and remains to be elucidated with further detailed experimentation. The gradual drop of the signal might be explained in part by slight photobleaching of the dyes over multiday measurements (Figure S33). In addition, the dyes may have different reactivities; the exact chemical structure of the commercially available aminooxy-functionalized dyes is unknown.

Together, the obtained spectroscopic evidence shows that our copolymers can easily be decorated with aminooxy-terminated dyes. Furthermore, multiple aminooxy ligands can be attached both sequentially (to a prefunctionalized copolymer backbone) or simultaneously in an accessible one-pot method. In addition, the evolution of the FRET signal as well as the evolving **Hyd-AL488** signal indicated that bound dyes could be released upon the introduction of a competing oxime-forming nucleophile. As the maximum FRET signal was reached over the course of a day, the speed of ligand displacement along the copolymer falls within biologically relevant time scales. While here we explore ligand exchange in the context of small molecule release and multifunctionalization, recent studies by Heilshorn and colleagues and within our own lab have shown that competitive binding in dynamic systems can also be used to modulate macroscopic hydrogel properties.^62,91^ Specifically, the kinetic and thermodynamic parameters for each molecular reaction are connected to the resulting network topology. These studies highlight the range of binding and rate constants accessible to dynamic covalent reactions and the potential for tuning response times, release and binding rates, and time-dependent concentration profiles.

To translate the displacement of ligands from solutions toward a hydrogel system, we attempted to use FRET to demonstrate the displacement of ligands using the same dye combinations in 3D-cross-linked hydrogel droplets. However, the high concentration of dye required to saturate the aldehydes prevented light from passing through the hydrogel, led to quenching, and resulted in a low signal (Figure S35). Instead, we turned to fluorescent recovery after photobleaching (FRAP) experiments to assess the mobility of bound dyes after the addition of a competitive dye (Figure S36). We could observe the diffusion of the newly added dye into the bleached area. We also observed that the addition of amino-oxy dye to a bound hydrazone dye increased the FRAP recovery of the hydrazone dye, which suggests displacement. No FRAP recovery of a bound oxime dye was examined when adding hydrazide dye. Of note, the magnitude of the recovery signals was too small to draw firm conclusions, and further experiments are required to confirm the extent and rate of displacement at a molecular level.

Looking to show a large macroscopic change, we wanted to leverage the replacement of hydrazone by aminooxy to decross-link a freestanding hydrogel. We prepared hydrazone-cross-linked hydrogel droplets (**S25**, 1 wt %, 0.2 equiv **PEG-HZ** hydrazides with respect to aldehydes, trace **Ox-AL647** for visualization) and added ≈2 equiv (with respect to aldehydes) of a small molecule aminooxy competitor (Figure S37). We observed that within 24 h, the hydrogel was dissolved, whereas a control hydrogel incubated in only PBS remained intact. This responsive behavior has utility for recovering embedded cells/organoids, which can be important not only for clinical translation but also for cellular analysis techniques such as gene/protein expression, single-cell analyses, and cell sorting. These techniques can be challenging to perform in the presence of a hydrogel. Additionally, partial replacement enables spatiotemporal modulation of the hydrogel mechanical properties.

### 2.7 In Vitro Cytocompatibility and Release of HDFs on **S25–S75** Copolymer Hydrogels

**Figure 7:**
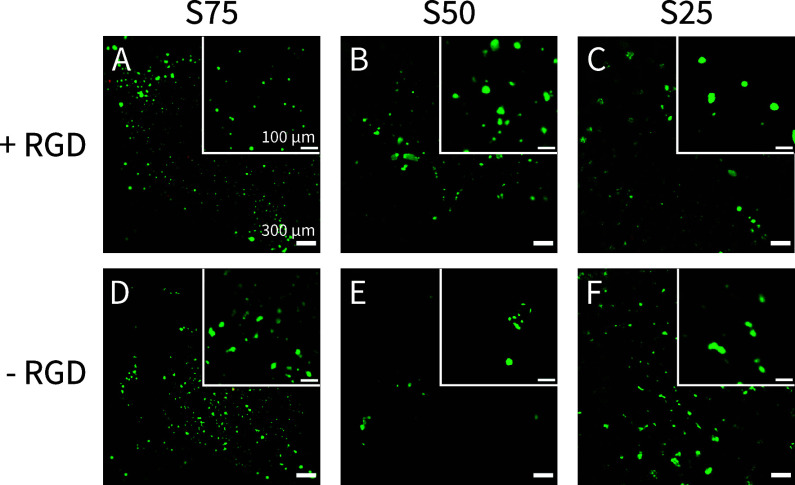
Live–dead staining of human dermal fibroblasts (HDFs) seeded on **S75–S25** hydrogels shows good cytocompatibility. HDFs were seeded on copolymer hydrogels (**S75**–**S25**) with (A–C) and without (D–F) 1.0 mM **Ox-RGD**. (A,D) **S75**, (B,E) **S50**, and (C,F) **S25**, display live (Calcein-AM, green) and dead (Ethidium homodimer-1, red) cells on top of the hydrogels after 20 h. Observing the absence of dead cells in these images, we also tested 2D samples as a positive control for the live dead staining (Figure S41). Scale bars are 300 and 100 μm for the overview and insets, respectively, *N* = 2.

To investigate the biocompatibility of this platform, we cultured human dermal fibroblasts (HDFs) on 2 wt % hydrogels of **S75**, **S50**, and **S25**, maintaining a constant cross-linker concentration of 10 mM – corresponding to the number of equivalents with respect to aldehyde groups of 1.0, 0.4, and 0.18 respectively. We also investigated whether the presence of 1.0 mM **Ox-RGD** would facilitate cell adhesion and spreading morphology. Staining (calcein-AM/ethidium homodimer-1 for live/dead cells) and fluorescent imaging after 20 h showed good cell viability with no apparent effect of the different chain composition or presence of **Ox-RGD** (Figure 7). Interestingly, while imaging our hydrogels, we observed diffraction patterns that unfortunately led to slightly blurry images and precluded accurate quantification of HDF viability (Figure S38). We suspect that the appearance of these patterns may be due to the fast gelation kinetics (Figure 3A), leading to heterogeneous distribution of the cross-linker throughout the gel, and consequently, the creation of different optical domains.

Since we did not observe cell spreading at an **Ox-RGD** concentration of 1.0 mM, we decided to increase the amount to 2.5–10 mM on 2 wt % **S50** hydrogels. Again, no cell spreading was observed for all RGD concentrations after 3 days of culture (Figure S39). During this culture period, LDH assays supported that HDFs remained more viable on top of hydrogels containing RGD than hydrogels without RGD (Figure S40). The prevention of anoikis in rounded cells on softer (≈1 kPa) substrates has been reported in the literature,^92^ and could be complicated by interactions between free aldehydes and cell membranes,^93^ as well as RGD surface density.^94^ Moreover, Anseth and co-workers very recently showed that cell spreading on synthetic hydrogels was only exhibited when the critical stress for strain-stiffening was within the biologically relevant stress regime.^95^ The effect of strain-stiffening on cell spreading in our system is a target for future biological studies.

Encouraged by the stimuli-responsive decross-linking and cell viability, we hypothesized that we could release cells on demand for harvesting or delivery. After culturing HDFs for 3 days on **S50** hydrogels containing no RGD or 2.5–10 mM of RGD, we added 1.0 equiv of *O*-ethylhydroxylamine as a competitor to the media. After 7–9 h, we observed that the hydrogels de-cross-linked, leading to a release of the HDFs to the underlying tissue culture plastic (TCP). Notably, without RGD present in the initial hydrogel, the HDFs did not survive and grow on the TCP; the HDFs seeded on hydrogels containing RGD grew well on the TCP after 3 days (Figure S40B, right). This supported the observation that the HDFs remained more viable in the presence of RGD even though cell spreading was absent.

Taken together, we demonstrated HDF viability in our copolymer hydrogel system with temporal control over release of cells into their surroundings. Given the nature of these hydrogels, we also envision applications in time-controlled drug release or the swapping of bioactivity using competitive molecules. Interestingly, while not explored in this work, sulfonate groups have been used for the controlled release of charged proteins.^96^ As such, we could explore in future research whether sulfonates facilitate a secondary uptake-release mechanism, which could further strengthen the biological application of this copolymer platform.

## 3 Conclusions

Here, we present the controlled RAFT synthesis of a well-defined, water-soluble copolymer with targetable aldehyde and sulfonate composition. Subsequent analysis of the reaction kinetics and determination of the reactivity ratios revealed that all copolymers had a blocky gradient microstructure. We then showed that this platform can be used to rapidly form hydrogels with tunable stiffness and strain-stiffening behavior and is compatible with microfluidic printing. We also demonstrated that the reversible nature of dynamic covalent hydrazone and oxime bonds can be harnessed for the ligation, as well as subsequent displacement, of ligands via competitive binding. Finally, we show HDF viability for biomaterial applications and notice potential for cell delivery applications by de-cross-linking hydrogels. This array of controllable mechanical properties, in conjunction with a well-defined chemical composition, cytocompatibility, and temporal control over ligand release, provides a novel synthetic platform to be exploited for diverse dynamic hydrogel applications and to foster the development of next-generation biomaterials.

## 4 Experimental Section

### 4.1 Materials

All chemicals were purchased from commercial suppliers and used as received, unless otherwise specified.

### 4.2 Synthesis of *N*-(3,3-Diethoxypropyl)-methacrylamide

We adopted a protocol reported by Wang et al. for the synthesis of **DEPMAm**.^52^ Glassware used for the reaction was oven-dried prior to use. In a dry N_2_ atmosphere, methacryloyl chloride (5.5 mL, 56 mmol, 1.1 equiv) was added dropwise to a precooled solution of 1-amino-3,3-diethoxypropane (8.2 mL, 51 mmol, 1.0 equiv) and triethylamine (7.8 mL, 56 mmol, 1.1 equiv) in 110 mL anhydrous dichloromethane (DCM) at 0 °C. The reaction was left to stir for 3 h; the ice-salt bath was not replaced during this time. The crude reaction mixture was washed with 100 mL of 1× 0.1 M HCl, 2× saturated NaHCO_3_, 2× dH_2_O, and 2× brine. After drying over anhydrous MgSO_4_, the DCM was removed under reduced pressure to yield a transparent yellow oil. This oil was passed through a silica plug (≈22 g of silica) using ≈300 mL of ethyl acetate as the mobile phase, followed by rotary evaporation to remove the solvent. This gave a pale yellow oil (9.33 g, 77.4% crude yield, 80% pure). ^1^H NMR (700 MHz, DMSO-*d*_6_) analysis revealed that two major amide impurities are present. The desired methacrylamide was isolated from the crude product by silica gel column chromatography (9:1 *v*/*v* DCM:acetone, TLC: *R*_f_ = 0.39; UV = 254 nm, KMnO_4_, ≈300 g silica, and ≈1000 mL mobile phase) and collected under reduced pressure as a very pale yellow oil. Of note, the impurities have a distinctive fruity odor which is conspicuously absent from the purified product, enabling a qualitative verification of separation by olfactory examination. Final yield = 4.87 g, 40%. ^1^H NMR (700 MHz, DMSO-*d*_6_, δ in ppm): δ 1.11 (t, 6H, *J* = 7.1 Hz, CH_2_C**H**_**3**_), 1.70 (dt, 2H, *J* = 5.6, 6.0 Hz, CHC**H**_**2**_CH_2_), 1.84 (t, 3H, *J* = 1.1 Hz, C = CC**H**_**3**_), 3.14 (dt, 2H, *J* = 6.0, 6.0 Hz, C**H**_**2**_NH), 3.43 (dq*, 2H, *J* = 7.1 Hz, CH_3_C**H**_**2**_O), 3.56 (dq*, 2H, *J* = 7.1 Hz, CH_3_C**H**_**2**_O), 4.51 (t, 1H, *J* = 5.6 Hz, C**H**OO), 5.31 (quint, 1H, *J* = 1.5 Hz, C = C**H**_**b**_), 5.62 (t, 1H, *J* = 1.1 Hz, C = C**H**_**a**_), 7.85 (t, 1H, *J* = 5.2 Hz, N**H**). ^13^C NMR (176 MHz, DMSO-*d*_6_, δ in ppm): δ 15.2 (CH_2_**C**H_3_), 18.5 (C = C**C**H_3_), 33.2 (CH**C**H_2_CH_2_), 35.0 (**C**H_2_NH), 60.6 (**C**H_2_O), 100.6 (**C**HOO), 118.7 (**C**=CCH_3_), 139.9 (C = **C**CH_3_), 167.2 (N**C**=O). ATR-FTIR (neat) cm^–1^: 3335 (w), 1656 (m), 1616 (m), 1527 (m), 1217 (w), 1128 (s), 1055 (s).

#### 4.2.1 Note

*Peaks at 3.43 and 3.56 ppm are denoted as double quartets, but these groups are two individual overlapping quartets. Magnetic inequivalence arising from the lack of a symmetric plane splits the two hydrogens into the ethyl ether groups (−OC**H**_**2**_CH_3_). As a result, a total of four different microenvironments are present, leading to four quartets. All quartets couple back to the terminal methyl group (*J* = 7.1 Hz).

### 4.3 Deprotection of DEPMAm

The details of this synthesis procedure (Scheme S1) and isolation attempts are described in the Supporting Methods.

### 4.4 RAFT (co)Polymerization of poly(3-Sulfopropyl Methacrylate), poly(*N*-(3-Oxopropyl) Methacrylamide), and poly(3-Sulfopropyl Methacrylate-*co*-*N*-(3-oxopropyl) Methacrylamide)

A series of RAFT (co)polymerizations were performed using CPPA as the chain transfer agent and 4,4′-Azobis(4-cyanopentanoic acid) (ACPA) as the initiator and with different feed ratios of **SM** to **DEPMAm**. Feed ratios are denoted as **S00–S100**, corresponding to the desired mole percent of **SM** monomer and are listed in Table 3 below where *f*_SM_ corresponds to the mole fraction of **SM**, and equiv is the number of equivalents with respect to CPPA. The total monomer concentration, CPPA concentration, and ACPA concentration were kept constant at 1.0 M, 4.0 mM, and 1.1 mM, respectively, in all (co)polymerizations.

**Table 3:** Reaction Compositions and Yield for Each Monomer Feed Ratio Studied in This Work

	SM				DEPMAm				
entry	*f*_SM_	equiv	moles (mmol)	mass (mg)	*f*_DEPMAm_	equiv	moles (mmol)	mass (mg)	yield (mg, [%])
**S00**	0.00	0.0	0.00	0	1.00	250.0	4.06	874	200 [24]
**S25**	0.25	62.5	1.01	250	0.75	187.5	3.04	655	245 [38]
**S50**	0.50	125.0	2.03	500	0.50	125.0	2.03	437	410 [44]
**S75**	0.75	187.5	3.04	750	0.25	62.5	1.01	218	490 [51]
**S100**	1.00	250.0	4.06	1000	0.00	0.0	0.00	0	600 [60]

For each reaction, SM was first dissolved in 1.8 mL of 1:1 *v*/*v* dH_2_O:1,4-dioxane. After the new volume was determined with a micropipette to account for differences in monomer solution volume, the SM solution and the **DEPMAm** were added to a round-bottomed flask. Next, 4.54 mg of CPPA (16.2 μmol, 1 equiv) and 1.23 mg of ACPA (4.4 μmol, 0.27 equiv) were added from stock solutions (1:1 *v*/*v* dH_2_O:1,4-dioxane). The final volume was then adjusted to 4.06 mL with 1:1 *v*/*v* dH_2_O:1,4-dioxane. After dry N_2_ gas was bubbled through the solution for 45 min at RT, the flask was submerged in an oil bath at 70 °C for 200 min while maintaining positive dry N_2_ pressure. Aliquots (≈35 μL) were collected under dry conditions with N_2_-flushed syringes at *t* = 20, 40, 60, 90, 120, and 200 min. The aliquots of the crude reaction mixtures were rapidly cooled in air and immediately diluted in 700 μL of DMSO-*d*_6_ for NMR and GPC analysis; an exception here was the **S100** reaction, which was insoluble in DMSO-*d*_6_ so phosphate buffered D_2_O (vide infra) was used. After 200 min, the remaining reaction mixture was transferred to a 3.5 kDa MWCO Snakeskin dialysis membrane and dialyzed sequentially against 0.1 M HCl, 50 mM NaCl (periodically neutralized with saturated NaHCO_3_), 25 mM NaCl, and finally distilled water (dH_2_O). The resulting (co)polymers were collected as pale pink to white fluffy solids after lyophilization.

### 4.5 Small-Scale, Neutralized RAFT Copolymerization of Equimolar SM and DEPMAm

Details of a RAFT polymerization of equimolar **SM** and **DEPMAm** (Scheme S2) in a neutralized reaction mixture are described in the Supporting Methods.

### 4.6 Free Radical Copolymerization of Equimolar SM and DEPMAm

Details of the free radical polymerization of equimolar **SM** and **DEPMAm** are described in the Supporting Methods.

### 4.7 Preparation of Phosphate Buffered D_2_O for NMR Analyses

To a fresh 100 g bottle of D_2_O, we added 381 mg of K_3_PO_4_, 463 mg of KD_2_PO_4_, and 100 μL of 100 mM DSS-*d*_6_ to yield a final phosphate concentration of 56.9 mM and DSS-*d*_6_ concentration of 0.11 mM. The pH was measured to be 7.42. Unless otherwise specified, all NMR spectra measured in D_2_O were measured using this phosphate-buffered D_2_O.

### 4.8 NMR Spectroscopy

All NMR spectra were recorded at 299.7 K using a Bruker Avance III HD 700 MHz spectrometer equipped with a cryogenically cooled three-channel TCI probe and analyzed with the TopSpin 4.0 software (Bruker, Germany). Standard phase correction, baseline correction, and referencing commands were used to process the fid file, except for the spectra obtained from crude mixtures taken during (co)polymerization. To achieve a more accurate baseline correction in these spectra, we set spline files across the polymer series using the *.baslspts* command.

### 4.9 NMR Sample Preparation of Purified Polymers

NMR spectra of purified **S25–S100** were obtained in D_2_O. To obtain spectra of the purified **S25–S100** in DMSO-*d*_*6*_, samples (≈5.0 mg) were predissolved in 70 μL of phosphate buffered D_2_O (pH = 6, adjusted with 0.5 M HCl) and further diluted with 630 μL DMSO-*d*_*6*_. **S50+1-2-3**, **S50+2-1-3**, and **S50+O–P** were predissolved in 95 μL of phosphate buffered D_2_O (pH = 6, adjusted with 0.5 M HCl), and further diluted in 505 μL of phosphate buffered D_2_O.

### 4.10 NMR Analysis of (Co)polymerization Reactions at Different Feed Ratios

The feed ratio of the copolymers was determined via ^1^H NMR (700 MHz, DMSO-*d*_6_) at *t* = 0 min via the integral ratio of the vinylic protons of the methacrylate (1H, 6.00–6.05 ppm) to the methacrylate and methacrylamide (1H, 5.64–5.59 ppm) monomer.

Analysis of monomer conversion of RAFT (co)polymerization was determined via the ^1^H NMR (700 MHz, DMSO-*d*_6_) spectrum of the crude reaction mixture. The integral of the 1H methacrylate peak (6.00–6.05 ppm, *I*_Ma_) was set to 1.00 except for **S00** entry, in which we set the 1H of the methacrylamide peak (5.64–5.59 ppm, *I*_MAm_) to 1.00. Subsequently, the conversion was calculated from the integral ratio of the polymer backbone peak (3H, 0.45–1.02 ppm, *I*_bb_) to the sum of the backbone peak and both vinylic monomer signals:
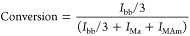
3

^1^H NMR spectra (700 MHz, D_2_O) of purified products were used to determine the fraction of aldehyde incorporation as well as the *M*_n._ The incorporated fraction of backbone units containing free aldehyde was determined from the integral ratio of the free and hydrated aldehyde (5.05–5.18 and 9.67–9.77 ppm, respectively) to the 2H of the CH_2_ moiety adjacent to the sulfonate group (3.9–4.4 ppm), according to 2(A + A*)/(2(A + A*) + a), see Figure 2. The *M*_n_ was determined from the integral ratio of the 3H of the methyl group of the backbone, and the aromatic 5H of the chain transfer agent was multiplied by the average molecular weight of the monomeric unit according to
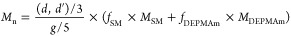
4

### 4.11 GPC Analysis of (Co)polymerization Reactions

Molecular weight, dispersity, and UV–vis absorbance of the (co)polymers were determined by GPC on a Prominence-I LC-2030C3D LC (Shimadzu) system comprised of an autosampler and a Shodex SB-G 6B guard (6.0 × 50 mm) column, connected to a dual setup of Shodex SB-803/SB804 HQ (8.0 × 300 mm) columns in series. These were followed by a refractive index detector and a photodiode array detector. The mobile phase used was 0.1 M NaNO_3_ in dH_2_O at a flow rate of 1 mL min^–1^ at 25 °C, using PEG standards up to 545,000 *M*_W_ (PEG calibration kit, Agilent Technologies). Purified samples were always dissolved at a concentration of 1.0 mg mL^–1^ in 0.1 M NaNO_3_. Regarding the crude samples for the kinetics study, 80 μL of the samples in DMSO-*d*_6_, (see above) was further diluted in 720 μL of 0.1 M NaNO_3_. All samples were filtered through 0.2 μm pores prior to running at an injection volume of 50 μL.

### 4.12 ATR-FTIR Analysis of Monomers and (Co)polymerization Products

ATR-FTIR spectra were recorded on a Nicolet iS50 FT-IR instrument (ThermoFisher) in the range of 4000–400 cm^–1^. Prior to 32 scans for sample measurement, 16 scans were recorded as the background. Spectra were baseline corrected in MATLAB (R2020b) using the “*msbackadj*” function with the “*pchip*” regression method, “*lowess*” smoothing method, and a window size of 500.

### 4.13 DSC Analysis of (Co)polymerization Products

The thermal properties of **S25–S100** polymers were determined by differential scanning calorimetry using a TA Instruments DSC250. Due to the insolubility of **S00**, we excluded this sample from DSC evaluation. Samples (±5 mg) were heated under a nitrogen atmosphere in *T*_zero_ aluminum pans with hermetic lids from RT to 200 °C at a rate of 5 °C min^–1^, held at isothermal conditions for 5 min to erase their thermal history, and then cooled to −50 °C at a rate of 5 °C min^–1^. The heating and cooling cycles were repeated another time starting from −50 °C.

### 4.14 Thermogravimetric Analysis (TGA) of (Co)polymerization Products

The decomposition temperature of all copolymers (**S00–S100**) was assessed by TGA using a TA Instruments TGA550. Samples were dissolved in dH_2_O at high concentration (20 wt %) and lyophilized to obtain a packed solid. These were loaded onto a platinum pan and heated from RT to 1000 °C at a rate of 10 °C min^–1^, starting under a nitrogen atmosphere. At 600 °C, the atmosphere was switched to air to allow pyrolysis.

### 4.15 Calculation of Monomer Concentration Value for the Analysis of Reaction Kinetics and Reactivity Ratios

To analyze the reaction kinetics and reactivity ratios for our copolymerization reactions, we needed to determine the concentrations of **SM** and **DEPMAm** at each time point measured during the reaction. We used the integral values extracted from the ^1^H NMR (700 MHz, DMSO-*d*_6_) spectra of the reaction mixture over time (Figures S9–S11) with the exception of **S100**, which was measured in D_2_O (Figure S12).

For the pseudo-first-order kinetic plots (ln([M]_0_/[M]_*t*_) versus *t*), [M]_*t*_ values were calculated according to
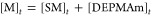
5where [SM]_*t*_ and [DEPMAm]_*t*_ are monomer concentrations at time *t*, given by
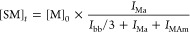


and
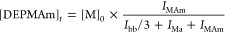


For **SM** and **DEPMAm,** respectively, here, the subscripts “Ma”, “MAm”, and “bb” refer to the methacrylate, methacrylamide, and backbone proton signals. Multiplication by the 1.0 mM starting total monomer concentration yields the current monomer concentration, while the initial concentration is given by the feed ratio. See Section 4.10 for peak positions and the noted supporting figures for spectra.

The mole fraction of remaining **SM** was calculated using the ratio of the methacrylate to methacrylamide integrals:
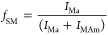
6

### 4.16 Sample Preparation for Rheology Analysis of Copolymer Hydrogels Cross-Linked with PEG-HZ

Stock solutions of different copolymers were prepared in PBS at either 16 wt % (**S75**) or 4 wt % (**S50** and **S25**) while a stock solution of the **PEG-HZ** was prepared containing 100 mM hydrazide functions. The **S50** and **S25** stock solutions were first dissolved in PBS at pH = 5 to facilitate dissolution and subsequently adjusted to pH = 7. Due to the small volumes and high mass content of stock solutions, their densities were approximated by measuring their new volumes. These density values were subsequently used for calculating the volumes of further stock solutions. A density of 1.15 g cm^–3^ was used for **PEG-HZ** and **S75**, while a value of 1.05 g cm^–3^ was used for **S50** and **S25** stock solutions. The final concentration value obtained was then adjusted based on the new volume of the solutions. Values for the added mass, final adjusted volume, and final adjusted concentration can be found below in Table 4.

**Table 4:** Preparation of Stock Solutions for Rheometry

compound	unit *M*_W_^a^ (g mol^–1^)	mass (mg)	*n*_Units_ (mmol)	*C*_Function_^b^ (mM)	volume (μL)	purity	ρ (g cm^–3^)	wt %
**PEG-HZ**	44.64	65.8	1.461	92.24	288	0.95	1.15	22.9
**S75**	227.88	56.0	0.246	71.43	403	1.00	1.15	13.9
**S50**	223.97	16.0	0.071	48.65	420	1.00	1.05	3.8
**S25**	219.54	16.0	0.073	111.40	420	1.00	1.05	3.8

a The average molecular weight of a monomer unit for each polymer is determined by averaging the individual unit molecular weights as a function of chain composition. In the case of **PEG-HZ**, the two terminal hydrazide groups are each considered a monomer unit of different compositions.

b Function refers to hydrazides in **PEG-HZ** and aldehydes in **S75**–**S25**.

Samples were prepared by first mixing the **PEG-HZ** with the PBS necessary to ensure correct final concentrations, mixing the PBS with the copolymer stock solution first left the **PEG-HZ** too concentrated, resulting in more heterogeneous final hydrogels. The dilute **PEG-HZ** was then mixed with the copolymer (**S25**, **S50**, **S75**) stock solution to yield the final concentration specified in Figure 4 (and Table 5), and immediately loaded onto the rheometer. In the case of **S25**, gelation was too fast to premix the solutions and then load them into the rheometer. To overcome this, the diluted **PEG-HZ** solution was suspended from the upper geometry, while the copolymer stock solution was placed on the bottom geometry. Sample loading was performed with an applied rotation to mix the solutions while loading.

**Table 5:** Mass Content and Hydrazide Equivalents for Rheological Characterization of Hydrogel Formulations

copolymer	wt % copolymer	wt % total	conc. Hz (mM)	equiv Hz
**S25**	2	4.70	10.6	0.18
**S50**	2	4.70	10.6	0.40
**S75**	1	2.35	5.3	1.00
**S75**	2	4.70	10.6	1.00
**S75**	3	5.70	10.6	0.67
**S75**	4	6.70	10.6	0.50
**S75**	6	10.0	31.8	1.00

### 4.17 Rheological Analysis of Copolymer Hydrogels Cross-Linked with PEG-HZ

Rheological measurements were performed using a DHR-2 from TA Instruments equipped with a Peltier heating element and solvent trap using a 20 mm cone–plate with an angle of 2.002° at 20 °C. A time sweep was performed for 3600 s to follow cross-linking kinetics at 1 rad s^–1^ and 1% strain, followed by a frequency sweep from 1 to 100 rad s^–1^ at 1% stain, and finally a strain sweep from 0.1 to 1000% strain at 1 rad s^–1^. Final shear moduli values were taken as the average value of the plateau moduli during frequency sweeps. The differential modulus (*K*′ = ∂σ/∂γ) was determined from the strain sweeps by taking the derivative of the oscillation stress with respect to strain. This stiffening index, *m*, was given by the slope to a linear fit of log(*K*′) vs log(σ) (as *K*′ ∝ σ^*m*^) for the final 5 points on the stiffening curve prior to rupture. Similarly, the critical strain (σ_c_) was determined from the intersection of the same linear fit with the plateau modulus.

### 4.18 Sequential Conjugation of Oxime-Functionalized Dyes and RGD to S50

We sequentially ligated aminooxy-conjugated ligands: (1) Aminooxy-CF488A (**Ox-CF488**, ε = 70 × 10^3^), (2) Aminooxy-CF640R (**Ox-CF640**, ε = 105 × 10^3^), and (3) aminooxy-RGD (**Ox-RGD**) onto the **S50** copolymer. In a parallel reaction, we also inverted the order of addition of dyes **1** and **2** (so **2**-**1**-**3**; the sequence of the numbers indicates the order of functionalization).

*Step 1*. The **S50** polymer was predissolved in 3 mL PBS (pH = 5.0) with a final concentration of 25 mg mL^–1^ after volume correction due to increased density (ρ ≈ 1.05 g cm^–3^). Then 0.92 mL of copolymer stock solution (22.8 mg, 29.1 μmol of aldehyde groups, 1.0 equiv) was added into 9.1 mL of PBS (pH = 7.4). The final polymer concentration was 2.3 mg mL^–1^. For the reactions with dyes **1** and **2**, we added 16.8 and 24.3 μL of **1** (2.61 mM, 4.4 × 10^–2^ μmol, 1.5 × 10^–3^ equiv) and **2** (1.81 mM, 4.4 × 10^–2^ μmol, 1.5 × 10^–3^ equiv) stock solutions to the reaction mixtures, respectively. We let the solutions stir at RT for 17 h. Crude mixtures of **S50+1** and **S50+2** were analyzed via GPC after 1:1 (*v/v*) dilution in 0.1 M NaNO_3_. The solutions were then dialyzed in a 3.5 kDa MWCO Snakeskin dialysis membrane against dH_2_O with 2–3 bath changes over 48 h, and finally freeze-dried.

*Step 2*. **S50+1** (16 mg, 20.4 μmol of aldehyde groups) and **S50+2** (19 mg, 24.3 μmol of aldehyde groups) were again predissolved in PBS (pH = 5.0) and diluted to a final concentration of 2.3 mg mL^–1^ in PBS (final pH = 7.4). Subsequently, from the stock solutions, 19.8 μL of **2** (4.2 × 10^–2^ μmol, 1.7 × 10^–3^ equiv) and 16.2 μL of **1** (3.5 × 10^–2^ μmol, 1.7 × 10^–3^ equiv) were added, respectively. We again allowed the solutions to stir at RT for 17 h. The mixture was dialyzed and lyophilized. Purified products were analyzed via GPC.

*Step 3*. **S50+1-2** (11.8 mg, 15.0 μmol of aldehyde groups) and **S50+2-1** (11.6 mg, 14.7 μmol of aldehyde groups) were again predissolved in PBS (pH = 5.0) and diluted to a final concentration of 2.3 mg mL^–1^ in PBS (final pH = 7.4). Subsequently, we added 37.5 and 36.9 μL of **3** from a 20 mM stock solution (0.75 μmol, 0.05 equiv, and 0.74 μmol, 0.05 equiv, respectively) to the solution. We again let the solutions stir at RT for 17 h. Then, the mixture was dialyzed and lyophilized. The final products were collected as green fluffy solids and evaluated via GPC. Final yields (both **S50+1-2-3** and **S50+2-1-3**) were 9.0 mg (39%).

### 4.19 Cyclic Strain Rheometry of an **S75** Hydrogel

Details on the hydrogel preparation and rheological analysis are described in the Supporting Methods.

### 4.20 One-pot Multiconjugation of Oxime-Functionalized Dyes and RGD to **S50**

The **S50** copolymer was predissolved in PBS (pH = 5.0) with a final concentration of 24 mg mL^–1^. Then, 0.746 mL of the polymer stock solution (18 mg, 22.9 μmol of aldehyde groups, 1.0 equiv) was added to 6.95 mL of PBS (final pH = 7.4). The final polymer concentration was 2.3 mg mL^–1^. Subsequently, 15.1 μL of **1** (2.6 mM, 3.9 × 10^–2^ μmol, 1.7 × 10^–3^ equiv), 21.8 μL of **2** (1.8 mM, 3.94 × 10^–2^ μmol, 1.7 × 10^–3^ equiv), and 197 μL of **3** (2.0 mM, 3.9 × 10^–1^ μmol, 1.7 × 10^–2^ equiv) stock solutions were added. The reaction mixture was allowed to stir at RT for 17 h. Crude mixtures were analyzed via GPC after 1:1 dilution in 0.1 M NaNO_3_. The solutions were dialyzed in a 3.5 kDa MWCO Snakeskin dialysis membrane against dH_2_O with 3 bath changes over 48 h, after which the product was freeze-dried, yielding a green fluffy solid (14.5 mg, 81%).

### 4.21 UV–vis Spectroscopy of Sequential and One-Pot Conjugated **S50** Copolymers

UV–vis absorbance spectra of the functionalized copolymers with ligated dyes were recorded on an Agilent Cary 60 UV–vis spectrophotometer in PBS for cell culture using a quartz cuvette (Hellma Analytics, 114F-10-40, Light Path = 10 mm). The absorbance was measured from 250 to 800 nm using a “medium” scan rate (600 nm min^–1^). Samples were measured at a concentration of 2 mg mL^–1^. The dye concentration on the polymer was determined from standard curves, ranging from 10 to 1.25 μM. The reaction efficiency was calculated by dividing the average dye concentration on the copolymer samples by the total concentration of dye added during functionalization (see above).

### 4.22 Hydrogel Formation Using Preconjugated **S50**

Details of the hydrogel formulation of **S50+O–P** using 1.0 equiv of hydrazide cross-linker are described in the Supporting Methods.

### 4.23 Printing Fibers of **S75** and **S25** Hydrogels Using an Aspect Microfluidic Printer

Details of the preparation of the polymer and cross-linker solutions, as well as the printing procedure, are described in the Supporting Methods.

### 4.24 Fluorescence Spectroscopy and FRET Analysis of Dye Displacement in **S25** Copolymer Solutions

Fluorescence emission spectra were recorded on an Agilent Cary Eclipse Fluorescence Spectrophotometer equipped with a multicell holder and a Peltier temperature controller. All measurements were performed at 20 °C in a quartz cuvette by using a medium scan speed with a 2.5 nm excitation slit width. Detector voltages (sensitivity) of 590 and 550 V were used for **Ox-AL647 → Hyd-AL488** (oxime displacing hydrazide) and **Ox-AL647 → Ox-CF488** (oxime displacing oxime), respectively.

In the kinetic study, the fluorescence signals for the donor (**Hyd-AL488** or **Ox-CF488**), FRET, and acceptor (**Ox-AL647**) were monitored for 49 h. The reaction mixture was excited every 60 s at 493 nm to measure the emission at 515 nm (donor) and 680 nm (FRET), and it was excited at 651 nm to measure the emission at 680 nm (acceptor). Beginning and end-point scans were obtained before and after the reaction.

Samples were prepared by initially reacting 1.0 equiv of the donor dye (with respect to aldehyde groups) on the **S25** copolymer. To this end, 5 μL of a 2 mg mL^–1^**S25** stock solution in PBS (0.01 mg, 2.9 × 10^–5^ mmol aldehyde groups), either 8.34 μL of a **Hyd-AL488** ([stock] = 3.51 mM) or 11.2 μL of an **Ox-CF488** ([stock] = 2.61 mM) solution in PBS, and ≈290 μL of PBS were mixed, making up a total volume of 300 μL. The mixtures were left at RT for 19 h. Then, in quartz cuvettes, 97 μL of the reaction mixtures were diluted in 900 μL PBS (final aldehyde and dye concentration = 10 μM). After the addition of a further 1.0 equiv of **Ox-AL647** (2.92 μL, [stock] = 3.33 mM), the kinetics measurement was started. There was a delay in the start time of ≈20 s per quartz cuvette for sample mixing and loading.

### 4.25 Preparation of Hydrogels for FRET/FRAP Measurements

Details on the formulation of various hydrogel (**S25**) formulations are described in the Supporting Methods.

### 4.26 Analysis of FRET and FRAP in **S25** Hydrogels with **Hyd-AL488** and **Ox-AL647**

Details of the equipment, machine settings, and FRET/FRAP protocol are described in the Supporting Methods.

### 4.27 De-cross-Linking of **S25** Hydrogel via Addition of Excess Ox-RGD

Details of the selective decross-linking of an **S25** hydrogel are described in the Supporting Methods.

### 4.28 Culture of Human Dermal Fibroblasts

HDFs were cultured at 37 °C under a 5% CO_2_ atmosphere in Dulbecco’s modified Eagle’s medium (DMEM, Gibco) containing high glucose (4.5 g dm^–3^) and GlutaMax, supplemented with 10% (*v/v*) fetal bovine serum (FBS) and 1% (*v/v*) penicillin/streptomycin (P/S, Gibco). Cells were passaged at approximately 80% confluence and used between passage numbers 8–14.

### 4.29 Evaluation of HDF Viability on **S25–S75** Copolymer Hydrogels

Human dermal fibroblast viability was tested on **S25–S75** hydrogels containing either 0 or 1 mM RGD. Hydrogels were prepared in the same manner as the rheology samples (see above), except that stock solutions of 3.8 wt % for the **S75** ([aldehyde] = 19.6 mM) and 10.5 wt % for the **PEG-HZ** ([hydrazide] = 39.7 mM) were used. We prepared 130 μL hydrogels in Ibidi μ-Plate (96 Well Black Glass Bottom): First, 68.2 μL of **S25–S75** was loaded into the well. Subsequently, a mixture of 33.6 μL of **PEG-HZ** and 31.4 μL of PBS was added. In the gels containing **Ox-RGD** (stock solution: 20 mM), 5 μL of the solution was mixed with **PEG-HZ** and PBS; the **Ox-RGD** volume was subtracted from the PBS volume in this mixture.

Before cell seeding, the gels were incubated with 130 μL of serum-free media supplemented with 1% P/S for 3 h. Then, HDFs (P13) were seeded at a density of 15,000 cells cm^–2^. After 1 day of culture, media was replaced with a solution (1:1 (*v*/*v*) full media:PBS) containing 1 μM calcein, and 2 μM ethidium homodimer-1 was incubated for 30 min at 37 °C, shielded from light. Afterward, the staining solution was replaced with fresh media, and the cells were imaged using an inverted fluorescence microscope (Nikon Eclipse Ti-e) equipped with a live-cell incubator.

### 4.30 Culture of HDFs on Top of S50 Hydrogels and Release to Underlying Substrate via De-Cross-Linking

Details on the hydrogel preparation containing 0–10 mM of RGD (Tables S6 and S7), cell culture, decross-linking protocol, and the lactate dehydrogenase cytotoxicity assay are described in the Supporting Methods.

### 4.31 Statistical Analysis

Statistical analyses were performed using either Origin 2018 SR1 or GraphPad Prism 9.1. The exact statistical test is specified in the figure legends. Fitting of pseudo-first-order kinetics and reactivity ratios was done in Origin 2018 SR1 using the nonlinear curve fitting tool. Data are presented as mean ± standard deviation unless otherwise specified.

## Data Availability

The data that support the findings of this study are openly available in DataverseNL at https://doi.org/10.34894/QF4FBB.

## Supporting Information Available

Additional experimental details, materials, and methods, including copolymerizations, rheology, hydrogel preparation and analysis, fluorescence, and cell studies; homo- and copolymerizations and reactivity rations; ^1^H and ^13^C spectra and kinetic analysis, GPC, FTIR, TGA, DSC, kinetic fitting, rheological data, printing, spectrophotometric data (UV–vis, fluorescence), hydrogel de-cross-linking, and cell studies; solubility tests, additional copolymer property quantification, hydrogel, and stock solution preparation and formulations, strain-stiffening; and monomer conversion and instantaneous copolymer composition (PDF)
