# Supplementary material for: Well-Defined Synthetic Copolymers with Pendant Aldehydes Form Biocompatible Strain-Stiffening Hydrogels and Enable Competitive Ligand Displacement
Source: J Am Chem Soc. 2024 Aug 20;146(35):24330–47. doi: 10.1021/jacs.4c04988 (PMC11378284; doi:10.1021/jacs.4c04988)
Supplement: Supplementary file 1 — ja4c04988_si_001.pdf [file ja4c04988_si_001.pdf]

Supporting Information

Well-defined synthetic copolymers with pendent aldehydes form biocompatible strain-stiffening hydrogels and enable competitive ligand displacement

Ivo A. O. Beeren[1,2,‡], Francis L. C. Morgan[1,2,‡], Timo Rademakers[2], Jurica Bauer[2], Pieter J. Dijkstra[2], Lorenzo Moroni[2*], Matthew B. Baker[1,2,*]

‡ *These authors contributed equally to this work and are listed alphabetically by last name*

[*] *Corresponding authors:m.baker@maastrichtuniversity.nl; l.moroni@maastrichtuniversity.nl*

AUTHOR ADDRESSES

[1]Department of Instructive Biomaterials Engineering, MERLN Institute for Technology-Inspired Regenerative Medicine, Maastricht University, 6229 ER, Maastricht, The Netherlands

[2]Department of Complex Tissue Regeneration, MERLN Institute for Technology-Inspired Regenerative Medicine, Maastricht University, 6229 ER, Maastricht, The Netherlands

KEYWORDS

Dynamic covalent chemistry, aldehyde copolymer, hydrogels, strain-stiffening, ligand displacement, cell delivery

Table of Contents

Supporting Methods

**Method S1**	Deprotection of DEPMAm	S1
**Method S2**	Neutralized RAFT copolymerization of equimolar SM and DEPMAm	S1
**Method S3**	Free radical copolymerization of equimolar SM and DEPMAm	S2
**Method S4**	Hydrogel formation using pre-conjugated **S50**	S3
**Method S5**	Cyclic strain rheometry of an **S75** hydrogel	
**Method S6**	Printing fibers of **S75** and **S25** hydrogels using an Aspect microfluidic printer	S3
**Method S7**	Preparation of hydrogels for FRET and FRAP measurements	S3
**Method S8**	Analysis of FRET and FRAP in **S25** hydrogels with Hyd-AL488 and Ox-AL647	S4
**Method S9**	De-crosslinking of **S25** hydrogel via addition of excess Ox-RGD	S5
**Method S10**	Release of human dermal fibroblasts (HDFs) on top of **S50** hydrogel to underlying substrate via de-crosslinking	S5
**Method S11**	Lactate dehydrogenase (LDH) cytotoxicity assay	S5

Supporting Discussions

**Discussion S1**	Analysis of the homopolymerization of DEPMAm (**S00**)	S7
**Discussion S2**	Observations and comments of the small scale, neutralized RAFT, and free radical copolymerization of equimolar SM and DEPMAm	S8
**Discussion S3**	Supporting discussion of reactivity ratios and copolymer microstructure	S10

Supporting Figures

**Figure S1**	^{1}H NMR (700 MHz, DMSO-d_6) spectrum of DEPMAm	S12
**Figure S2**	^{13}C NMR (176 MHz, DMSO-d_6) spectrum of DEPMAm	S13
**Figure S3**	^{1}H–^{13}C HSQC (700 MHz, DMSO-d_6) spectrum of DEPMAm	S13
**Figure S4**	Time-course GPC traces over 200 min of the homopolymerization of DEPMAm (**S00**)	S14
**Figure S5**	Time-course ^{1}H NMR (700 MHz in DMSO-d_6) spectra monitoring the homopolymerization of DEPMAm (**S00**) over 200 min	S15
**Figure S6**	Time-course ^{1}H NMR (700 MHz in DMSO-d_6) spectra of **S25**	S16
**Figure S7**	Time-course ^{1}H NMR (700 MHz in DMSO-d_6) spectra of **S50**	S17
**Figure S8**	Time-course ^{1}H NMR (700 MHz in DMSO-d_6) spectra of **S75**	S18

**Figure S9**	Time-course ^1H NMR (700 MHz in DMSO-d_6) spectra of **S100**	S19
**Figure S10**	^1H NMR (700 MHz in DMSO-d_6) spectra of crude reaction mixture (at t = 40 min and 180 min) of **NN50**, **N50**, and **FR50**	S20
**Figure S11**	GPC analysis of **N50**, **NN50**, and **FR50**	S21
**Figure S12**	^1H NMR (700 MHz, D_2O) spectrum of purified **N50**, **NN50**, and **FR50**	S21
**Figure S13**	^1H NMR (700 MHz, DMSO-d_6) spectrum of the concentrated crude reaction mixture of the deprotection of DEPMAm	S22
**Figure S14**	^1H NMR (700 MHz, D_2O) spectrum of purified **S25**, with integration values	S23
**Figure S15**	ATR-FTIR spectra of both monomers (SM & DEPMAm), and all (co-)polymers (**S00–S100**)	S24
**Figure S16**	Time-course GPC traces over 200 min of the (co-)polymerization of SM and DEPMAm	S25
**Figure S17**	Thermogravimetric analysis (TGA) of **S00–S100** (co-)polymers	S26
**Figure S18**	Differential scanning calorimetry (DSC) thermographic curves of **S25–S100**	S27
**Figure S19**	Individual monomer conversions over time and supporting plots of the instantaneous copolymer composition (F_A)	S28
**Figure S20**	Pseudo-first order kinetic fit of Ln($[M]_0/[M]_t$) versus time for DEPMAm in the **S75** copolymerization	S29
**Figure S21**	Individual and global fits to the Meyer-Lowry model for the **S25–S75** copolymerization reactions and supporting plot of the instantaneous copolymer composition as a function of overall conversion	S29
**Figure S22**	^1H NMR (700 MHz, D_2O) spectra of **S75** *vs* **S75** mixed with 0.6 equiv w.r.t. aldehyde groups of *O*-ethylhydroxylamine (EH) for 2 days	S30
**Figure S23**	Full time and frequency sweeps of the **S25–S75** hydrogels varying either the copolymer wt% or the copolymer formulation	S31
**Figure S24**	Full strain sweeps of the **S75–S25** copolymer hydrogels, varying either the copolymer wt% or the copolymer formulation	S31
**Figure S25**	Representative plot of normalized differential modulus (K'/K'_{lin}) vs normalized stress (σ/σ_c), and raw K' vs σ for all replicates	S32
**Figure S26**	The strain response, rupture, and recovery, of an **S75** hydrogel subjected to cyclic strain up to 300%	S32
**Figure S27**	Plot of the critical stress (σ_c) and stiffening index (m) as a function of total wt% of the **S25–S75** hydrogels	S33
**Figure S28**	Proof of concept microfluidic printing of **S25** and **S75** with PEG-HZ	S33
**Figure S29**	Absorption intensity vs retention time of **S50** prior to, and post conjugation with either Ox-CF488, Ox-CF640, or both dyes.	S34

Figure S30 ^{1}H NMR (700 MHz in D_2O) spectra of Ox-RGD, and decorated **S50** after purification S35
Figure S31 Absorbance of purified **S50** copolymer with conjugated Ox-CF488, Ox-CF640, and Ox-RGD S35
Figure S32 Gelation kinetics of **S50** with different degrees of pre-functionalization S36
Figure S33 Raw data following the evolution of the donor, acceptor, and FRET signal and ratio over ≈ 49 h S36
Figure S34 UV-Vis spectra of **S25** after pre-functionalization with 1 or 2 equiv small molecule hydrazide/oxime S37
Figure S35 FRET efficiency data of **S25** hydrogels containing conjugated dyes S37
Figure S36 FRAP measurements in **S25** hydrogels to indicate changes in dye diffusion as result of displacement S38
Figure S37 Bulk de-crosslinking of **S25** hydrogel via addition of excess Ox-RGD S39
Figure S38 A 2 wt% **S25** polymer was crosslinked using PEG-HZ in a 96 well plate S39
Figure S39 Proof-of-concept study using competitive displacement upon addition of an aminooxy small molecule to de-crosslink **S50** hydrogels and release cells to the tissue culture plastic S40
Figure S40 LDH cytotoxicity assay of HDFs seeded on **S50** hydrogels containing 0–10 mM Ox-RGD S41
Figure S41 Live and dead staining image of HDFs on TCP after 24 h S41

Supporting Tables

Table S1 Different solvents and conditions tested to dissolve the product obtained after dialysis and lyophilization of the **S00** reaction mixture S42
Table S2 Summary of conversion and copolymer characteristics for the small scale neutralized RAFT and free radical **S50** copolymerizations S42
Table S3 Screening of mass content and crosslinker equivalent to determine possible hydrogelation regimes using **S75** S42
Table S4 Gelation onset times and plateau moduli for the **S25–S75** hydrogel formulations at different mass content and functionalization S43
Table S5 Summary of critical stresses and associated stiffening indices S43
Table S6 Preparation of typical stock solutions for hydrogels used in the HDF release study S43
Table S7 Hydrogel formulations for the HDFs release study S43

Supporting Equations

Equation S1 Monomer conversion S44

Supporting methods and schemes

Deprotection of DEPMAm

First, 100 µL of *N*-(3,3-diethoxypropyl)methacrylamide (DEPMAm) was dissolved in 1.9 mL 1:1 dH_2O:1,4-dioxane (*v/v*). The pH was adjusted to 4 and the solution was left to stir for 19 h at RT. The solvents were removed *in vacuo* and a white solid was obtained. However, the product was poorly soluble in both aqueous and organic solvents; the addition of DMSO-d_6 and subsequent ^{1}H NMR evaluation revealed that both the deprotected and protected methacrylamides were present in the soluble fraction at but isolating the deprotected aldehyde was not pursued further (**Discussion S1**).

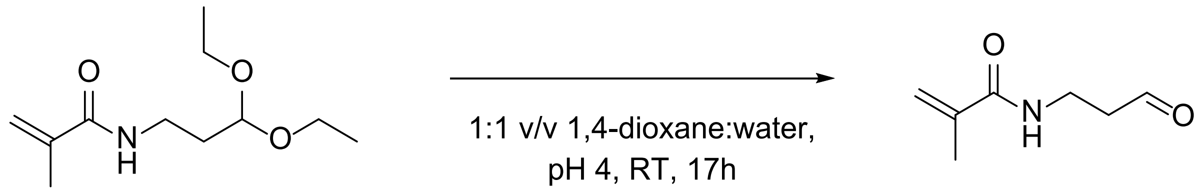

Scheme S1. Acetal deprotection conditions for DEPMAm.

Neutralized RAFT copolymerization of equimolar SM and DEPMAm

A RAFT copolymerization of 3-sulfopropyl methacrylate potassium salt (SM) and DEPMAm was performed as described in the main text on a smaller scale according to the scheme below: 1.80 mL instead of 4.08 mL. We maintained 4-Cyano-4-(phenylcarbonothioylthio)pentanoic acid (CPPA) as the chain transfer agent (CTA), and 4,4'-Azobis(4-cyanopentanoic acid) (ACVA) as the initiator. The total monomer concentration, CPPA concentration, and ACPA concentration were kept constant at 1.0 M, 4.0 mM, and 1.1 mM, respectively.

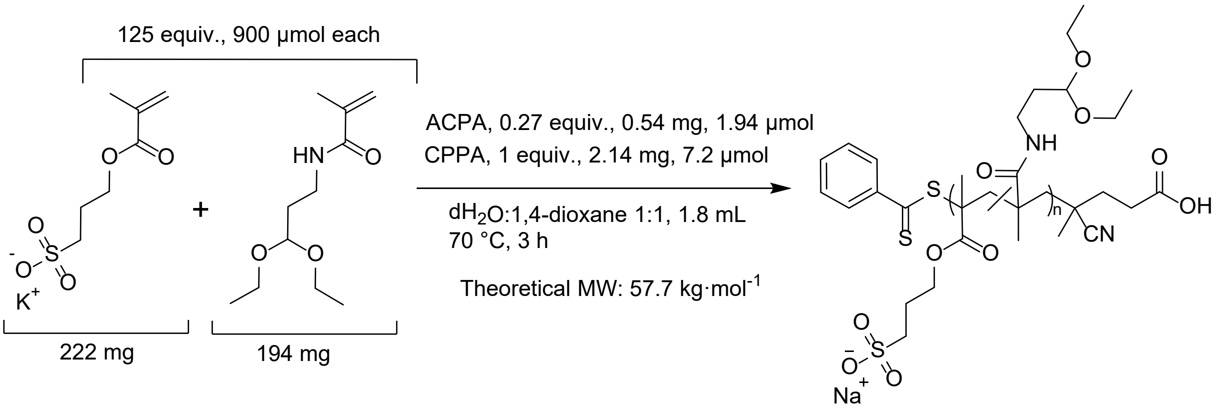

Scheme S2. Reaction scheme for the small-scale, neutralized RAFT copolymerization of equimolar SM and DEPMAm.

The reaction was prepared as described in the main text. In parallel, a second copolymerization was performed at the same scale but with slight differences in preparation to give a neturalized starting reaction mixture. The ACPA and CPPA stock solutions were combined and their initial pH of 3.7 was raised to 5 using 0.5 M NaOH. Following addition of the two monomer solutions (the DEPMAm is slightly basic), the final pH was adjusted to 6.5 as given by the pH meter (mixed solvent systems complicate rigorous definition of pH).[1] The neutralized reaction mixture was then degassed via bubbling dry N_2 gas through the solution for 45 min at RT. Finally, the flask was immersed in an oil bath at 70 °C for 3 h while maintaining positive dry N_2 pressure. The same purification consisting of dialysis of the crude reaction mixture against 0.1 M HCl, 50 mM NaCl (periodically neutralized with saturated $NaHCO_3$), 25 mM NaCl, and finally dH_2O was performed prior to lyophilization to yield pale pink to white fluffy solids (depending on residual CTA content). These polymers – obtained from smaller scale reactions – are denoted as **N50** (104 mg, 25% yield) and **NN50** (107 mg, 26% yield) for the neutralized and non-neutralized reactions, respectively.

Free radical copolymerization of equimolar SM and DEPMAm

The free radical copolymerization was performed in parallel with the neutralized and non-neutralized (control) reaction under the same conditions as the non-neutralized control with one exception: The free radical polymerization did not contain any chain transfer agent. Following purification (as for **N50** and **NN50**) and lyophilization, a fluffy white solid was obtained. The obtained polymer is denoted as **FR50** (130 mg, 31% yield).

Hydrogel formation using pre-conjugated **S50**

The conjugated **S50** copolymer described in the main text (*One-pot multi-conjugation of oxime-functionalized dyes and RGD to **S50***) was dissolved at 4 wt% (4 mg in 100 µL) in phosphate buffered saline (PBS). Rheometry samples were prepared and measured as described in the main text, with the final formulation containing 1.5 wt% S50, 1 equiv 4-arm poly(ethylene glycol) hydrazide (w.r.t. total aldehydes, [hydrazide] = 18.8 mM, M_w = 5000 g·mol^{-1}).

In addition, **S50** copolymers were prepared with various degrees of pre-conjugation with *O*-ethylhydroxylamine, namely targeting 0.2, 0.6, and 0.9 equiv w.r.t. available aldehdyes. To this end, we prepared a 4 wt% solution of **S50** and a 250 mM solution of *O*-ethylhydroxylamine in PBS. Then, we mixed polymer, PBS, and the *O*-ethylhydroxylamine solutions in an Eppendorf tube, vortexed, and left the solutions at RT for 3–4 h for each of the targeted degrees of pre-conjugation.

Rheometry samples were then prepared by adding **PEG-HZ** from a stock solution ([hydrazides] = 41.2 mM) to obtain a 2 wt% **S50** solution ([total initial aldehyde] = 25.5 mM), and measured as described in the main text. For samples treated with 0.2 and 0.6 equiv during pre-conjugation, 0.4 equiv hydrazide functions were added, where for 0.9 equiv pre-conjugation, 0.1 equiv hydrazides were added.

Cyclic strain rheometry of an **S75** hydrogel

Following *in situ* hydrogel formation as described in section 4.16, a 1.9 wt% **S75** hydrogel (1 equiv hydrazide from **PEG-HZ**) was subject to a series of cyclic strain sweeps at 1 rad·s^{-1} from 1–10%; 10 –1%; 1–30%; 30 –1%; 1–50%; 50 –1%; 1–100%; 100 –1%; 1–200%; 200 –1%; 1–300%; 300 –1%.

Printing fibers of **S75** and **S25** hydrogels using an Aspect microfluidic printer

Stock solutions of 8.0 wt% **S75** ([aldehyde] = 40 mM), 9.5 wt% **S25** ([aldehyde] = 280 mM), and 13.2 wt% homobifunctional poly(ethyleneglycol) hydrazide (PEG-HZ, [hydrazide] = 50 mM, M_w = 5000 g·mol^{-1}) were used for this experiment. The **S75** and PEG-HZ solutions were loaded in a microfluidic bioprinter (RX1 Aspect Biosystems, Canada). We used the DUO CORE-SHELL print head (Aspect Biosystems), which allowed the inflow of two materials into the printing nozzle. The **S75** solution was in the 'Core A' channel and the PEG-HZ was in the 'Shell A' channel. A pressure of 40 mbar was applied to both the crosslink and polymer solution to enable inflow into the print head. We fabricated a rectangular, single structure (width = 40

mm) using a translation speed of 10 mm·min^{-1}. The **S25** and the PEG-HZ were printed used the syringe pumps to enable inflow into the print head. The 'Buffer' channel contained PBS and flowed at a speed of 100 μL·min^{-1}, while 'Core A' contained the **S25** and flowed at a speed of 200 μL·min^{-1}. Finally, in 'Shell A', we added the PEG-HZ, which flowed at 50 μL·min^{-1}.

Preparation of hydrogels for FRET and FRAP measurements

Stock solutions in PBS of **S25** (3.8 wt%, 110 mM aldehyde) and PEG-HZ (5 wt%, 20 mM hydrazide) were prepared and adjusted to pH = 7.4 using 0.5 M NaOH. Stock dye solutions of aminooxy-Alexa Fluor^{TM}647 (Ox-AL647, 111 mM) and hydrazide functionalized Alexa Fluor^{TM}488 (Hyd-AL488, 111 mM) were also prepared. From these stock solutions, 20 μL droplets composed of 1 wt% **S25**, 0.1 equiv PEG-HZ (0.2 equiv hydrazide), 0.001 equiv dye (28 μM), and a total mass content of 2.5 wt% were formed on parafilm to prevent spreading. After ≈20 min, droplets were transferred to a 4-quadrant imaging petri dish. We prepared 3 different formulations and allocated them quadrant positions in the petri. **Hydrogel A** contained the donor dye (Hyd-AL488), **Hydrogel B** contained the acceptor dye (Ox-AL647), and **Hydrogel C** contained both dyes (positive control). Stock solutions of Ox-AL647 and Hyd-AL488 at 5.0 μM were added to their complementary gels (**Hydrogel A** and **Hydrogel B**, respectively), when performing fluorescence resonance energy transfer/fluorescent recovery after photo-bleaching (FRET/FRAP) experiments. PBS was added to **Hydrogel C** as a swelling control.

Analysis of FRET and FRAP in S25 hydrogels with Hyd-AL488 and Ox-AL647

FRET and FRAP experiments were performed on an inverted SP8 laser scanning confocal microscope (Leica), equipped with a white-light laser (WLL) and a 100x oil objective (Leica HC PL APO CS2 100x/1.40 OIL). Imaging was performed at a frame size of 512x512 pixels, and a frame rate of 0.865 s. FRET imaging was performed using the FRET AB module, which measures FRET efficiency through acceptor photobleaching:

$$\text{Efficiency} = \frac{D_{\text{Post}} - D_{\text{Pre}}}{D_{\text{Post}}}$$

Where D_{Pre} and D_{Post} refer to the donor fluorescent intensity pre- and post-photobleaching. Donor (Hyd-AL488) and acceptor (Ox-AL647) were measured pre- and post-bleach; donor signal was acquired between 500–550 nm, with the WLL tuned to 488 nm at 8% intensity; acceptor signal was acquired between 675–725 nm, with the WLL tuned to 647 nm at 7% intensity. During bleaching, the acceptor was bleached using the WLL tuned to 647 nm

at 100% intensity for 120 s. Within the bleaching ROI, the intensity of the donor and acceptor were measured pre- and post-bleach. Gels without the donor or acceptor were used as controls. Measurements were first taken on **Hydrogel A** and **Hydrogel C** as prepared; the former as a background control (no acceptor) and the latter as a positive control (equilibrated equimolar quantities). **Hydrogel A** was covered with stock 5.0 µM Ox-AL647, while **Hydrogel C** was covered with PBS. Measurements were then performed to evaluate the change in FRET signal in 3D hydrogels. Acquisition was performed at 5 different spots per gel, at the edge (behind the diffusion front), with 2 independent replicates ($N = 2$, $n = 5$).

FRAP imaging was done using the FRAP module with FRAP booster and 'Zoom during bleach' on a Ø15 µm bleaching area, with a bleaching time of 120 s at maximal laser intensity. Prior the bleaching, three pre-bleach images were recorded, and recovery was followed for 360 s after the bleaching time had ended. For image acquisition pre- and post-bleach, Hyd-AL488 was acquired between 505–560 nm, with the WLL tuned to 488 nm at 1.5% intensity, while Ox-AL647 was acquired between 660–720 nm, with the WLL tuned to 647 nm at 1.5% intensity. Measurements were first taken on **Hydrogel A–C** as formed ('preADD', $n = 2$). **Hydrogel A** and **Hydrogel B** were covered with stock 5.0 µM solution of their complementary dye (donor or acceptor), while **Hydrogel C** was covered with PBS. **Hydrogel C** also serves as a control for swelling through the addition of PBS. Measurements were then performed to study the effect of adding an imine competitor in 3D hydrogels. Afterwards, the ROI was reduced in size to Ø 12–13 µm for analysis to improve the signal to noise ratio. Then, the fluorescence intensities after bleaching were scaled to 1.0 before following the fluorescent recovery over time. Measurements were done in quadruplicate ($n = 4$).

De-crosslinking of S25 hydrogel via addition of excess Ox-RGD

To demonstrate the displacement of crosslinks we prepared two 50 µL droplets using the **S25** copolymer (3.8 wt% stock, 111 mM aldehydes) and homobifunctional PEG-HZ (5 wt% stock, [hydrazides] = 19 mM hydrazides). Hydrogels (50 µL) were prepared at 1 wt% **S25** with 0.20 equiv hydrazides and 0.001 equiv Ox-AL647 – corresponding to a final dye concentration of 28 µM. Once formed, hydrogels were transferred to a glass vial and covered with 800 µL of either PBS or 4.1 mM Aminooxy-RGD (Ox-RGD, ≈ 2 equiv w.r.t. aldehdyes). Photos were taken to follow the evolution of each gel at 0 h, 5 h and 24 h.

Release of HDFs on top of **S50** hydrogel to underlying substrate via de-crosslinking

Stock solutions of **S50** and PEG-HZ were prepared according to **Table S6**. All stock solutions were sterile filtered before use. We prepared 100 μL hydrogels containing 0, 2.5, 5.0, and 10 mM of Ox-RGD in Ibidi black 96-well flat-bottom plate, according to **Table S7**. Gels were formed by first loading 52.5 μL of the **S50** stock solution in the wells. Subsequently, a mixture of PEG-HZ, PBS and Ox-RGD was added, and hydrogels were left to form overnight at 4 °C. Prior to cell seeding, hydrogels were pre-incubated with serum-free media for 4 h. human dermal fibroblasts (HDFs, passage 14) were seeded at a density of 15000 cells·cm^{-2}. After 3 days, the morphology of the cells was assessed using brightfield microscopy.

After assessing morphology (at day 3), the media was changed. We added 10 μL of a 250 mM *O*-ethylhydroxylamine stock solution (1.0 equiv w.r.t. aldehydes, 25 mM final concentration) and ≈ 90 μL of full medium to de-crosslink the hydrogels. The bottom of the wells were imaged using brightfield microscopy after 3 more days of culture (6 days total).

Lactate dehydrogenase cytotoxicity assay

After 24 h and 48 h, we determined the LDH activity of the HDFs seeded on the top of the hydrogels according to manufacturer's protocol of the CyQUANT™ LDH Cytotoxicity Assay Kit (Invitrogen). Briefly, 50 μL of the media was collected and transferred to an Ibidi black 96-well flat-bottom plate. Subsequently, we added 50 μL of the 'Reaction Mixture' of the kit to each well and left the plate at RT for 30 min, protected from light. Next, we added 50 μL of 'Stop Solution' to each well. Then, the absorbance at 490 nm and 680 nm was measured. To determine the LDH activity, the absorbance at 680 nm was subtracted from the absorbance at 490 nm. To calculate the cell viability, the data is corrected for the spontaneous LDH release (HDFs seeded on tissue culture plastic) and the maximum LDH release. The latter is determined by lysing cells seeded on tissue culture plastic by adding 10 μL of 10X lysis buffer to 90 μL of media. Finally, the cell viability was calculated, according to the following formula:

$$\text{Cell viability (\%)} = 1 - \frac{\text{LDH}_{\text{Sample}} - \text{LDH}_{\text{Spontaneous}}}{\text{LDH}_{\text{Max}} - \text{LDH}_{\text{Spontaneous}}}$$

Supporting Discussions

Discussion S1. Analysis of the homopolymerization of DEPMAm (S00).

Compared to the **S25–S75** copolymerization and S100 homopolymerization, the S00 had the lowest monomer conversion (49%) and highest dispersity (1.32). The dispersity of RAFT polymerization is generally between 1.1 and 1.3; the controlled nature of a RAFT polymerization keeps dispersity low. The large dispersity of 1.32, but particularly, the linear increase over time (**Figure S4**) indicate a loss of control over the radical as the reaction progresses. Despite the apparently successful crude synthesis of **S00** (**Figure S5**), the calculated monomer conversion (49% via NMR) is much too high to be consistent with the observed 8.5 kg·mol^{-1} polymer (**Table 1**); either the polymerization is terminated early leading to a population of short chains, or there are competing side reactions.

Analysis of the crude ^{1}H NMR spectrum obtained at 200 min revealed the presence of multiple unidentified peaks, which is indicative of side reactions (**Figure S5**). Compared to the other polymerizations, the side reactions were much more apparent after 200 min. Considering the amide peaks, instead of steady increase as the methacrylamide is incorporated into the desired copolymer, we observe the appearance of two new distinct amide peaks, indicating different amide populations. Similarly, we observed the vinylic proton signals split into three pairs. These results indicated that either the monomer is degrading or side reactions are occurring that slightly change the chemical shift of the amide or vinylic protons. With at least three (potentially) polymerizable methacrylamide species, the presence of multiple polymeric species was therefore unsurprising. After following our purification and lyophilization protocol, many attempts were made to solubilize **S00** in order to elucidate the nature of the reaction products, but these were ultimately unsuccessful (**Table S1**).

Discussion S2. Observations and comments on the small scale, neutralized RAFT, and free radical copolymerization of equimolar SM and DEPMAm

We performed a copolymerization in a neutralized reaction mixture in order to investigate the effect of pH on in-situ aldehyde deprotection. In addition, a free radical polymerization (at pH = 5) was performed to assess if the in-situ aldehyde deprotection interfered with the copolymerization, as free aldehydes are generally reported to be incompatible with radical polymerizations. These syntheses were smaller scale analogues of the **S50** copolymerization, and the methods are described in the **Supporting Methods**.

By shifting the pH up to 6.5, we observed no significant change in the aldehyde deprotection or obtained product (**NN50**) compared to performing the reaction of pH = 5.0 (**N50**, as was done in the main text). However, we did notice that a reduction in the scale of the polymerization reduced the overall incorporation of DEPMAm into the copolymer, as well as both the M_n and dispersity (**Table S2**). In contrast, the free radical polymerization product (FR50) precipitated between 1–2 h. Despite precipitating out before the end of the reaction time, FR50 has a much higher M_n compared to the RAFT products, but also possesses a larger, bimodal molecular weight distribution (**Figure S11**). This suggests that the solubility of the copolymer is dependent on chain length in this solvent system, as chain length is known to affect solubility.[2,3] Notably, the free radical copolymer product is still highly pure according to ^{1}H NMR spectra, contains a higher fraction of aldehydes compared to the RAFT copolymers, and was equally easy to purify via dialysis and lyophilization. These results indicate a surprising compatibility of free aldehydes to undergo RAFT copolymerization in mixed H_2O:1,4-dioxane solvent system, and suggest that these reaction conditions may allow more general copolymerization of unprotected aldehyde monomers in the future.

To investigate this hypothesis, we attempted to deprotect DEPMAm (to recover *N*-(3-oxopropyl)methacrylamide) in order to prepare a fully deprotected aldehyde monomer. For this reaction, we chose to replicate the reaction conditions (1:1 dH_2O:1,4-dioxane (*v/v*), pH = 4.5–5, 70 °C) used in our polymerizations. Unfortunately, after removing the solvent, the obtained white product remained insoluble even leaving a sample of the resulting white solid in 0.1 M HCl for one week did not lead to increase solubility. If aldehyde condensation reactions were responsible for the observed insolubility, we would expect aqueous acidic conditions to facilitate the retro-aldol condensation. A portion of the product was then allowed to stand in DMSO-d_6 to extract the soluble fraction for analysis. In **Figure S13**, we observe that the soluble fraction contains the desired product, with the appearance of an aldehyde peak and corresponding disappearance of ethanol peaks, indicating complete deprotection. However, the

secondary reactions predominantly forming the insoluble fraction prohibited the isolation of any useful quantity of *N*-(3-oxopropyl)methacrylamide.

Discussion S3. Supporting discussion of reactivity ratios and copolymer microstructure

A pure gradient copolymerization can be defined by $\gamma = r_A \cdot r_B = 1$ (with $r_A \neq r_B$), where the last monomer unit added to a growing chain has no influence on the subsequent monomer that will be added; the relative rates of incorporation of monomer A or B remain constant. This can be seen as:

$$1 = r_A r_B = \frac{k_{AA}}{k_{AB}} \frac{k_{BB}}{k_{BA}}$$

$$k_{AB} k_{BA} = k_{AA} k_{BB}$$

and

$$\frac{k_{AB}}{k_{BB}} = \frac{k_{AA}}{k_{BA}}$$

Where the relative rate of monomer B being added to a chain ending with either monomer A or B is the same as monomer A.

Different ranges of γ, r_A, and r_B correspond to different polymerization regimes (alternating, blocky, etc.) which are also discussed further by Lynd et al.[4] Though to briefly provide some copolymerization regimes:

- As γ tends towards 0 with $r_B \ll r_A$ (or vica versa), one monomer is increasingly preferentially added to the chain ends, tending towards to a microstructure that contains ideal asymmetric alternations (single units of one monomer separated by segments of the other).
- As γ tends towards 0 with $r_B \approx r_A$ (i.e. both r_A and r_B are increasingly small), we tend towards a symmetrically alternating copolymer, where a chain terminating in one monomer unit will exclusively add to the co-monomer and *vice versa*.
- When $\gamma \neq 1$ with $r_B \neq r_A$, we produce a gradient: alternating segments of A and B with varying lengths. As γ becomes >> 1, we tend towards blockier copolymers, where the size of the blocky segments increases.

Determined reactivity ratios for a copolymerization reaction can be used in a variety of ways. Using **Equation S2**, we can determine the instantaneous copolymer chain composition (F_A) as a function either of the instantaneous feed ratio ($f_A(t)$, **Figure S19B**) or of the total monomer conversion (**Figure S19C**). These types of plots are of particular use for

understanding how the composition of the copolymer chain varies over time from the beginning to the end of the synthesis.

Considering **Figure 19B**, the **S25**, for example, exhibits a constant gradual increase in aldehyde content with conversion compared to the near constant composition of the **S75** followed by a steep increase once the majority of SM is consumed. Similarly, **Figure S19C** shows us that for the **S50**, the initial chain composition is ≈ 85% SM**,** yet ends at ≈ 35% SM. While beyond the scope of the current work, for systems that exploit difference monomer ratios to vary solubility, self-organization, or other phenomenon relying on the physical properties of a copolymer, this type of analysis is valuable.

Supporting Figures

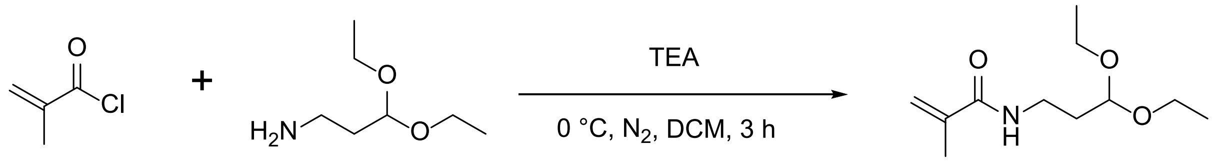

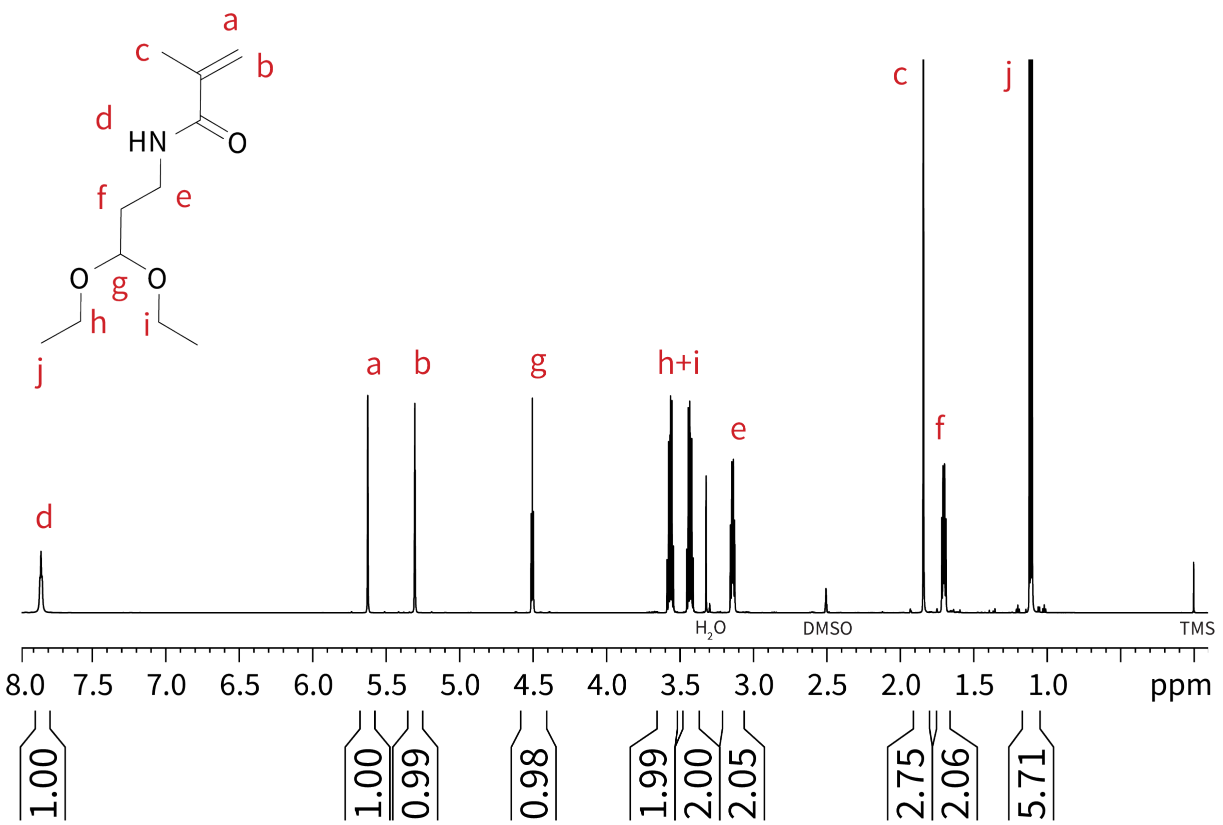

Figure S1. Top) Synthesis scheme for DEPMAm. Bottom) ^{1}H NMR (700 MHz, DMSO-d_6) spectrum of DEPMAm, synthesized according to the scheme above.

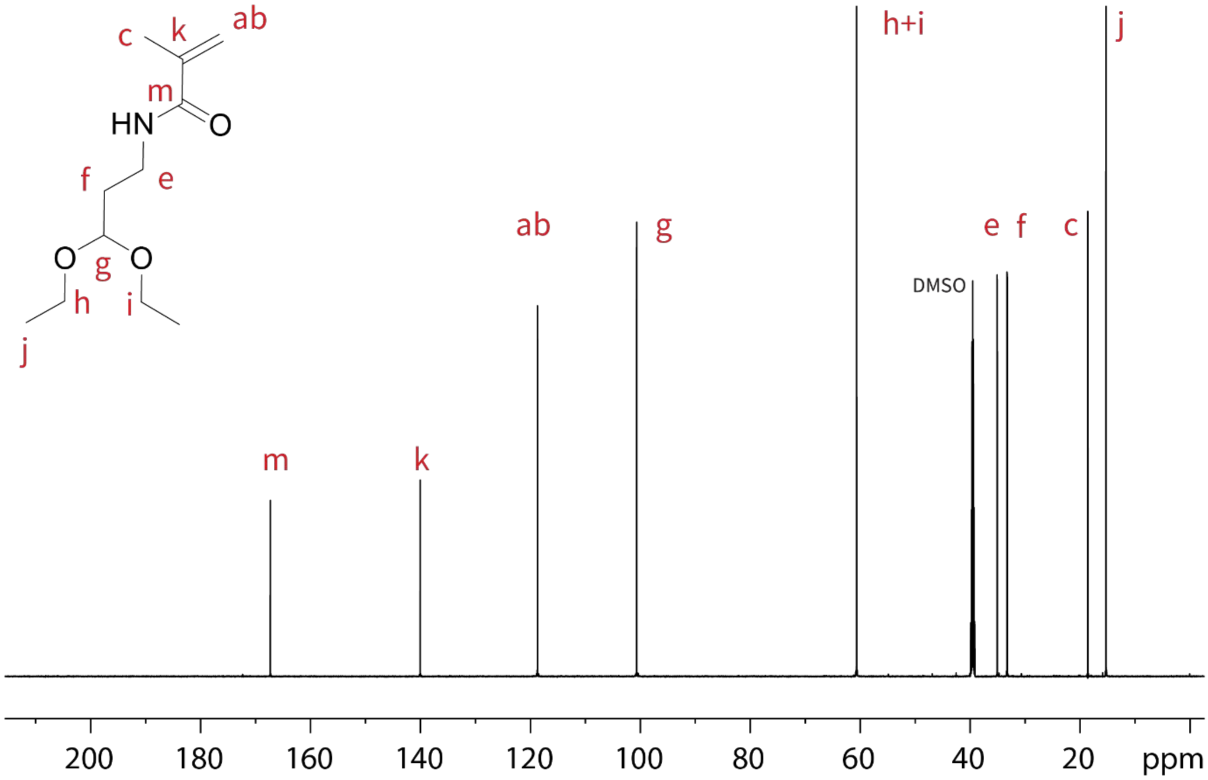

Figure S2. ^{13}C NMR (176 MHz, DMSO-d_6) spectrum of DEPMAm.

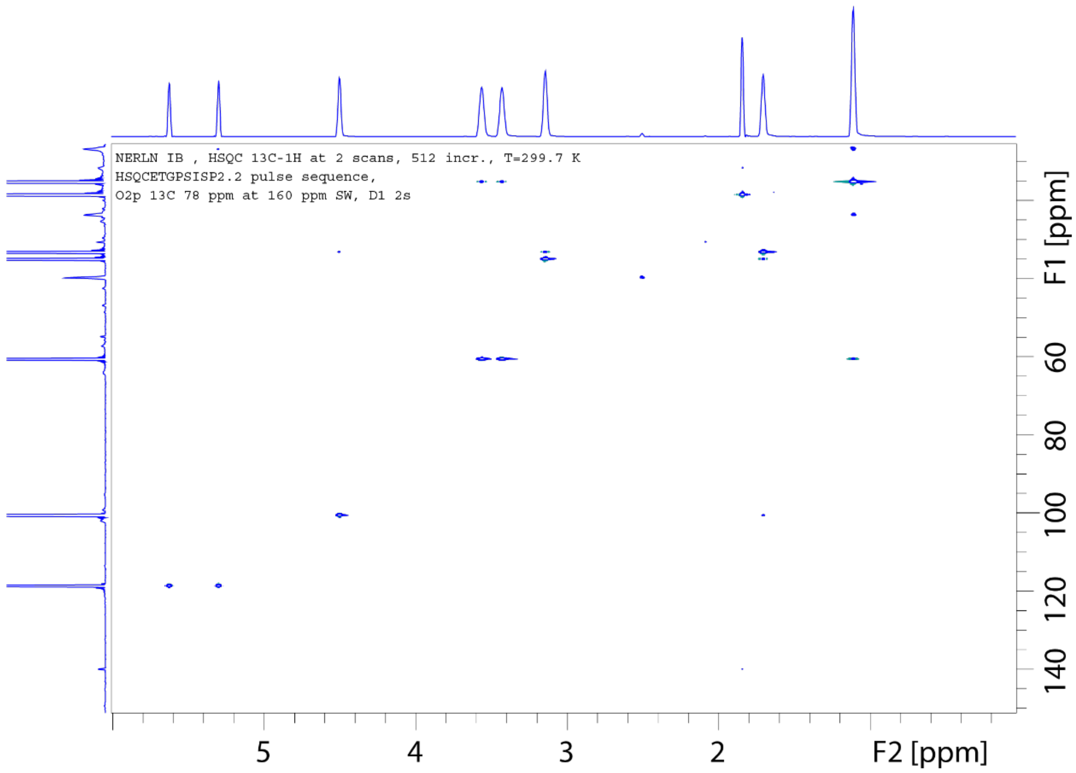

Figure S3. ^{1}H (700 MHz)–^{13}C HSQC (176 MHz, DMSO-d_6) spectrum of DEPMAm.

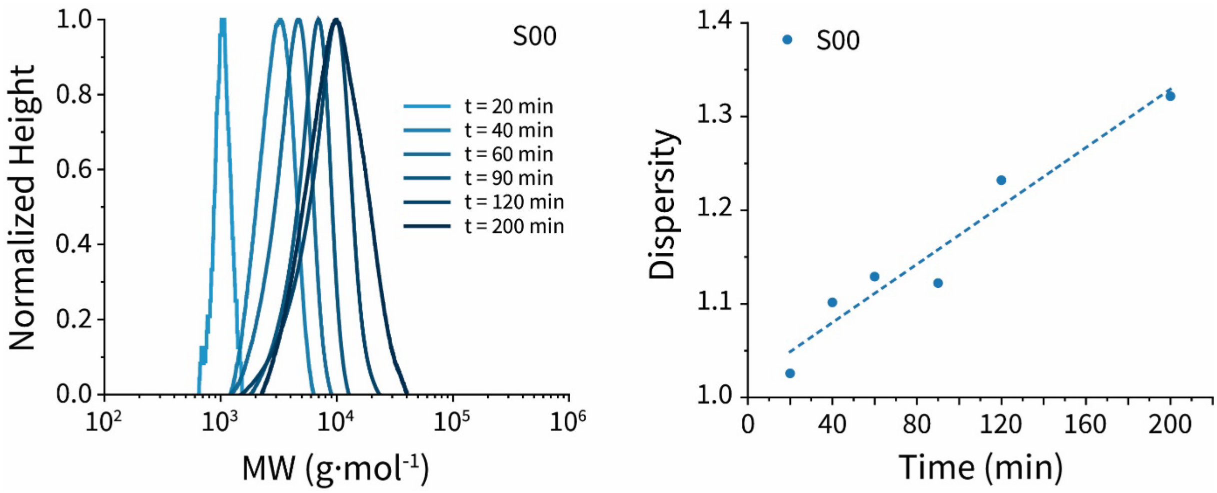

Figure S4. (Left) Time-course GPC traces over 200 min of the homopolymerization of DEPMAm. (Right) The corresponding dispersity over time for the same homopolymerization showing a consistent increase inconsistent with a controlled radical polymerization; a linear fit has been added for visual clarity.

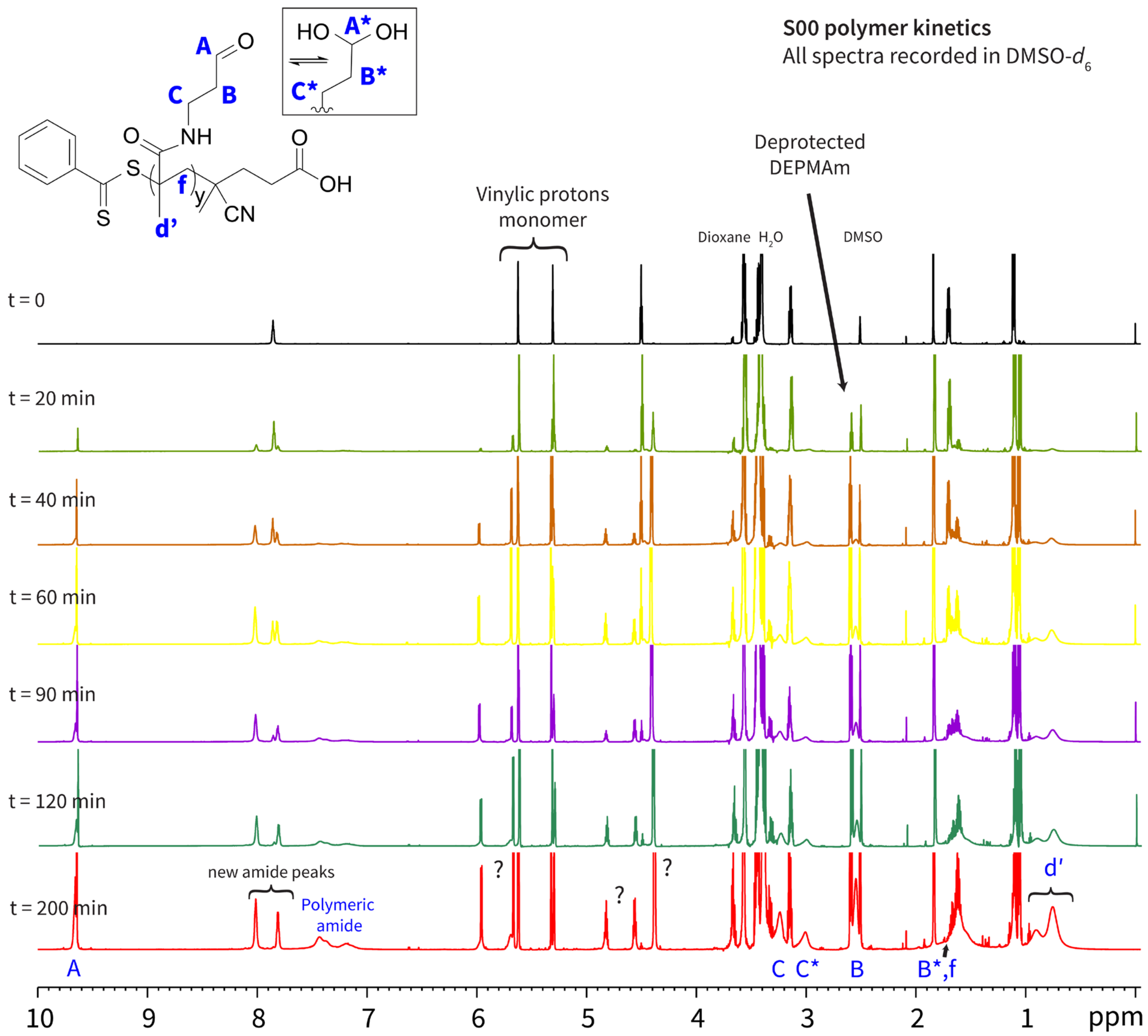

Figure S5. Time-course ^{1}H NMR (700 MHz in DMSO-d_6) spectra monitoring the homopolymerization of DEPMAm (**S00**) over 200 min.

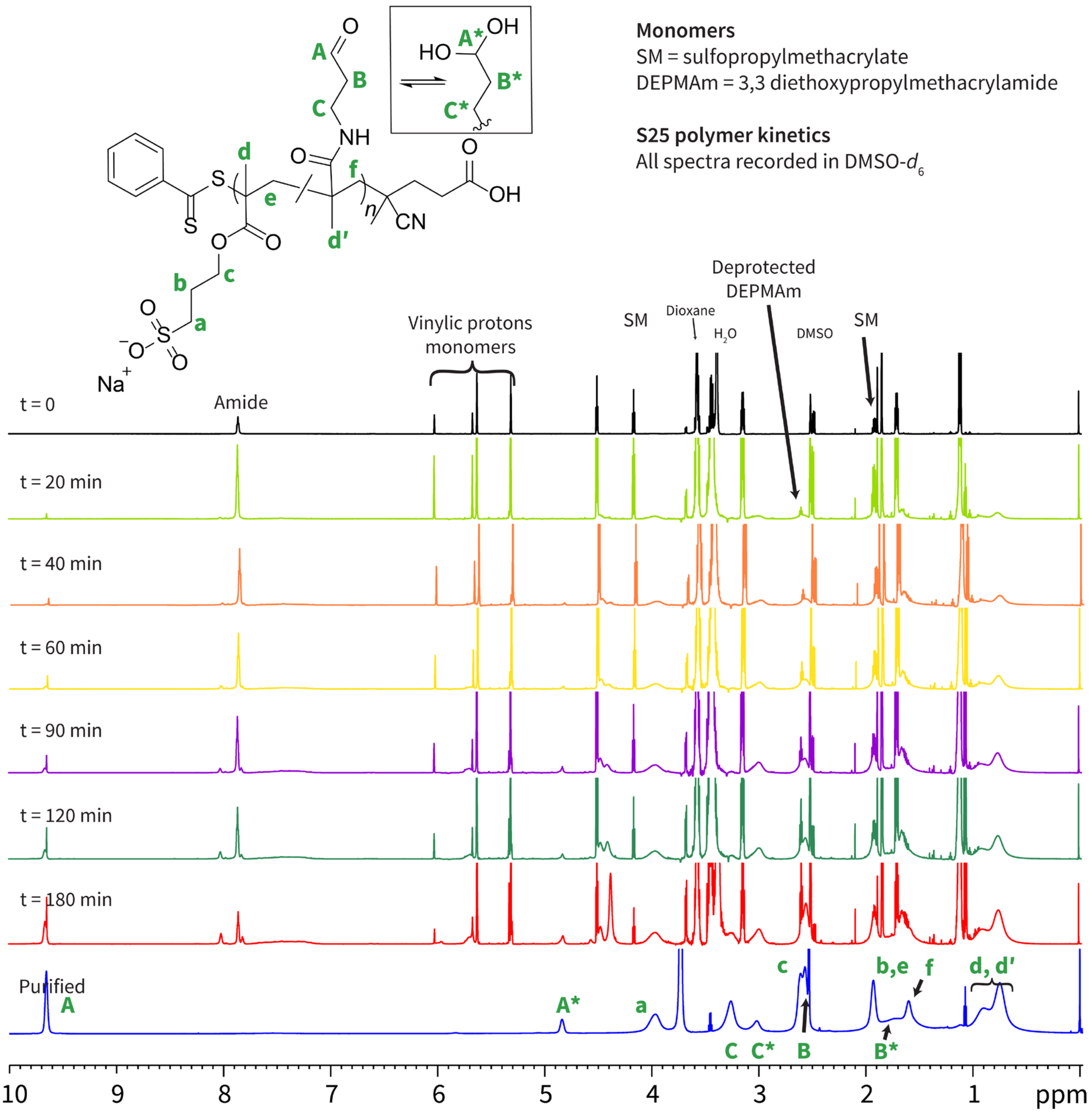

Figure S6. Time-course ^1H NMR (700 MHz in DMSO-d_6) spectra monitoring the copolymerization of SM and DEPMAm with a feed ratio of 25:75 mol % (**S25**), respectively. Based on the ratio of the newly apparent ethyl peak of ethanol (≈ 1.06 ppm, first visible after the start of the reaction) to the methyl peak of the acetal protecting group (≈ 1.11 ppm), we estimated the amount of *in situ* deprotection to be ≈ 60% after 180 min. To obtain the purified spectra in DMSO, the polymer was pre-dissolved in D_2O (≈ 10% of the total volume), which led to the disappearance of the amide peak and acetal formation with the free aldehyde.

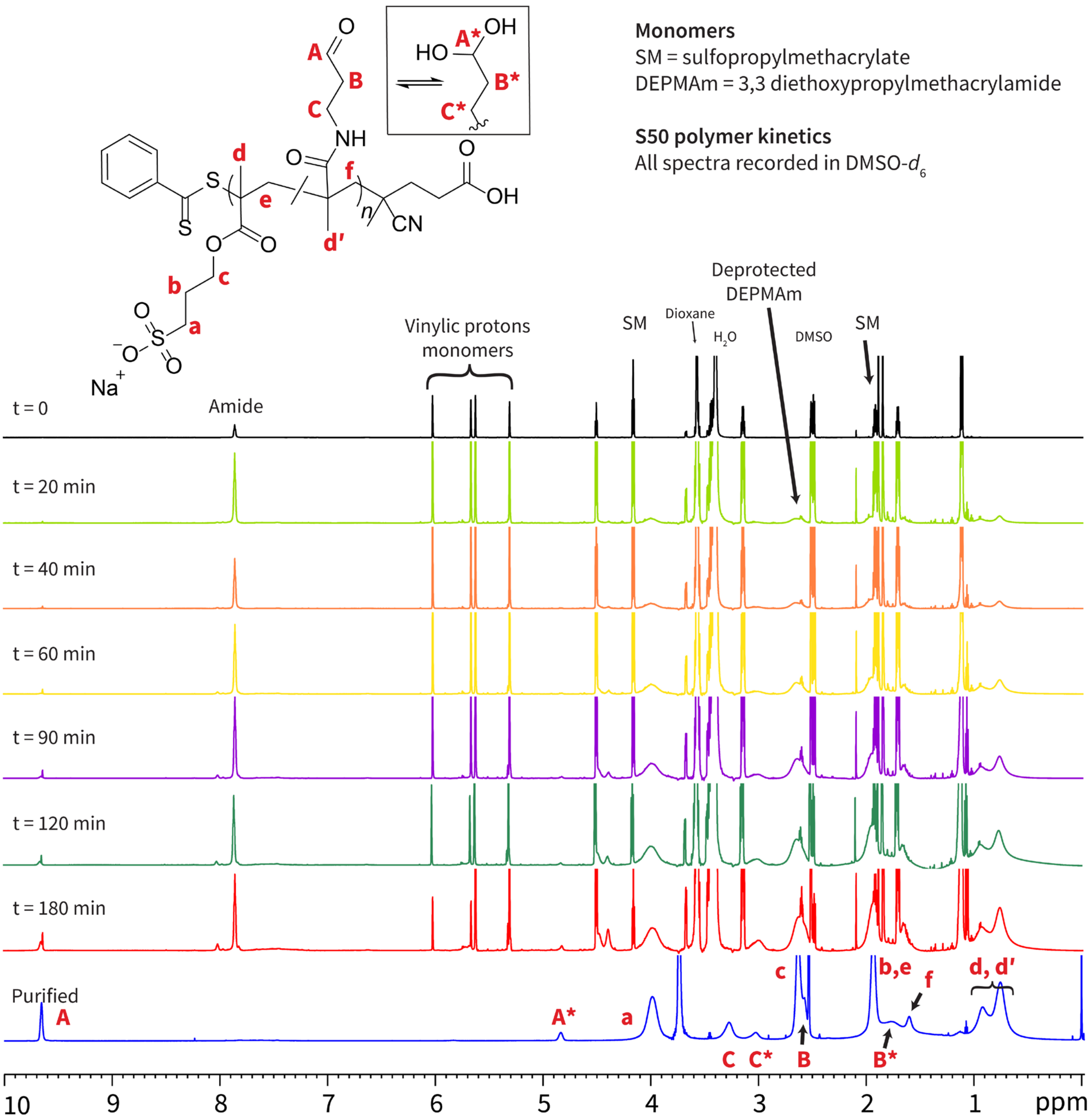

Figure S7. Time-course ^{1}H NMR (700 MHz in DMSO-d_6) spectra monitoring the copolymerization of SM and DEPMAm with a feed ratio of 50:50 mol % (**S50**), respectively. Based on the ratio of the newly apparent ethyl peak of ethanol (≈ 1.06 ppm, first visible after the start of the reaction) to the methyl peak of the acetal protecting group (≈ 1.11 ppm), we estimated the amount of *in situ* deprotection to be ≈ 30% after 180 min. To obtain the purified spectra in DMSO, the polymer was pre-dissolved in D_2O (≈ 10% of the total volume), which led to the disappearance of the amide peak and acetal formation with the free aldehyde.

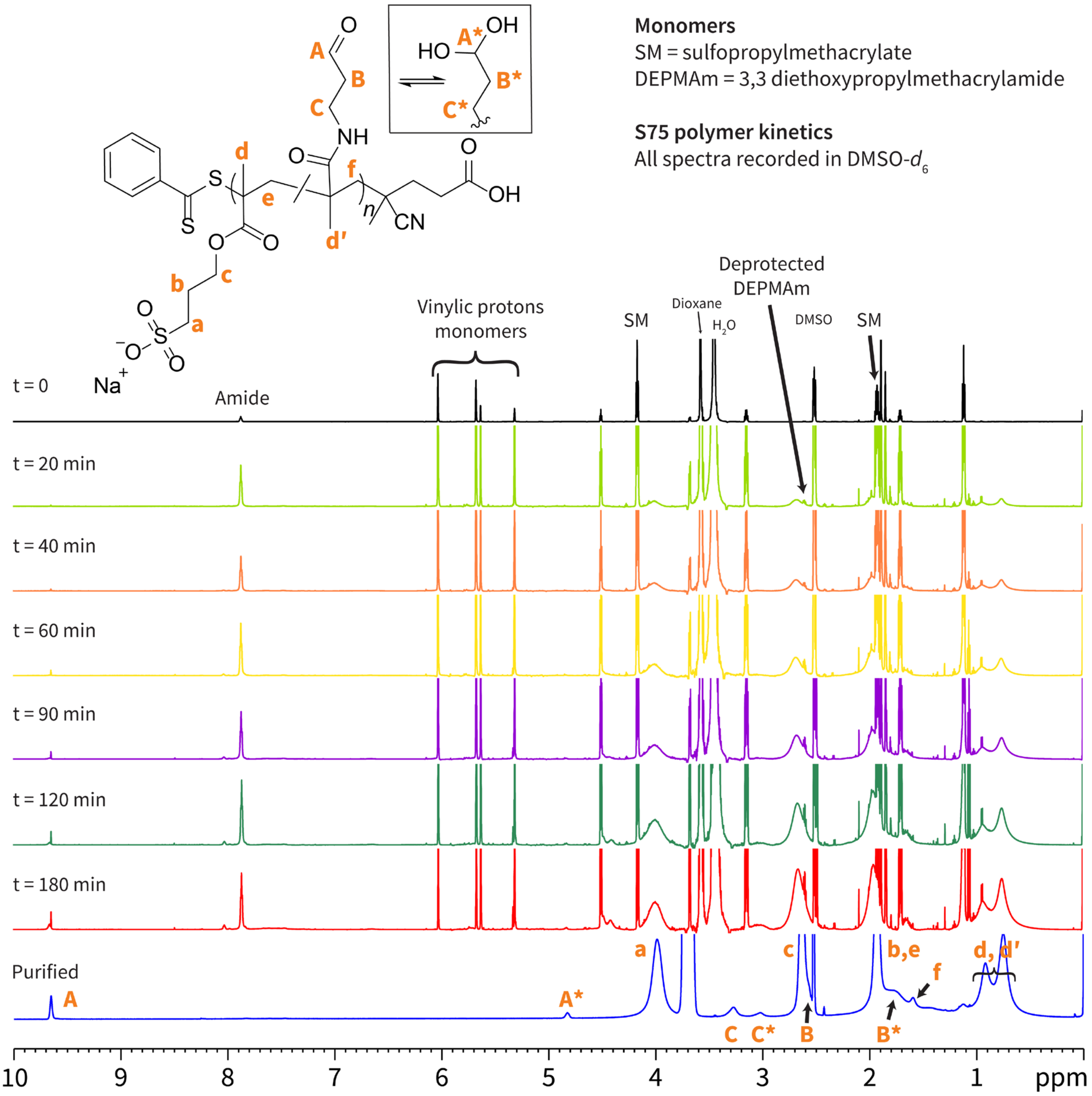

Figure S8. Time-course ^{1}H NMR (700 MHz in DMSO-d_6) spectra monitoring the copolymerization of SM and DEPMAm with a feed ratio of 75:25 mol % (**S75**), respectively. Based on the ratio of the newly apparent ethyl peak of ethanol (≈ 1.06 ppm, first visible after the start of the reaction) to the methyl peak of the acetal protecting group (≈ 1.11 ppm), we estimated the amount of *in situ* deprotection to be ≈ 25% after 180 min. To obtain the purified spectra in DMSO, the polymer was pre-dissolved in D_2O (≈ 10% of the total volume), which led to the disappearance of the amide peak and acetal formation with the free aldehyde.

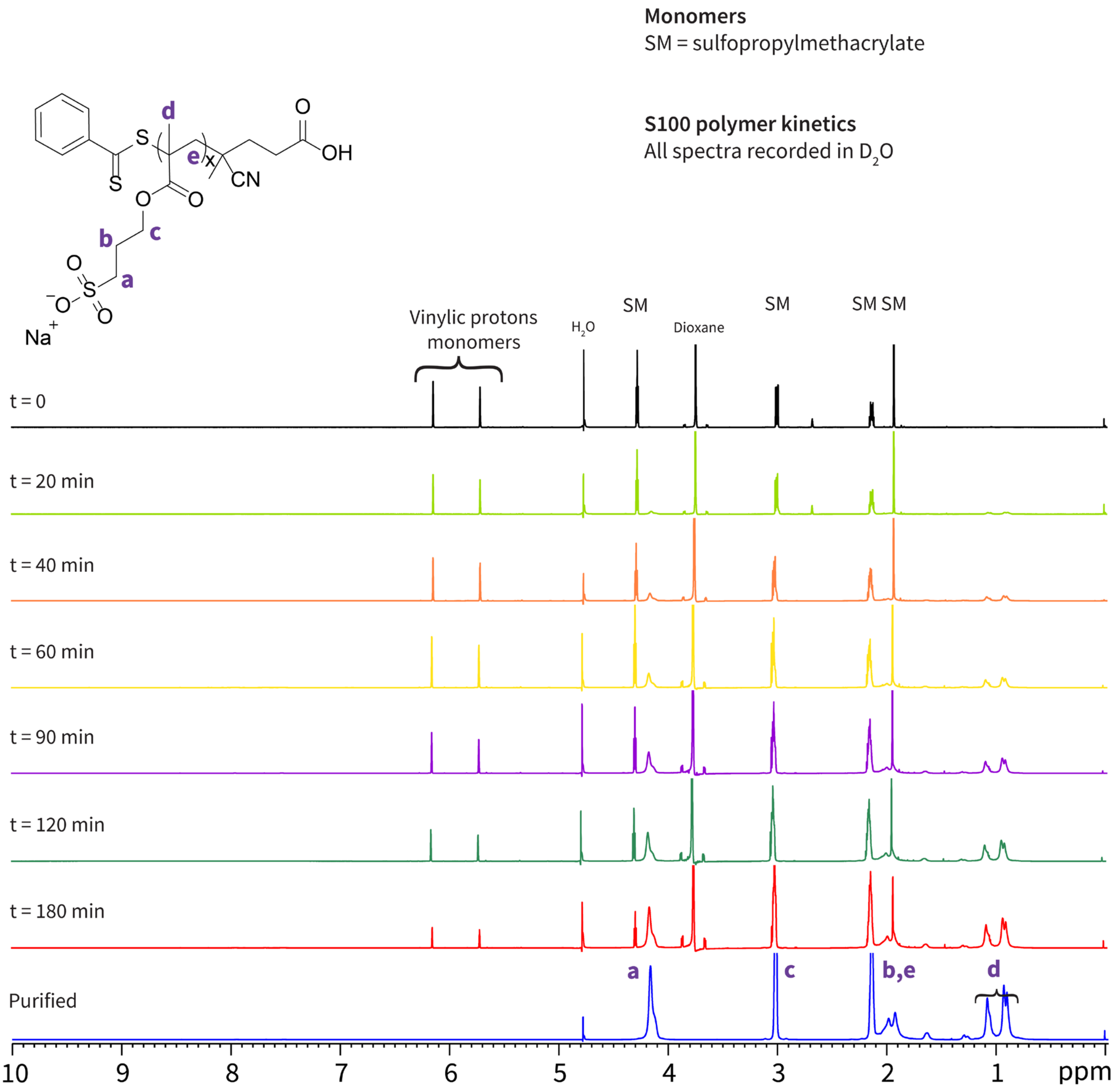

Figure S9. Time-course ^{1}H NMR (700 MHz in D_2O) spectra monitoring the homopolymerization of SM (**S100**).

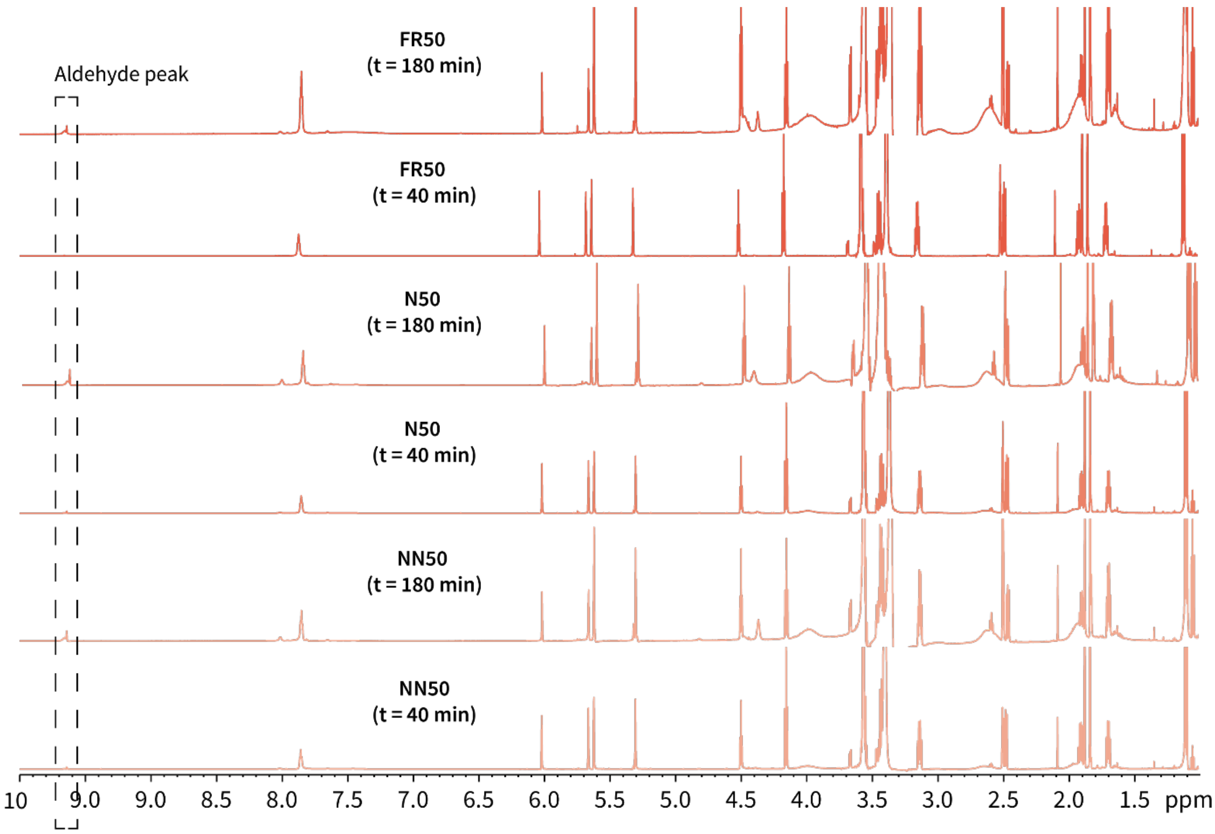

Figure S10. ^{1}H NMR (700 MHz in DMSO-d_6) spectra of crude reaction mixture (at t = 40 min and 180 min) of **NN50**, **N50**, and **FR50** (see also **Discussion S2**). In all reaction mixtures, rapid *in situ* deprotection of the aldehyde occurred as we observed a clear aldehyde peak (9.24 ppm) after 40 min, which became more prevalent over the course of the reaction. At t = 0, all spectra were (almost) identical to **S50** without the presence of a free aldehyde peak (**Figure S7**).

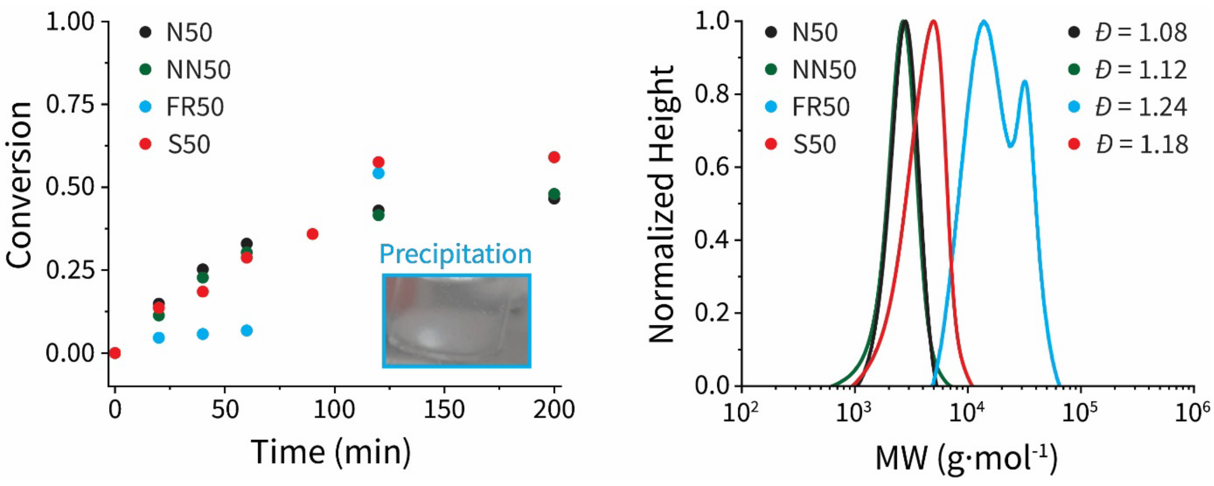

Figure S11. Analysis of **N50**, **NN5**0, and **FR50**. The data for the S50, reported in the main text, is also included for comparison. (Left) Monomer conversion over the course of the reaction as determined by NMR (Right) The final GPC traces for the purified products obtained for the same reactions highlighting the differences in molecular weight and dispersity.

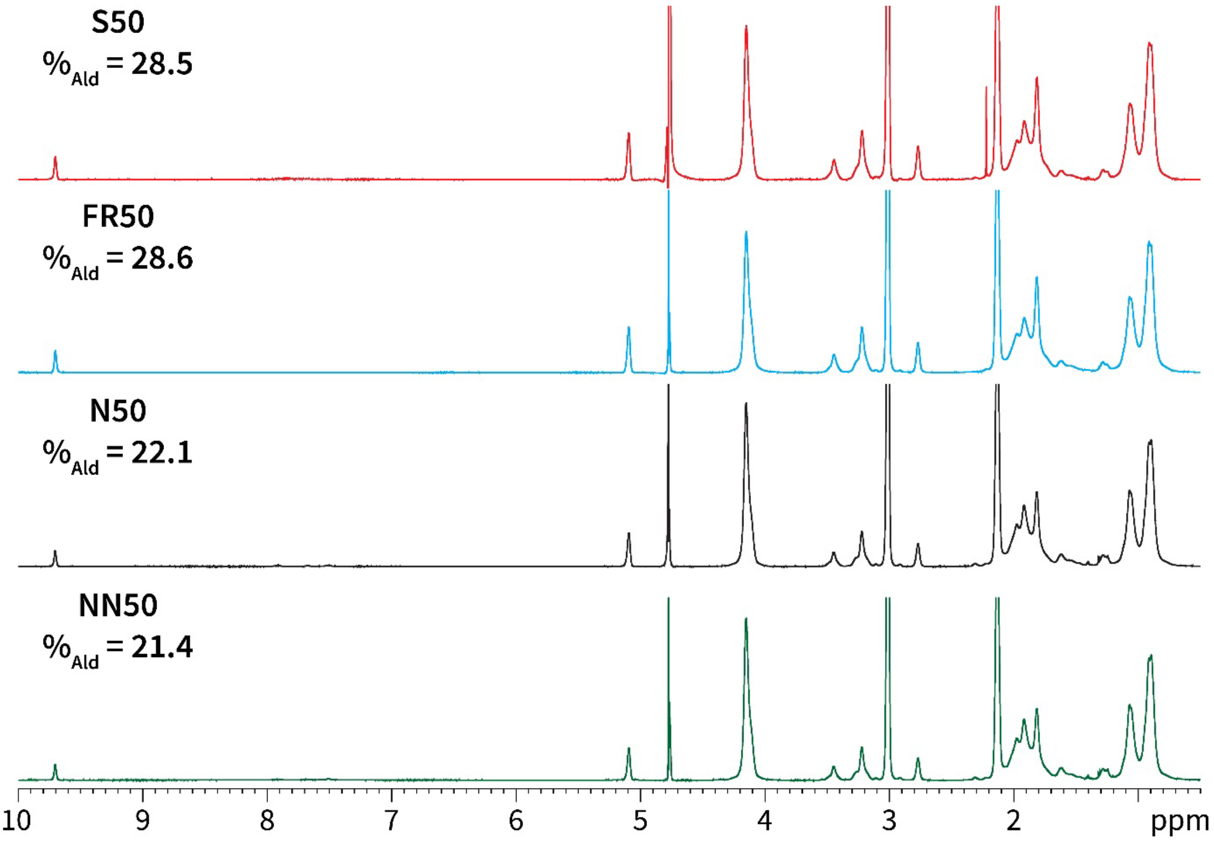

Figure S12. ^{1}H NMR (700 MHz, D_2O) spectrum of purified **N50**, **NN50**, and **FR50**. NMR data for **S50** from the main text is included for comparison.

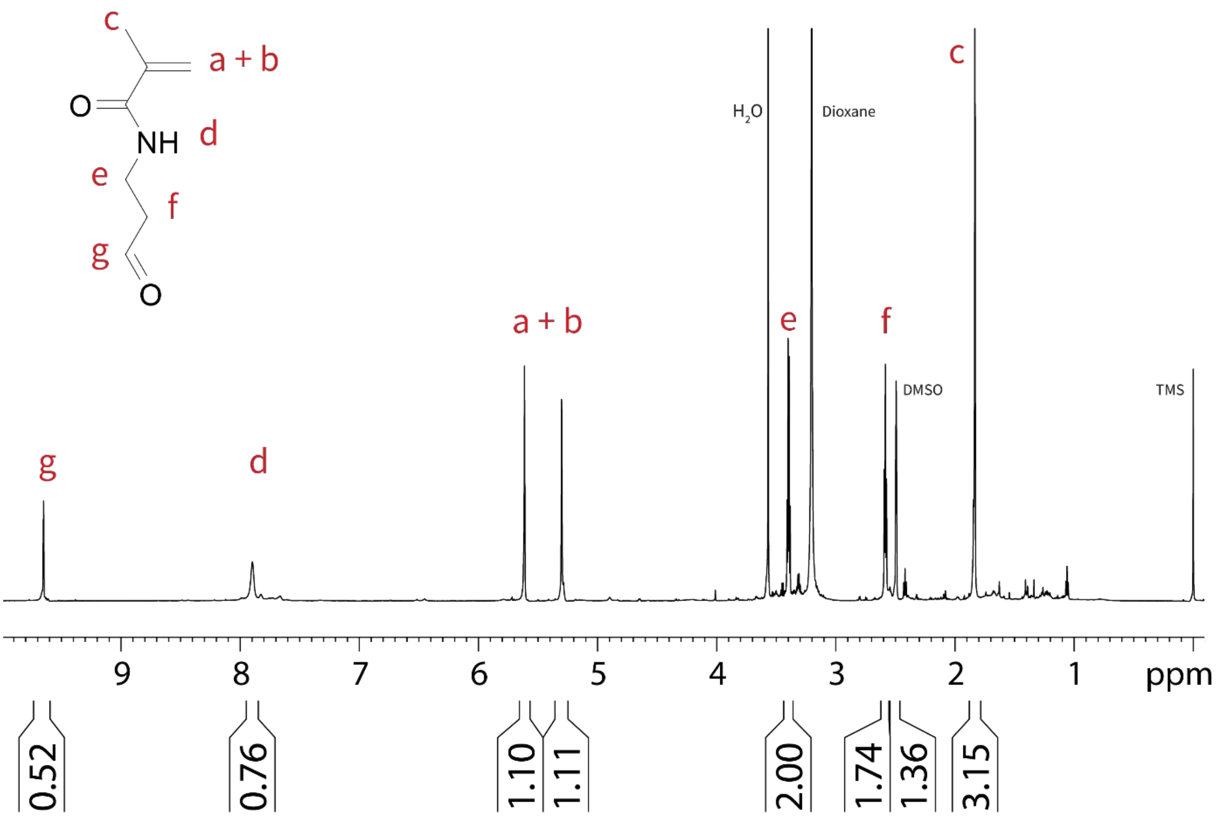

Figure S13. ^{1}H NMR (700 MHz, DMSO-d_6) spectrum of the soluble fraction of the concentrated crude reaction mixture of the deprotection of DEPMAm after extraction with DMSO-d_6 (see **Method S1**).

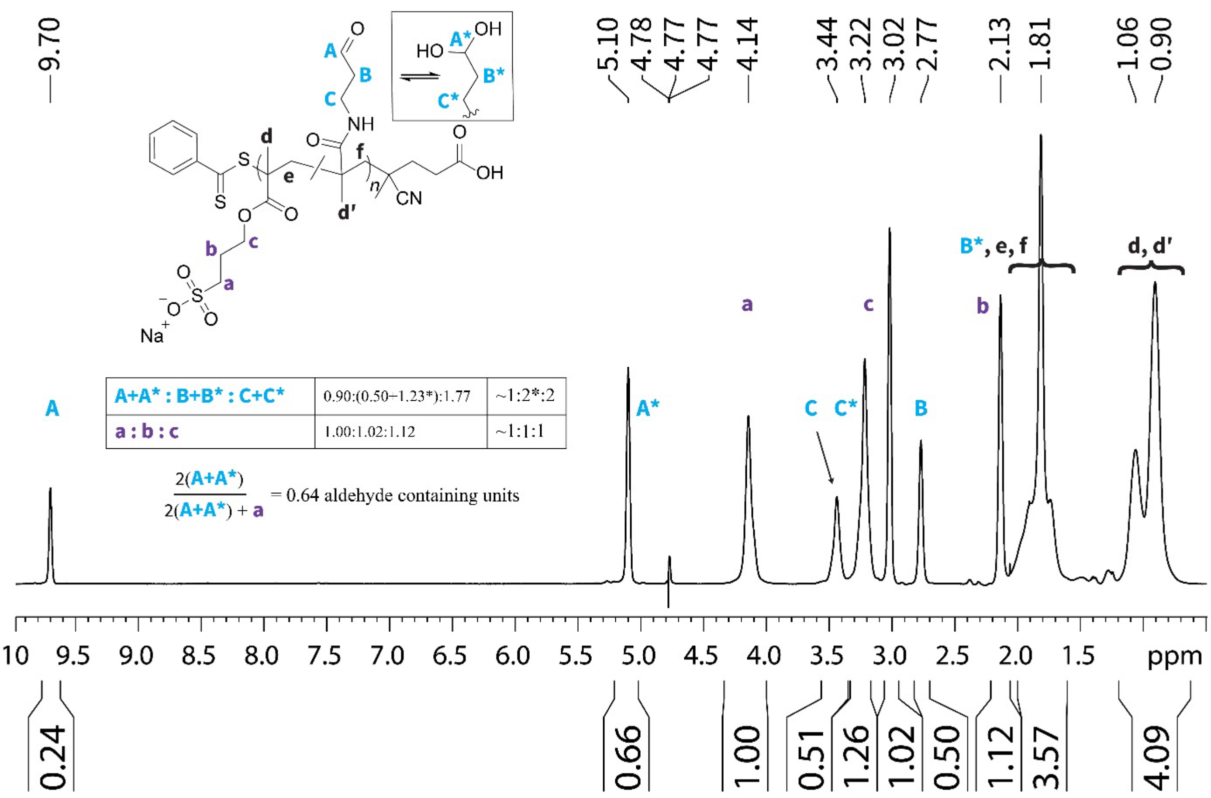

Figure S14. ^{1}H NMR (700 MHz, D_2O) spectrum of purified **S25** including the integration values. The integral ratios of the side chains align with the expected ratios. These ratios were also used to determine the composition of the backbone of the polymer. *We assumed that the ratio of B:B* equals the ratio of C:C*.

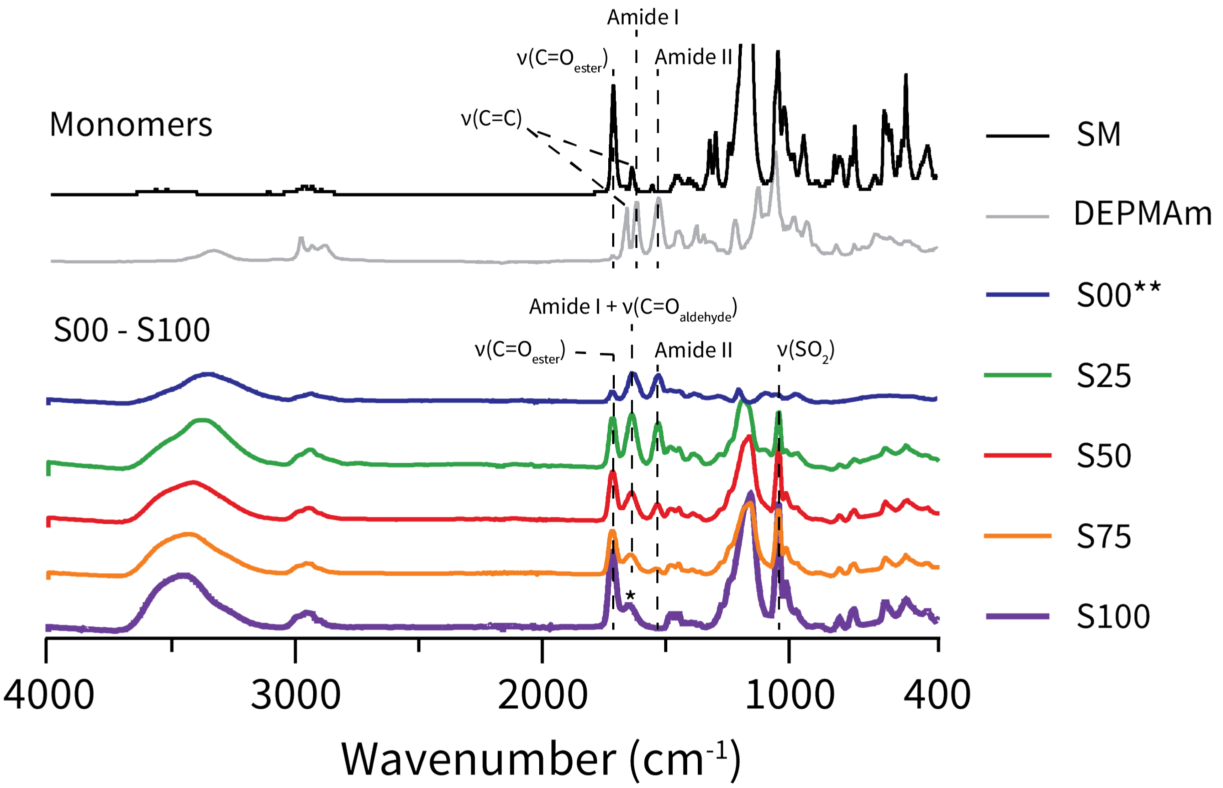

Figure S15. ATR-FTIR spectra of both monomers (SM & DEPMAm), and all (co-)polymers (**S00**–**S100**), with their main functional group assigned. The peak (1648 cm^{-1}) with an asterisk (*) in the **S100** spectrum does not align with the assigned amide I and aldehyde peak at 1637 cm^{-1} in the other polymer spectra. **In the **S00**, the assignment of the ester band should be interpreted with care, as we were unable to isolate the purified product due to the formation of side reactions (**Discussion S1**).

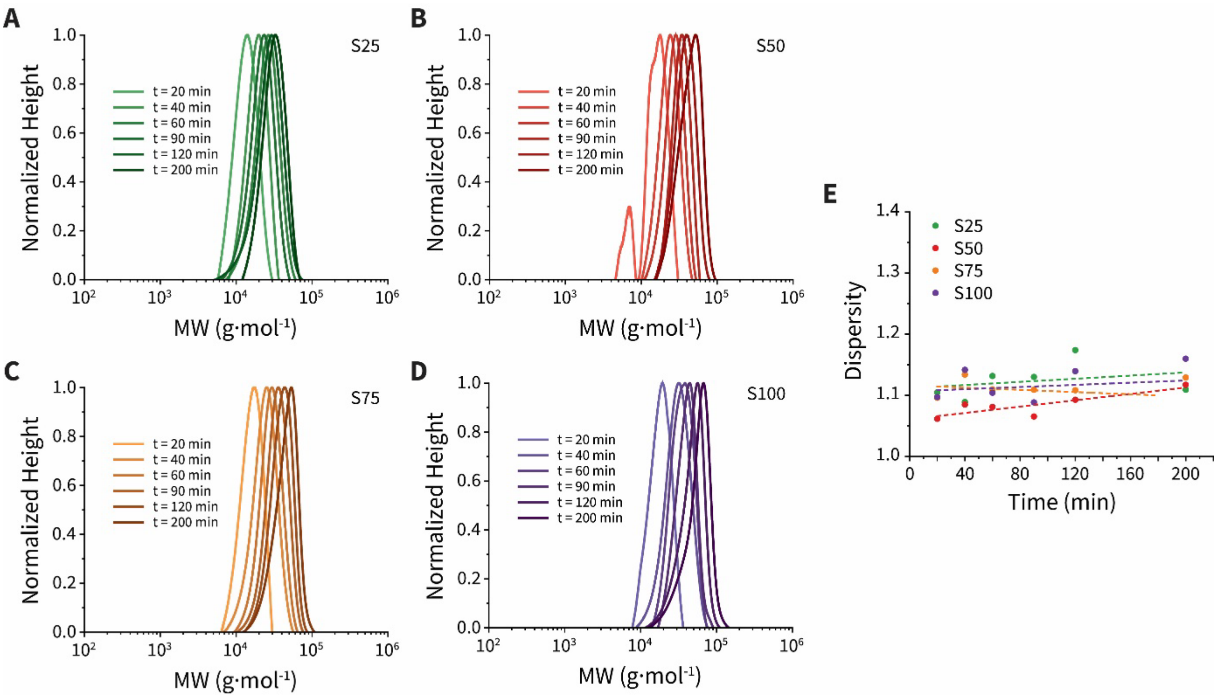

Figure S16. Time-course GPC traces over 200 min of the (co-)polymerization of SM and DEPMAm. (A) **S25**, (B) **S50**, (C) **S75**, (D) **S100**. (F) The dispersity over time for each feed ratio; linear fits have been added for visual clarity.

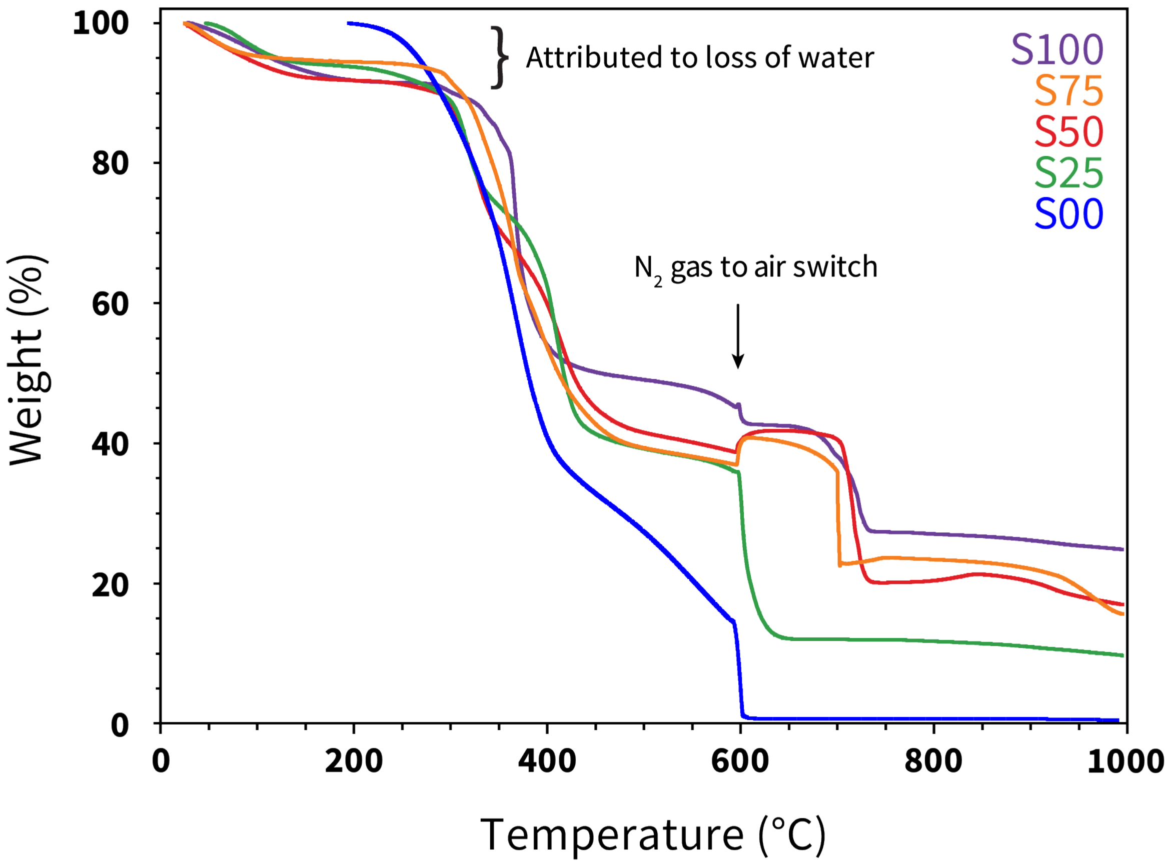

Figure S17. Thermogravimetric analysis (TGA) of **S00–S100** (co-)polymers.

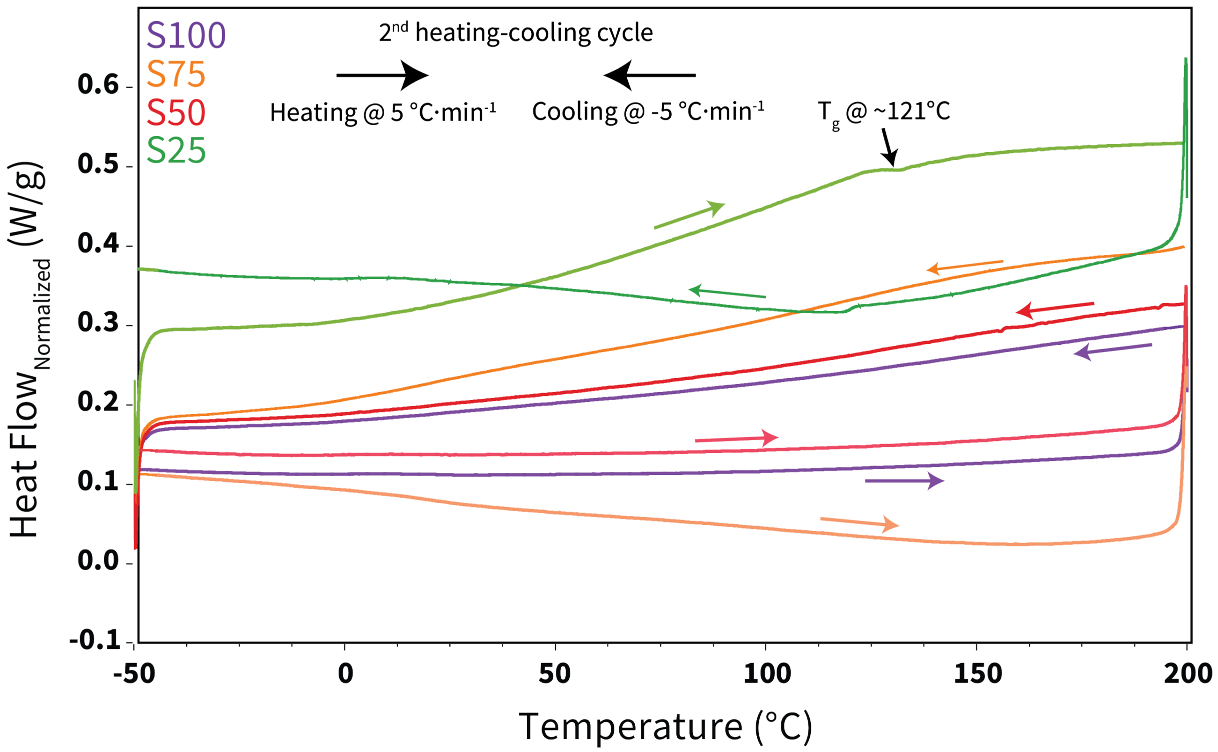

Figure S18. Differential scanning calorimetry (DSC) thermographic curves of **S25–S100**. After performing a first heating and cooling step to remove thermal history, we here depict the results of the second cycle. Measurements were performed from -50–200 °C heating (lighter curves) at 5 °C·min^{-1}, and cooling (darker curves) back to -50 °C at -5 °C·min^{-1}. n=1.

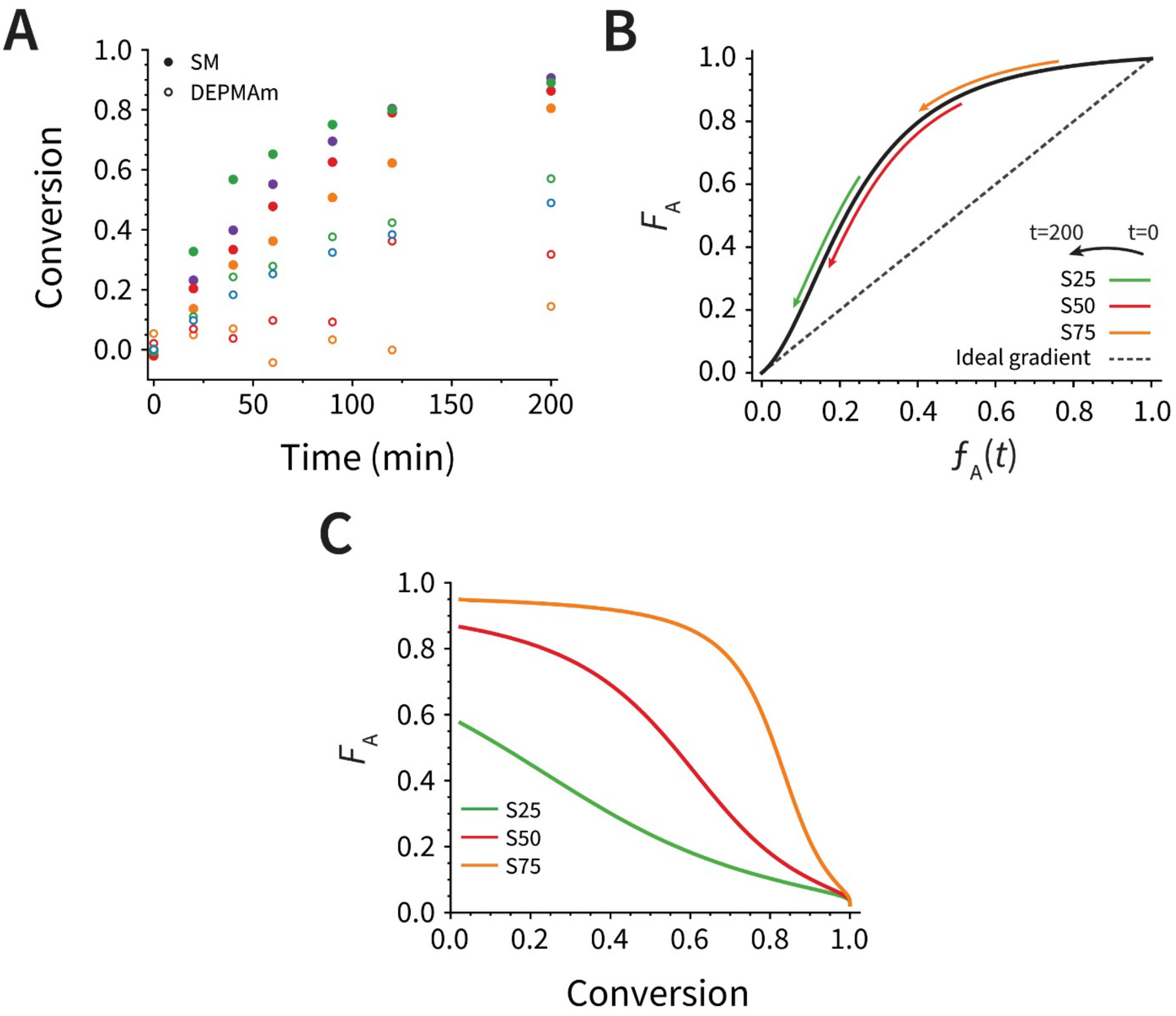

Figure S19. (A) Individual monomer conversions over time calculated according to **Equation S1**. (B) A plot of the instantaneous copolymer composition (F_A) as a function of the mole fraction of unreacted SM monomer. The dotted black line represents ideal gradient copolymerization while the black line is calculated using the r_A and r_B values determined in **Figure 3**. The colored line segments represent the evolution of instantaneous chain composition for each of the **S25–S75** copolymerization performed in this work. (C) Supporting plot of the instantaneous copolymer composition (F_A) as a function of overall conversion according to **Equation S2**.

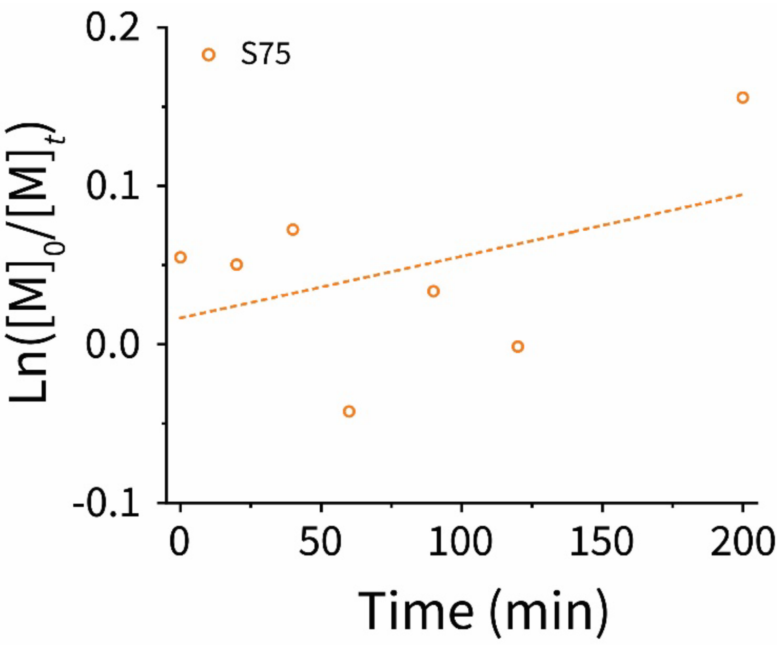

Figure S20. Pseudo-first order kinetic fit of $Ln([M]_0/[M]_t)$ versus time for DEPMAm in the **S75** copolymerization gives a large error as it contains both positive and negative small values clustered around zero.

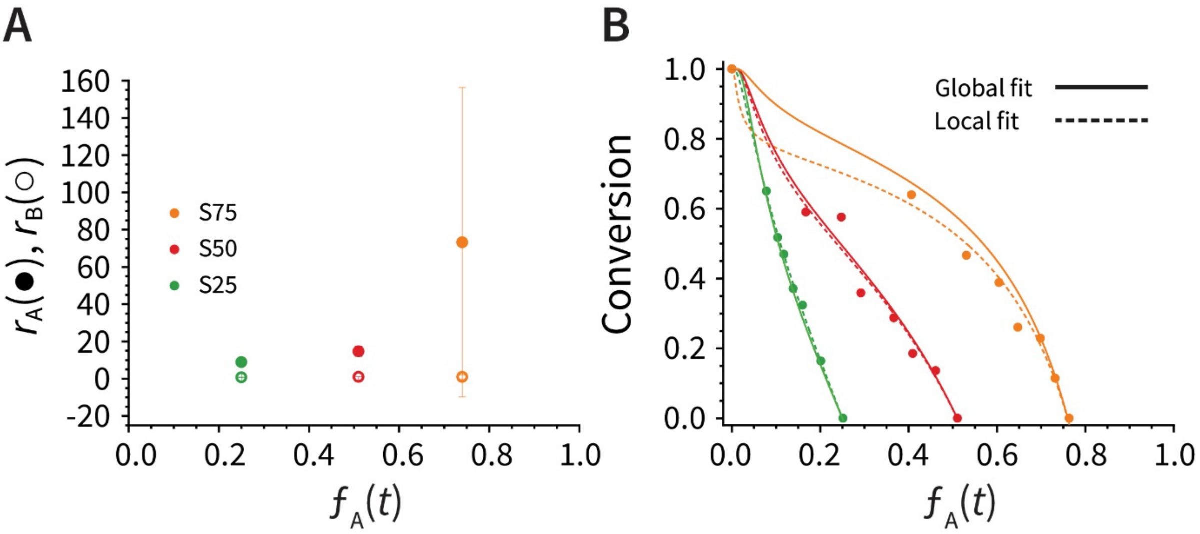

Figure S21. Individual and global fits to the Meyer-Lowry model (**Equation 1**) for the **S25–S75** copolymerization reactions and supporting plot of the instantaneous copolymer composition as a function of overall conversion. (A) Plot of the r_A and r_B values obtained for individual fits highlighting the error present in **S75**. (B) Comparison of the form of the individual and global fits for the **S25–S75** copolymerizations showing the consistency for **S25–S50** while **S75** begins to deviate at higher conversion with the uncertain individual fit.

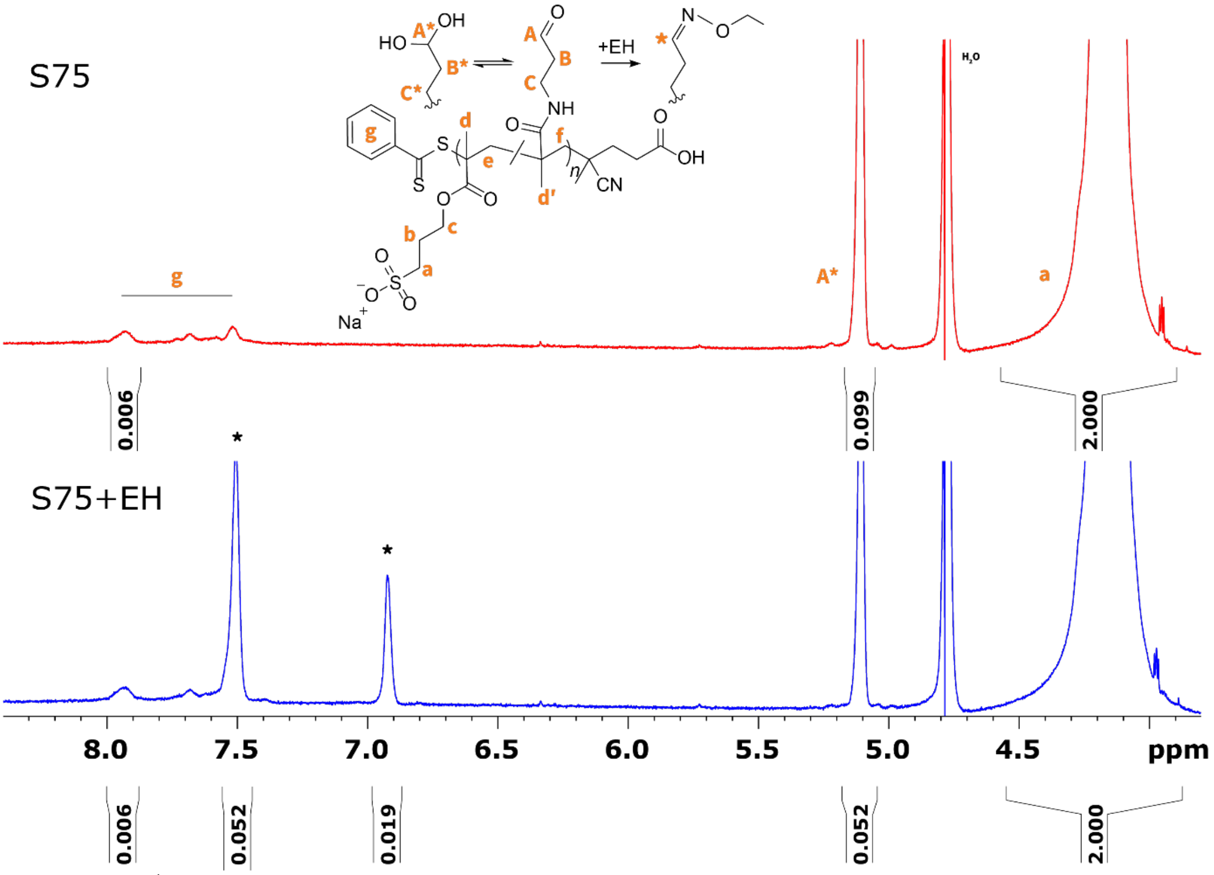

Figure S22. ^{1}H NMR (700 MHz, D_2O) spectra of S75 (top, red) and S75 mixed with 0.6 equiv w.r.t. aldehyde groups of *O*-ethylhydroxylamine (EH) for 2 days (bottom, blue). The spectra indicates that ≈ 50% of the aldehydes were converted into oxime bonds as indicated by the *E*/*Z* imine-type product (*) at ≈ 6.95 and ≈ 7.5 ppm. The CTA group did not apparently react with the hydroxylamine.

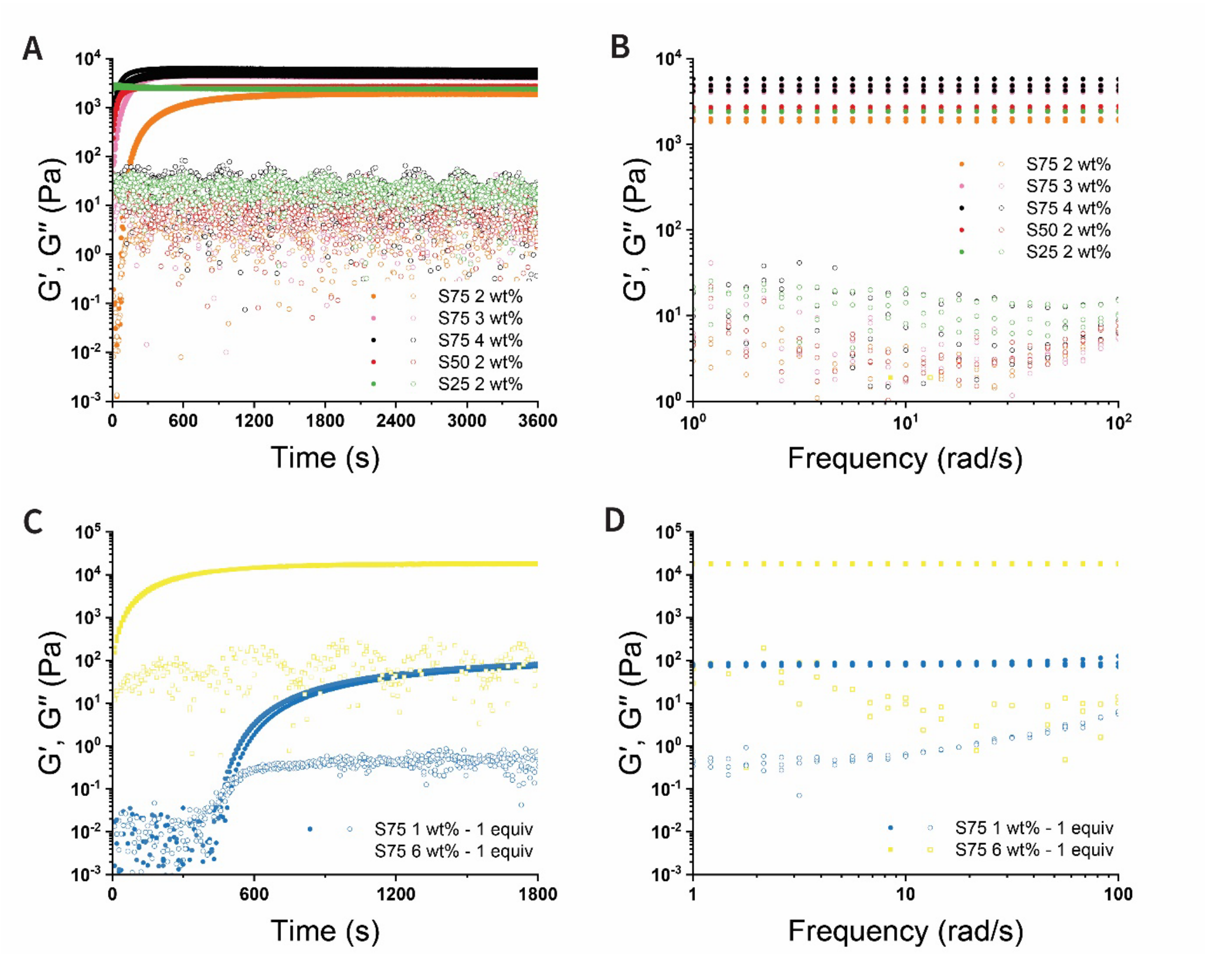

Figure S23. Full time sweeps (A+C) and frequency sweeps (B+D) of the **S25–S75** hydrogels varying either the copolymer wt% or the copolymer formulation.

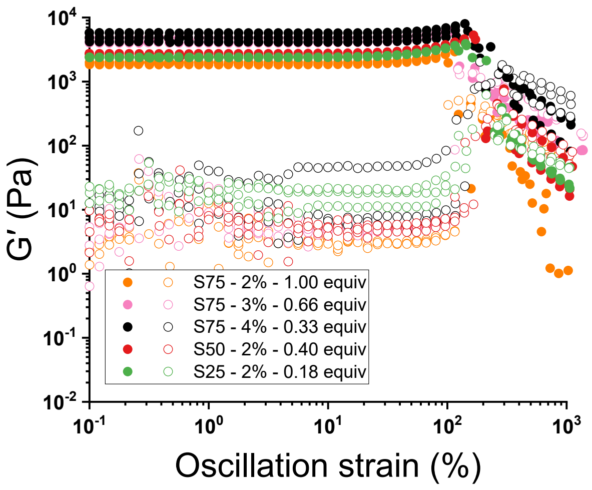

Figure S24. Full strain sweeps of the **S75–S25** copolymer hydrogels, varying either the copolymer wt% or the copolymer formulation.

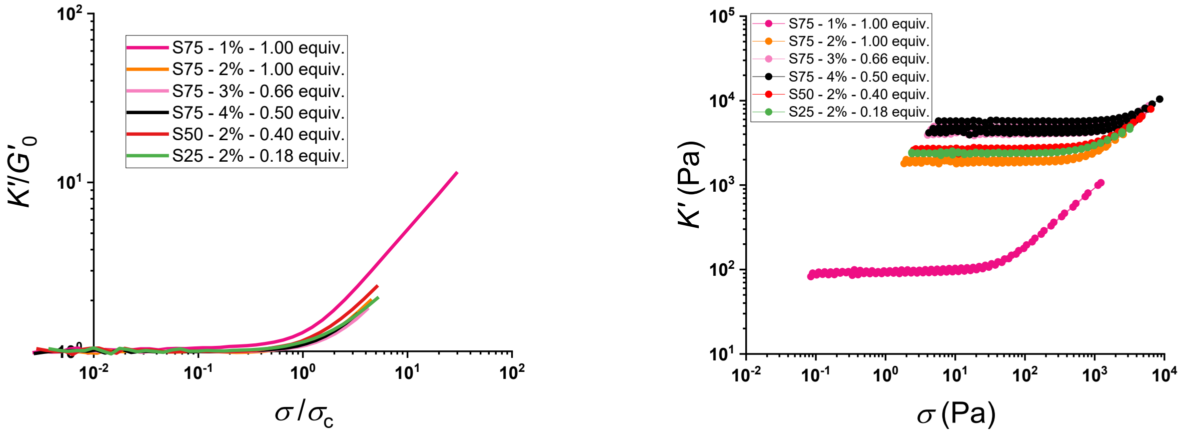

Figure S25. (Left) Representative plot of normalized differential modulus (K'/ K'_{lin}) vs normalized stress (σ/σ_c). (Right) Raw K' vs σ for all replicates.

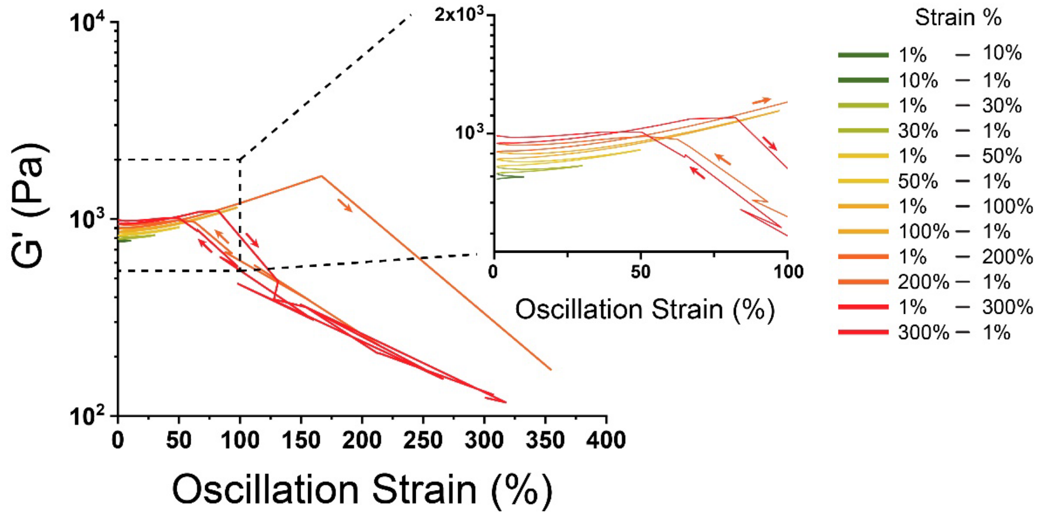

Figure S26. The strain response, rupture, and recovery, of an **S75** hydrogel (1.9 wt%, 1 equiv hydrazides from **PEG-HZ**) when subjected to cyclic strain from 1–300%.

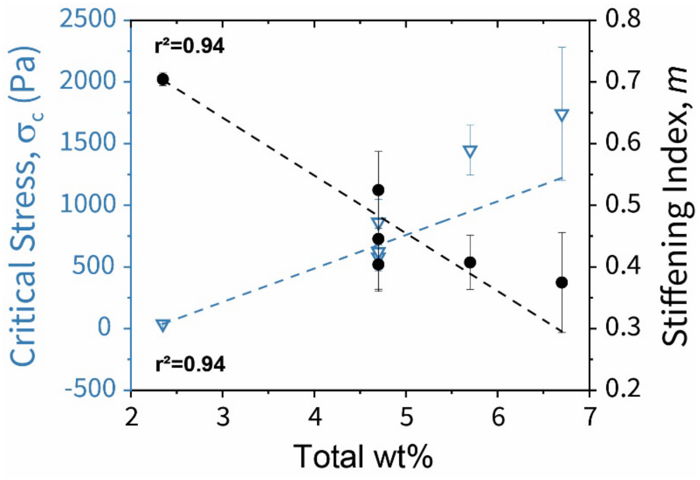

Figure S27. Plot of the critical stress (σ_c) and stiffening index (m) as a function of total wt% of the **S25–S75** hydrogels. For a plot of differential modulus (K') vs stress (σ), the critical stress is determined as the intersection of two linear regimes (**Figure S23**); the plateau, where $K' = G_0'$, and the stiffening regime (taken here as the final 5 points), where $K' = a\sigma^m$, and m is the corresponding stiffening index.

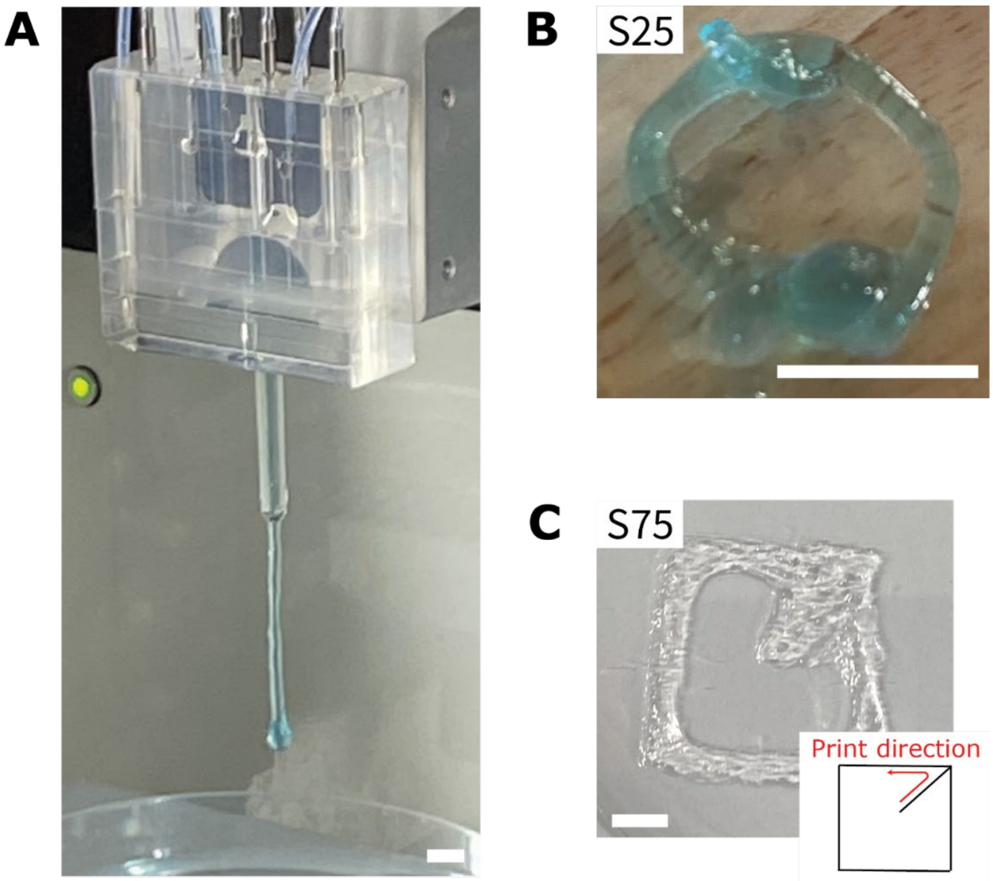

Figure S28. Proof of concept microfluidic printing of **S25** and **S75** with PEG-HZ. (A) Extrusion of a stable filament (**S25**) from the Aspect bioprinting. Scale bar = 1 mm. (B) Microfluidic print of a donut-shaped fiber using **S25** (9.5 wt%; 280 mM aldehyde) and PEG-HZ (13.2 wt%; 50 mM hydrazide) solutions. Scale bar = 1 mm. (C) A printed square (width = 40 mm) using **S75** (8.0 wt%; 40 mM aldehyde) and PEG-HZ (13.2 wt%; 50 mM hydrazide) solutions. Scale bar = 10 mm.

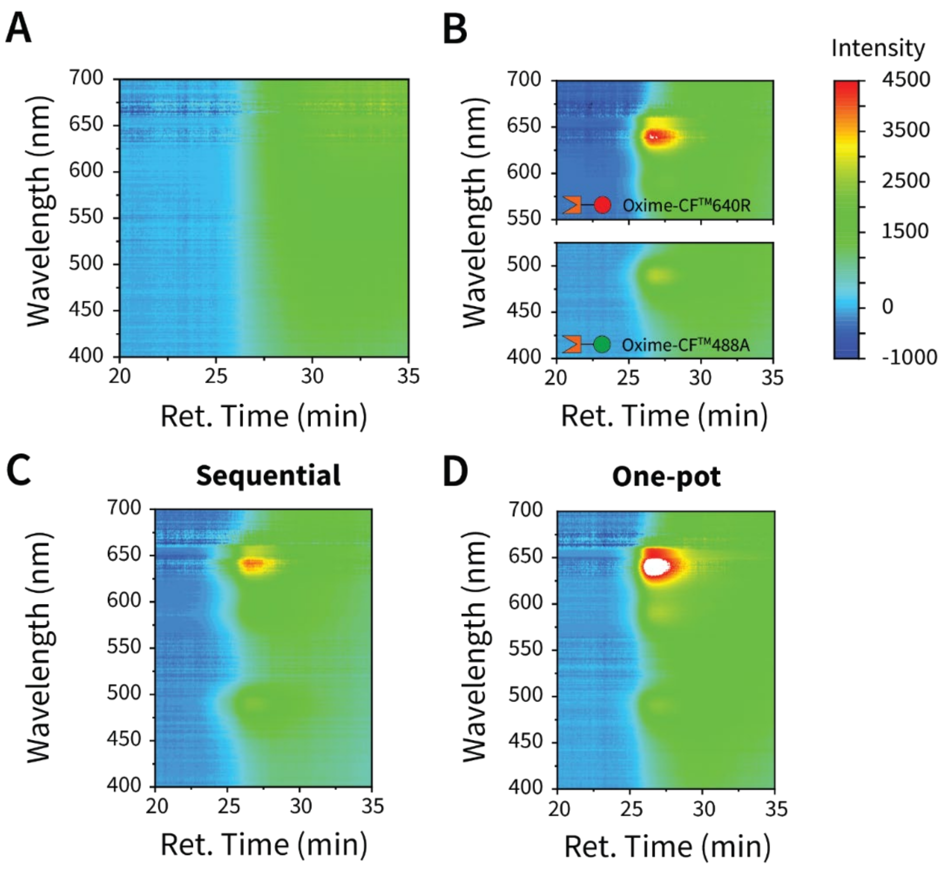

Figure S29. Absorption intensity vs retention time of **S50** prior to (A) and post conjugation with either Ox-CF488 (B, top, **S50+1**), Ox-CF640 (B, bottom, **S50+2**), or both dyes either sequentially (C, **S50+1-2**) or in a single reaction (D, **S50+O-P**).

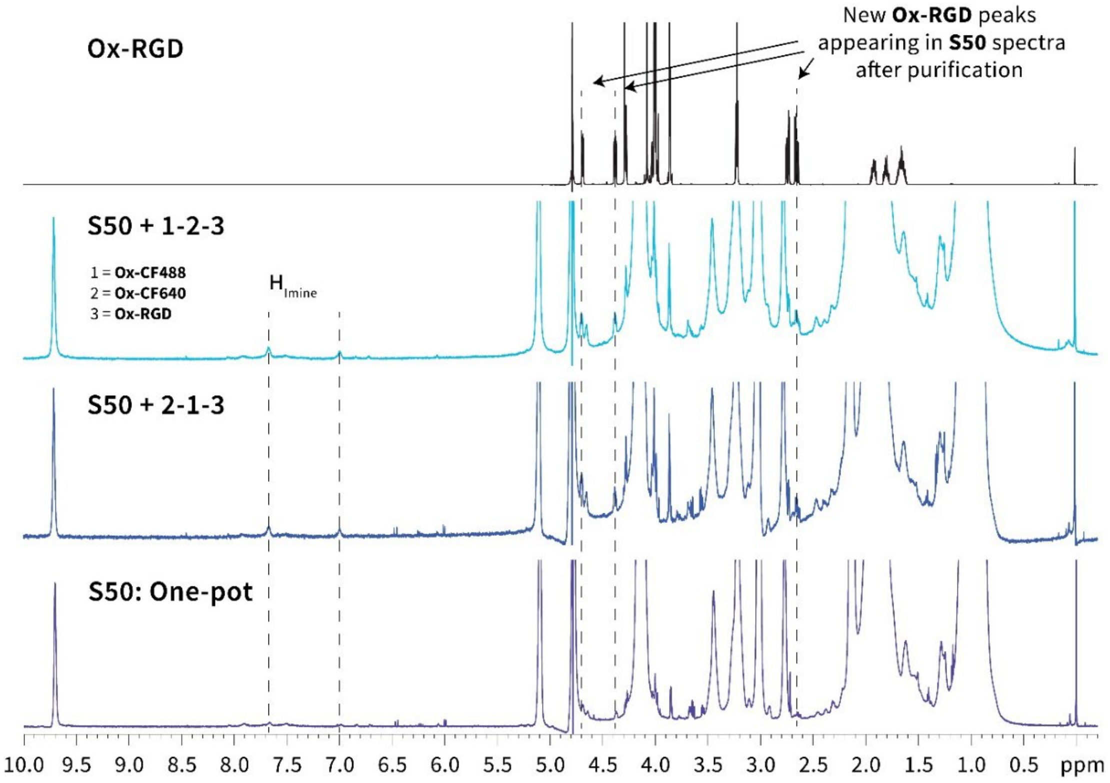

Figure S30. ^1H NMR (700 MHz in D_2O) spectra of Ox-RGD, and decorated **S50** after purification. A sequential and one-pot approach was used to conjugate ligands (see **Figure S23**). One can note the appearance and broadening of the Ox-RGD peaks in the **S50** spectra, due to binding to a polymer. Furthermore, the *cis*- and *trans*- conformations of the imine protons appear at 7.0 and 7.7 ppm.

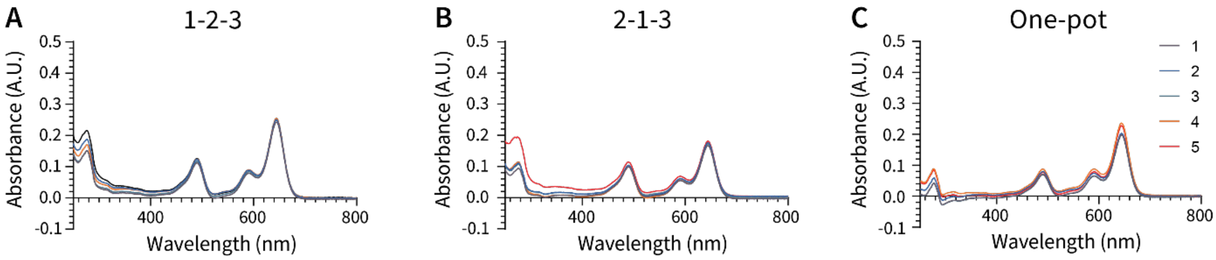

Figure S31. Absorbance of purified **S50** copolymer with conjugated Ox-CF488 (1), Ox-CF640 (2), and Ox-RGD (3). We attempted a sequential (A) 1-2-3, (B) 2-1-3, and one-pot (C) approach to decorate the copolymer. The S50 without conjugated molecules was used to correct the background. Five measurements were done from two stock solutions. N=2, n=5.

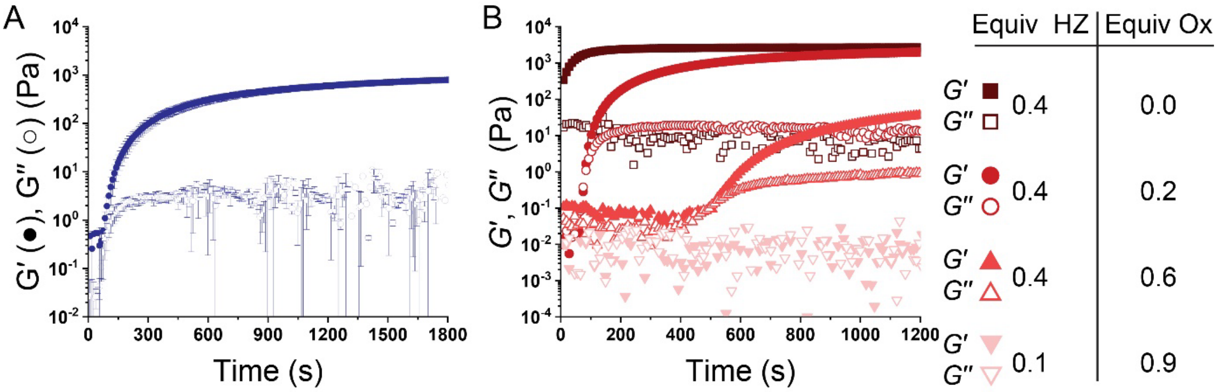

Figure S32. (A) Gelation kinetics of the functionalized **S50** copolymer at 1.5 wt% with 18.8 mM (1.0 equiv) hydrazide crosslinker. (B) Gelation kinetics of a series of 2 wt% S50 hydrogels with an increasing degree of pre-functionalization with *O*-ethylhydroxylamine.

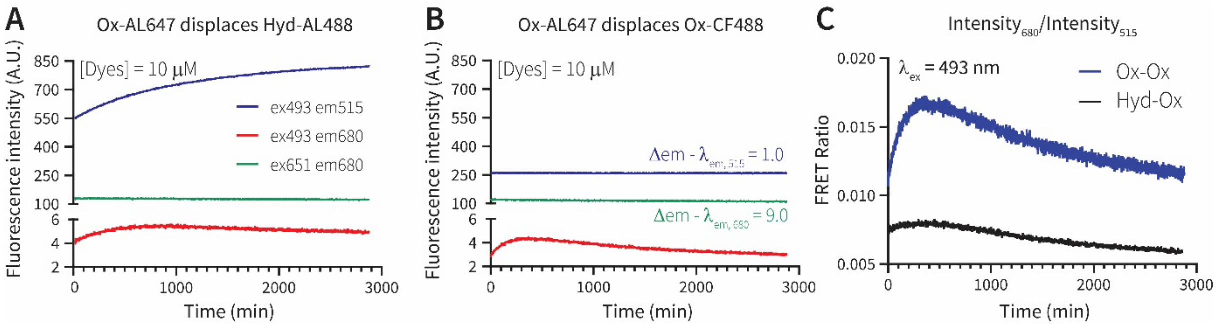

Figure S33. (A+B) Raw data following the evolution of the donor (λ_{ex} = 493 nm, λ_{em} = 515 nm), acceptor (λ_{ex} = 651 nm, λ_{em} = 680 nm), and FRET (λ_{ex} = 493 nm, λ_{em} = 680 nm) signals over ≈ 49 h. Δem was added in graph B to indicate the small difference in intensity between the end and begin of the signal. (C) The change in FRET ratio, defined here as the ratio of emission intensity at 680 nm (acceptor, em_{680}) to 515 nm (donor, Hyd-AL488 em_{515}) upon excitation at 493 nm: FRET ratio = em_{680}/em_{515}. Of note, due to drifting of the emission signal, the FRET ratio should be interpreted with care.

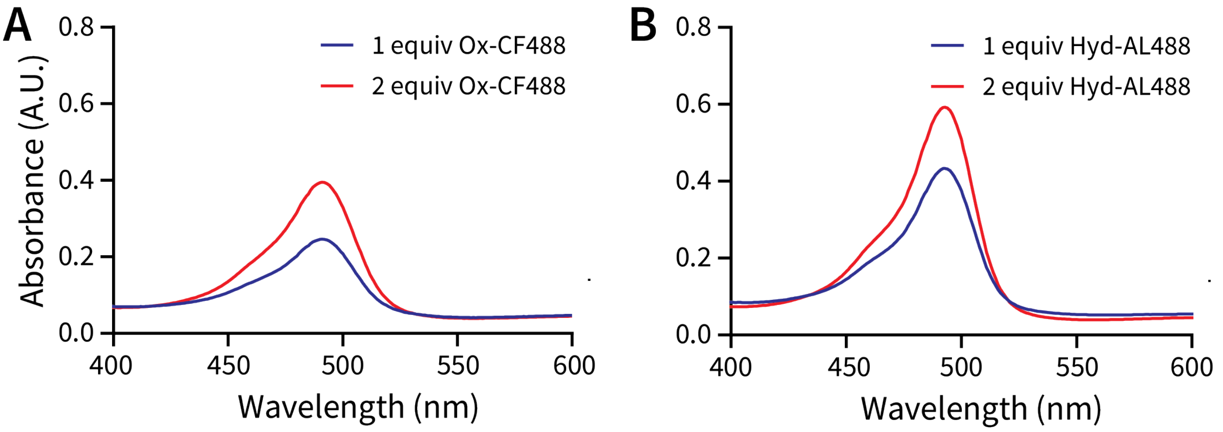

Figure S34. UV-Vis spectra of **S25** after mixing the polymer with either one or two equivalents of either (A) Ox-CF488 or (B) Hyd-AL488 for 3–4 h. The spectra were recorded after performing dialysis (3 buffer changes) against water.

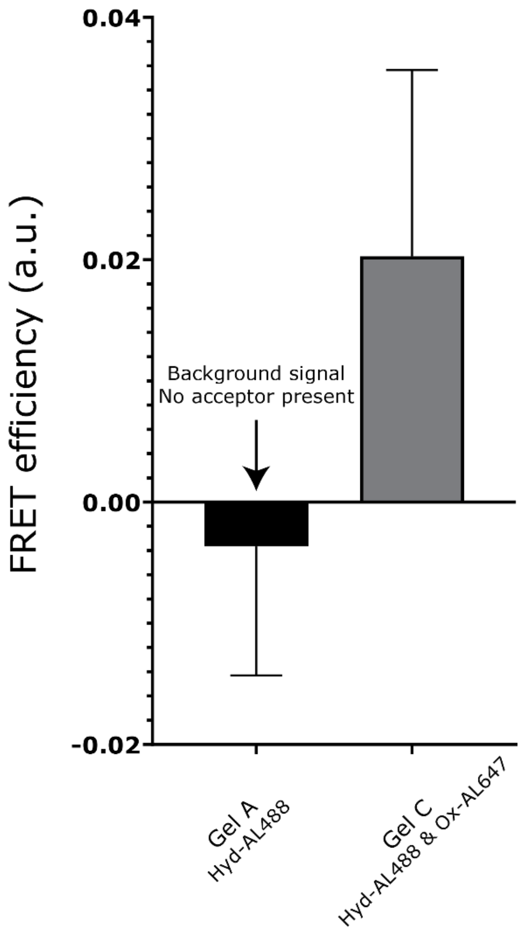

Figure S35. FRET efficiency data of **S25** hydrogels containing conjugated (Gel A) Hyd-AL488 or (Gel C) Ox-AL647 and Hyd-AL488 (see **Supporting Methods** for the gel labeling and preparation details). The FRET efficiency was measured to obtain a baseline measurement in the absence and presence of an acceptor (n = 2). However, the background (Gel A) and FRET (Gel C) signal possess too large of an overlap when considering their error for reliable measurements.

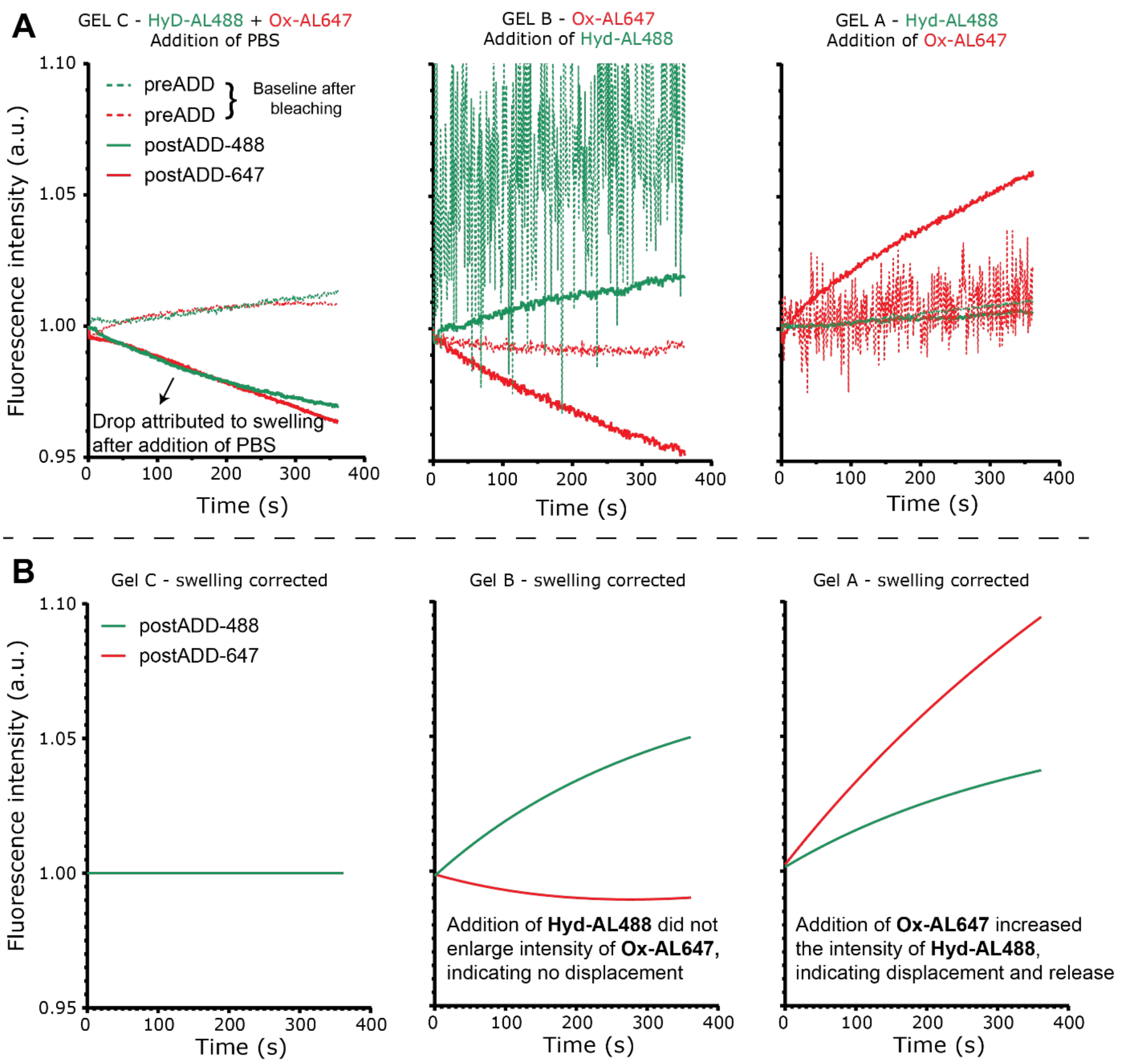

Figure S36. FRAP measurements indicate changes in diffusion of dye as result of displacement. (A) Raw FRAP data in **S25** hydrogels containing conjugated Ox-CF647 and Hyd-AL488 (Gel C, left), Ox-CF647 (Gel B, middle), or Hyd-AL488 (Gel A, right). After bleaching, the fluorescence intensity was set to 1.0 and the recovery was measured. To obtain a baseline, we measured the FRAP signal of Hyd-AL488 (λ_{em} = 500–550 nm, green) and Ox-CF647 (λ_{em} = 675–725 nm, red) on all three hydrogels (preADD). The baseline measurement indicated that before addition of a competitor no apparent changes in the recovery speed could be observed (signal remained constant around 1.00). Subsequently, we added PBS, Hyd-AL488, and OX-CF647, respectively, to the pre-formed gel C–A, and again measured the recovery (postADD). The addition of only PBS to Gel C led to a drop in the signal, which we attributed to diluting effects as result of swelling. (B) Therefore, we converted the raw data of the postADD into curves using non-linear regression, and corrected the postADD-488/647 data for swelling using the corresponding postADD graphs of gel C. An intensity going above 1.00 would suggest a signal due to FRAP recovery.

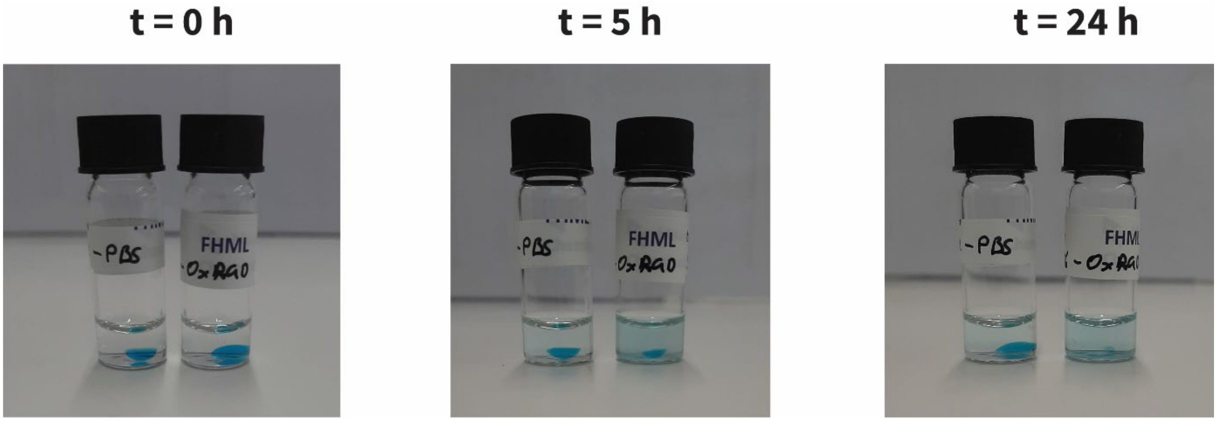

Figure S37. Bulk de-crosslinking of **S25** hydrogel via addition of excess Ox-RGD. A proof of concept study using a 1 wt% **S25** hydrogel, crosslinked with 0.2 equiv PEG-HZ and loaded with 0.001 equiv Ox-AL647 for visual clarity, is stable in PBS (left vial), but de-crosslinked via competitive displacement in the presence of ≈2 equiv Ox-RGD (right vial).

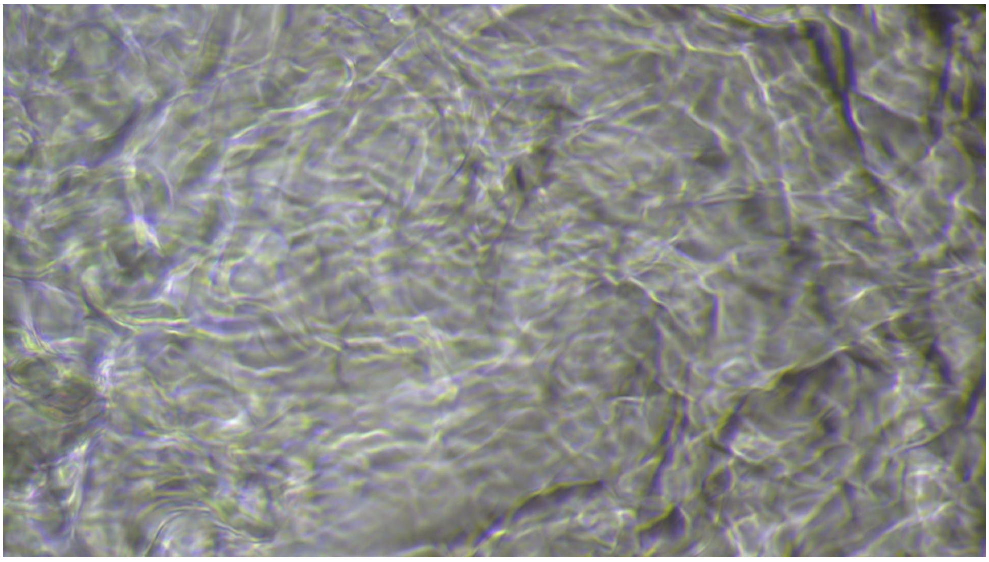

Figure S38. A 2 wt% **S25** polymer was crosslinked using 0.18 equiv of PEG-HZ in a 96 well plate. A low magnification (4x) using brightfield microscopy displays the clear refractive pattern induced by the hydrogel.

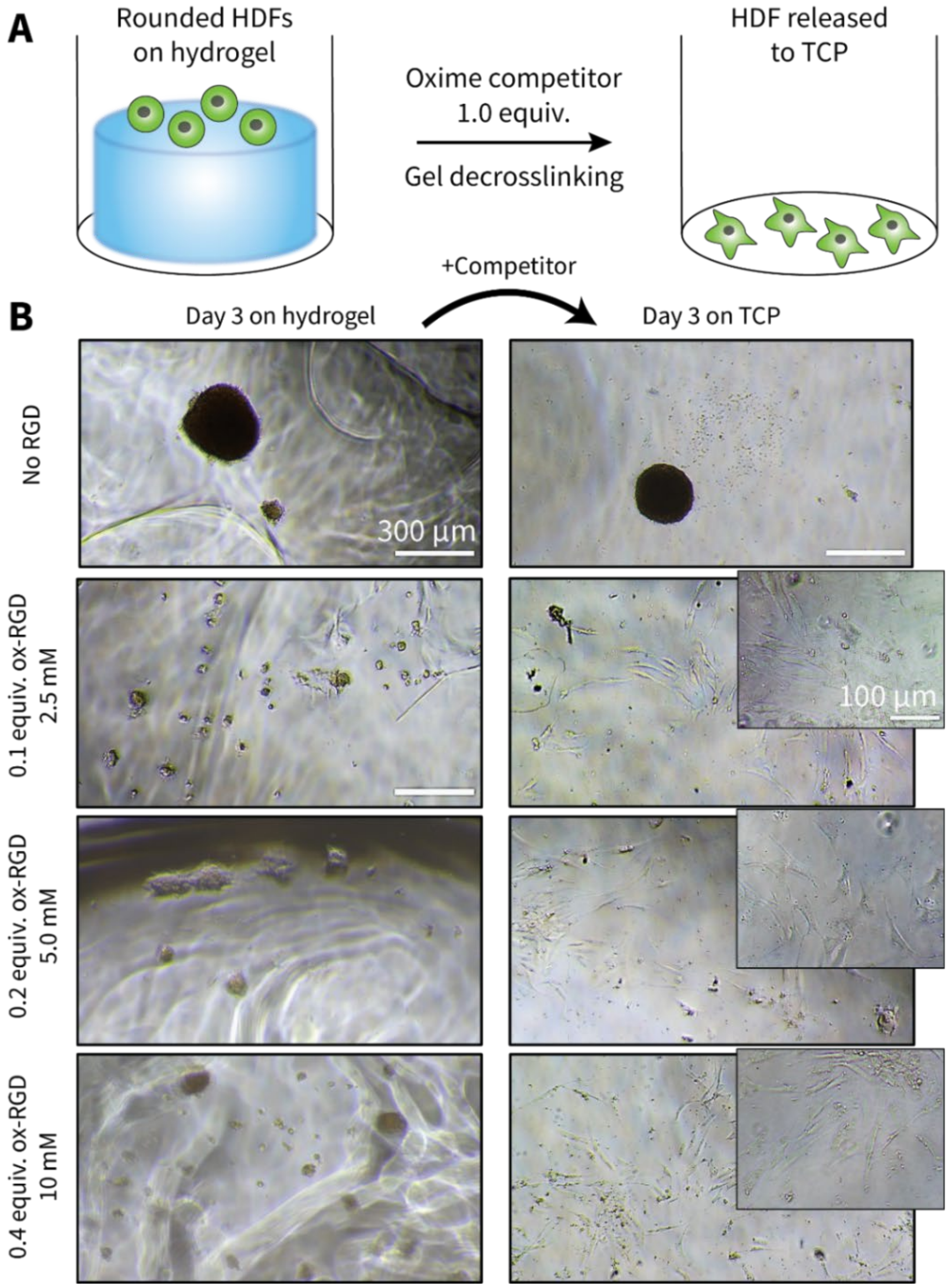

Figure S39. Proof-of-concept study using competitive displacement upon addition of a hydroxylamine small molecule to de-crosslink **S50** hydrogels and release cells to the TCP. (A) Illustration of the set-up for this study. Cells are cultured on **S50** hydrogels for 3 days. On day 3, an oxime competitor (*O*-ethylhydroxylamine) is added to decrosslink the hydrogel, releasing the cells to the underlying TCP. The TCP was subsequently imaged 3 days later. (B, left) HDFs were cultured for 3 days on hydrogels without (No RGD, 0.8 equiv PEG-HZ) or with RGD (2.5 mM, 5.0 mM, or 10 mM Ox-RGD, 0.4 equiv PEG-HZ). On day 3, 1.0 equivalent (25 mM) of *O*-ethylhydroxylamine was added as a competitor in the fresh media to decrosslink the hydrogels, which took about 7–8 h, and release the cells to the TCP. (B, right) After 3 more days on TCP (so 6 total culture days), the TCP was inspected using microscopy. Scale bars of the low magnification overview images are 300 µm and 100 µm in the zoom-in insets. *N* = 2.

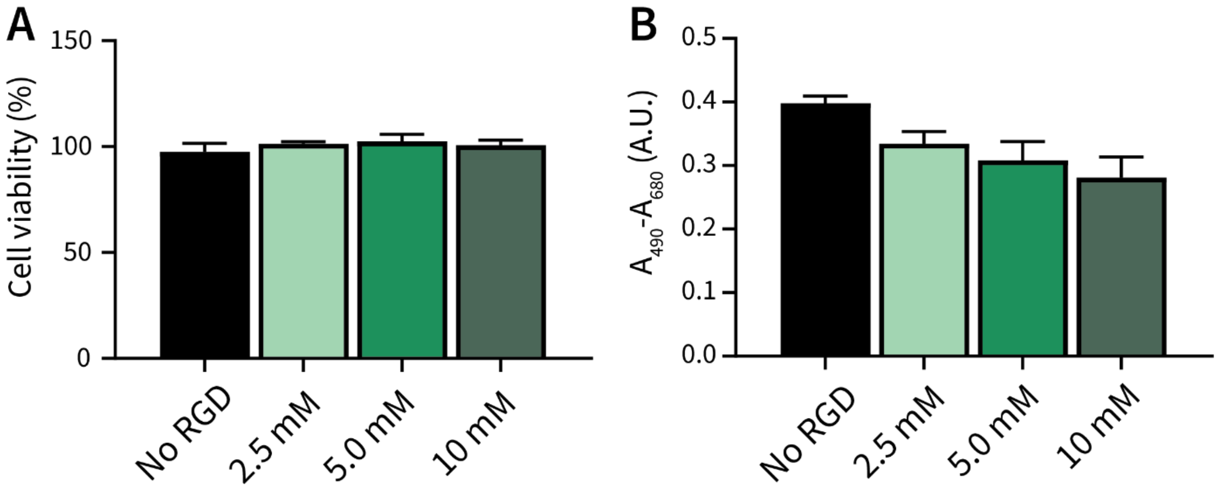

Figure S40. LDH cytotoxicity assay of HDFs seeded on S50 hydrogels containing 0, 2.5, 5.0, or 10 mM Ox-RGD. (A) HDF viability after 24 h on top of hydrogels. (B) Relative LDH activity of HDFs after 48 h on top of the hydrogel, determined by subtracting the absorbance at 680 nm from 490 nm. Due to the absence of the maximum LDH release after 48 h, we were not able to calculate the cell viability. In graph B, a lower value of (A_{490}-A_{680}) indicates a higher relative viability.

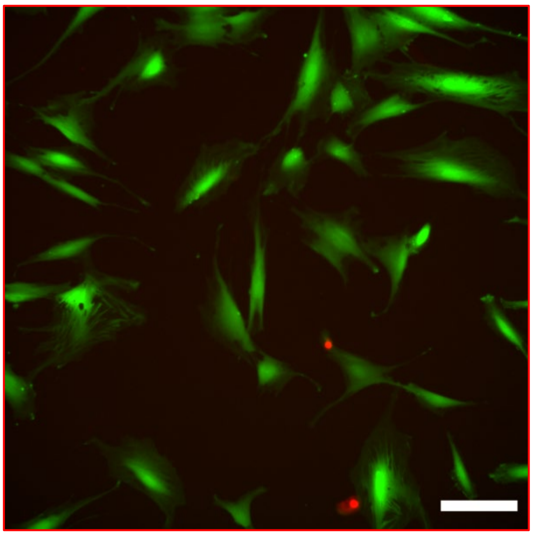

Figure S41. Live (calcein-AM, green) and dead (ethidium homodimer-1, red) image of HDFs on TCP after 24 h. This image serves as a positive control for cell adhesion and staining. Scale bar = 100 µm.

Supporting Tables

Table S1. Different solvents and conditions tested to dissolve the product obtained after dialysis and lyophilization of the **S00** reaction mixture.

Condition	Observation
dH_2O	Insoluble; white solid in solvent
DMSO	Insoluble; white solid in solvent
0.1 M HCl	Insoluble; white solid in solvent
dH_2O with a few drops of conc. H_2SO_4	Insoluble; white solid in solvent
Dioxane	Insoluble; white solid in solvent
HFIP	Insoluble; white solid became yellow after weeks
1.0 M NaOH	Insoluble; white solid became yellow overnight
Toluene	Insoluble; white solid in solvent

Table S2. Summary of conversion and copolymer characteristics for the small-scale neutralized RAFT and free radical **S50** copolymerizations; **S50** is included from the main text for comparison.

Reaction	Conv. (%)[a]	F_{Ald} (%)[b]	$M_{n,\,GPC}$ (kg·mol^{-1})[c]	$Đ$[c]
**S50**	59	28.5	36	1.18
**N50**	47	22.1	25	1.08
**NN50**	48	21.4	24	1.12
**FR50**	59	28.6	134	1.24

[a] The monomer conversion was determined via the ^{1}H NMR (700 MHz, DMSO-d_6) spectrum of the crude reaction mixtures at t = 180 min.
[b] The product was purified by dialysis and the incorporated fraction of backbone units containing free aldehyde was determined via ^{1}H NMR (700 MHz, D_2O).
[c] The M_n and dispersity of purified products was determined by GPC in aqueous 0.1 M $NaNO_3$.

Table S3. Screening of mass content and crosslinker equivalent to determine possible hydrogelation regimes via vial inversion.

pSM-*co*-OMAm	**S75** conc. (wt%)	[Aldehyde] (mM)	HZ-PEG$_{5k}$-ZH (equiv)	Gelation (Y/N)	Gelation onset
**S75**	10	50	1.0	Yes	<10 s
**S75**	10	25	0.5	Yes	<10 s
**S75**	10	10	0.2	Yes	<10 s
**S75**	4	20	1.0	Yes	<10 s
**S75**	2	10	1.0	Yes	<60 s

Table S4. Gelation onset times and plateau moduli for the **S25–S75** hydrogel formulations at different mass content and functionalization

**pSM-*co*-OMAm**	**wt% (copolymer)**	**wt% (PEG-HZ)**	**wt% (total)**	**Gelation Onset (s)**	**G_P (kPa)**
S75 1 wt%	1	1.35	2.35	≈480	0.08 ± 0.01
S75 2 wt%	2	2.70	4.70	≈60	1.9 ± 0.1
S75 3 wt%	3	2.70	5.70	< 8	4.5 ± 0.4
S75 4 wt%	4	2.70	6.70	< 8	4.9 ± 0.7
S75 6 wt%	6	8.10	14.10	< 8	18.0 ± 0.6
S50 2 wt%	2	2.70	4.70	< 8	2.6 ± 0.1
S25 2 wt%	2	2.70	4.70	< 8	2.4 ± 0.01

Table S5. Summary of critical stresses and associated stiffening indices.

**Formulation**	**Total wt%**	**σ_c (Pa)**	***m***
S75 1 wt%	2.35	40 ± 1	0.70 ± 0.01
S75 2 wt%	4.70	580 ± 110	0.45 ± 0.08
S75 3 wt%	5.70	1450 ± 200	0.41 ± 0.04
S75 4 wt%	6.70	1740 ± 540	0.37 ± 0.08
S50 2 wt%	4.70	860 ± 180	0.52 ± 0.06
S25 2 wt%	4.70	620 ± 50	0.40 ± 0.04

Table S6. Preparation of typical stock solutions for hydrogels in HDF release.

Compound	M_W (g·mol^{-1})	Mass (mg)	Amount (mmol)	Conc.[a] (mM)	V^b (μL)	Purity	ρ^c (g·cm^{-3})	wt%
**PEG-ZH**	5000	64.7	0.013	41.2	0.546[d]	0.95	1.15	10.3
**S50**	224	18.1	0.081	48.5	0.450	1	1.05	3.8
**S50+PAH**[e]	224	17	0.076	48.5	0.425	1	1.05	3.8

[a]Concentration of either the hydrazide or aldehyde group.
[b]Final volume has to be corrected for the change in density upon polymer addition; see *c*.
[c]Estimated density of the polymer in solution.
[d]The PEG-HZ salt was acidic, so a neutralization was required before further use. We prepared the stock solution by mixing 52 μL 0.5 M NaOH (1.0 equiv. relative to the hydrazide end-groups) and 494 μL PBS.
[e]We pre-conjugated 0.2 equivalents of aldehyde adding 0.38 μL of PAH and stirring the solution at RT overnight before further use.

Table S7. Hydrogel formulations for the HDFs release study.

Cond.	V_{S50} (μL)	Tot. [Ald] (mM)	V_{PEG-HZ} (μL)	Equiv. **PEG-HZ**	V_{PBS} (μL)	V_{Ox-RGD} (μL)	Equiv. **Ox-RGD**
**1**[a]	52.5	25.5	49.4	0.8	0.6	0	-
**2**[b]	52.5	25.5	24.6	0.4	20.4	5.0[c]	0.1
**3**[b]	52.5	25.5	24.6	0.4	15.4	10[c]	0.2
**4**[b]	52.5	25.5	24.6	0.4	15.4	10[d]	0.4

[a]**S50**+PAH (**Table S6**) was used.
[b]**S50** polymer (**Table S6**) was used.
[c]From a 50 mM Ox-RGD stock solution in PBS.
[d]From a 100 mM Ox-RGD stock solution in PBS.

Supporting Equations

Equation S1.

$$\text{Conversion} = 1 - \frac{[\text{M}]_t}{[\text{M}]_0} = 1 - \frac{[\text{A}]_t + [\text{B}]_t}{[\text{A}]_0 + [\text{B}]_0}$$

Equation S2.

$$F_\text{A} = \frac{r_\text{A} f_\text{A}^2 + f_\text{A} f_\text{B}}{r_\text{A} f_\text{A}^2 + 2 f_\text{A} f_\text{B} + r_\text{B} f_\text{B}^2}$$

Supporting References

(1) Kosinski, J. J.; Wang, P.; Springer, R. D.; Anderko, A. Modeling Acid-Base Equilibria and Phase Behavior in Mixed-Solvent Electrolyte Systems. *Fluid Phase Equilib.* **2007**, *256* (1–2), 34–41. https://doi.org/10.1016/j.fluid.2006.11.018.

(2) Girard, E.; Tassaing, T.; Marty, J. D.; Destarac, M. Influence of Macromolecular Characteristics of RAFT/MADIX Poly(Vinyl Acetate)-Based (Co)Polymers on Their Solubility in Supercritical Carbon Dioxide. *Polym. Chem.* **2011**, *2* (10), 2222–2230. https://doi.org/10.1039/c1py00209k.

(3) Homenick, C. M.; Sivasubramaniam, U.; Adronov, A. Effect of Polymer Chain Length on the Solubility of Polystyrene Grafted Single-Walled Carbon Nanotubes in Tetrahydrofuran. *Polym. Int.* **2008**, *57* (8), 1007–1011. https://doi.org/10.1002/pi.2439.

(4) Lynd, N. A.; Ferrier, R. C.; Beckingham, B. S. Recommendation for Accurate Experimental Determination of Reactivity Ratios in Chain Copolymerization. *Macromolecules* **2019**, *52* (6), 2277–2285. https://doi.org/10.1021/acs.macromol.8b01752.
